# Hydrofluorination of Alkynes Catalysed by Gold Bifluorides

**DOI:** 10.1002/cctc.201402891

**Published:** 2014-11-26

**Authors:** Fady Nahra, Scott R Patrick, Davide Bello, Marcel Brill, Alan Obled, David B Cordes, Alexandra M Z Slawin, David O'Hagan, Steven P Nolan

**Affiliations:** [a] EaStCHEM School of Chemistry, University of St AndrewsSt Andrews, KY16 9ST (UK)

**Keywords:** alkynes, bifluorides, gold, hydrofluorination, carbenes

## Abstract

We report the synthesis of nine new *N*-heterocyclic carbene gold bifluoride complexes starting from the corresponding *N*-heterocyclic carbene gold hydroxides. A new methodology to access *N,N′*-bis(2,6-diisopropylphenyl)imidazol-2-ylidene gold(I) fluoride starting from *N,N′*-bis(2,6-diisopropylphenyl)imidazol-2-ylidene gold(I) hydroxide and readily available potassium bifluoride is also reported. These gold bifluorides were shown to be efficient catalysts in the hydrofluorination of symmetrical and unsymmetrical alkynes, thus affording fluorinated stilbene analogues and fluorovinyl thioethers in good to excellent yields with high stereo- and regioselectivity. The method is exploited further to access a fluorinated combretastatin analogue selectively in two steps starting from commercially available reagents.

Fluorinated organic compounds are highly important in the pharmaceutical and agrochemical industries.[1] Incorporating a fluorinated group into drug-like compounds or biologically active molecules can improve many of their properties, including solubility, bioavailability, and metabolic stability.[2] The number of reports describing such chemistry has increased drastically over the last few years, giving rise to numerous procedures[3–9] catalysed mainly by Cu,[3] Pd,[4] Ir,[5] Ag,[6] Rh[7] and Mn[8] complexes.

The pioneering work of Sadighi, Gray and co-workers on the Au-catalysed hydrofluorination of alkynes revealed a new pathway for the easy formation of C=F bonds and, subsequently, the immense potential of *N*-heterocyclic carbene (NHC) gold(I) complexes in this field.[10]

Following reports by Riant, Leyssens and co-workers[11] and more recently by Huang, Weng and co-workers[12] on the high stability and reactivity of copper bifluoride complexes compared to their fluoride analogues, we became intrigued by the possibility of applying this methodology to NHC gold(I) complexes. Even though late transition metal bifluoride complexes have been known for over three decades,[11–15] the use of late transition metal bifluorides is still underdeveloped. Most reported complexes are phosphine or amine-based with only two examples bearing a NHC by Riant, Leyssens and co-workers[11] (Cu) and more recently by Whittlesey and co-workers (Rh).[15] We now report on the synthesis of new NHC gold(I) bifluoride complexes and their subsequent role in enabling the hydrofluorination of a range of alkynes.

Strategies consisting of late transition metal *tert*-butoxide complexes and triethylamine trihydrofluoride (NEt_3_**⋅**3 HF) proved unreliable when applied to NHC gold(I) complexes, due mainly to the poor stability of the [Au(NHC)(O*t*Bu)] complexes. Previously, we have reported on the synthesis of [Au(IPr)(OH)] (**1**).[16] We proposed that **1** could be a viable alternative to gold *tert*-butoxide complexes, thus permitting access to a wide variety of gold bifluoride complexes. Preliminary results proved encouraging, affording exclusively the cationic [[Au(IPr)(NEt_3_)](HF_2_)] complex (**2 a**, Scheme 1). Notably, during our initial optimisations, the gold fluoride complex **3** was always observed but its formation was suppressed subsequently by controlling the amount and dilution of NEt_3_**⋅**3 HF.

**Scheme 1:**
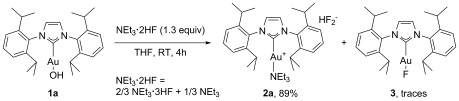
Initial synthetic route to complex 2 a.

The reaction was extended to various *N,N′*-bis(2,6-diisopropylphenyl)imidazol-2-ylidene (IPr)-based NHCs, whereby six new bifluoride complexes (**2 a**–**f**) were isolated in good yields and characterised fully (Scheme 2). Furthermore, the reaction was effectively scaled to 1 g for the IPr-bearing complex **2 a**.

**Scheme 2:**
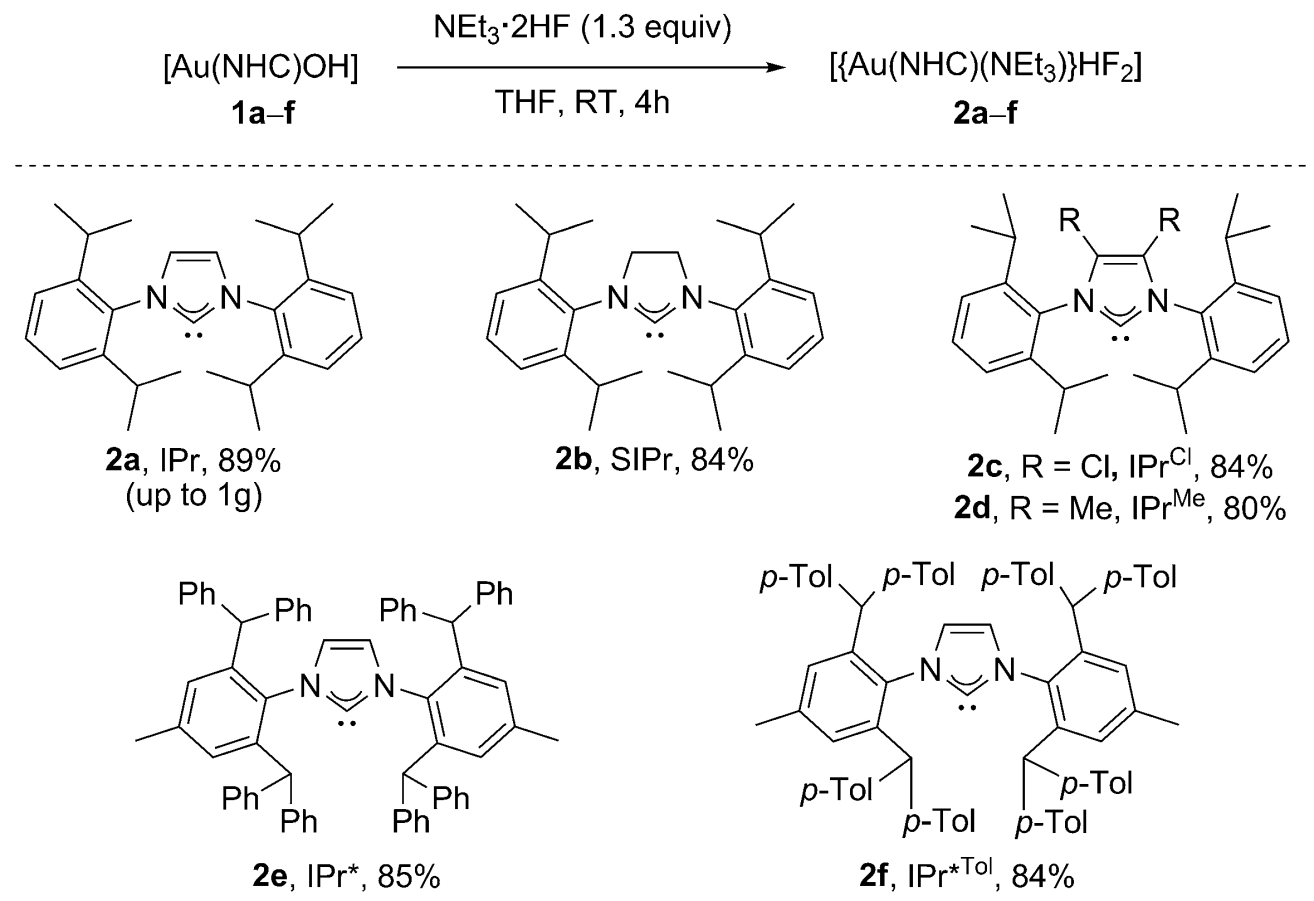
Synthesis of [[Au(NHC)(NEt_3_)](HF_2_)] complexes 2 a–f.

In an attempt to exchange the triethylamine moiety for other ligands, we found that the former could be substituted by pyridine by two methods (Scheme 3): reacting [Au(NHC)OH] either with diluted pyridine hydrofluoride (Py**⋅**HF; method A) or NEt_3_**⋅**3 HF in the presence of 1 equivalent of pyridine (method B) readily afforded the desired [[Au(NHC)(Py)](HF_2_)] complexes **4 a**,**b**. Method B was particularly interesting as it allowed for other ligands to be used. This strategy was exploited further by using Se(IPr) as ligand,[17] thus affording the desired cationic complex **5** in good yield. To the best of our knowledge, complex **5** constitutes the first example of a Se-based bifluoride.

**Scheme 3:**
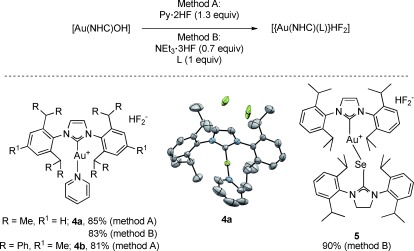
Synthesis of [[Au(NHC)(L)](HF_2_)] complexes and X-ray crystal structure of 4 a. All hydrogen atoms are omitted for clarity (see the Supporting Information for the disorder found for the HF_2_^−^ anion).

All gold bifluorides were air and moisture-stable in the solid state, and may be considered an anhydrous source of fluoride. More importantly, these species were also highly stable in solution if stored in plastic vials. The ^19^F NMR signal of these bifluorides showed a broad singlet at approximately −170 ppm, characteristic of a symmetric FHF^−^ anion.[11] The acidic proton of the HF_2_^−^ moiety for complexes **2 a**–**d** was observed in CD_2_Cl_2_, whereas the remaining bifluorides only revealed this proton in CD_3_CN.[18a] In all cases, the proton resonated between 13.2 and 13.7 ppm, which is consistent with the structure of these complexes.[11a] Single crystals of **4 a** and **5** were grown and X-ray analysis confirmed their structure (see the Supporting Information).[18b]

In an attempt to selectively form the gold monofluoride species, **1** and NEt_3_**⋅**3 HF (0.33 equiv) were reacted in benzene[10] and a mixture of mono- and bifluoride complexes were obtained in a 70:30 ratio. However, when the [Au(IPr)(O*t*Am)][16] (**6**; Am=amyl) complex was reacted under the same conditions, only the gold monofluoride was observed.[19] Therefore, we reasoned that these two species might be in equilibrium and the presence of H_2_O as a by-product (from the gold hydroxide reaction) was sufficient to shift the equilibrium towards the bifluoride. This phenomenon could be especially important for catalytic fluorination or fluorination and ring-opening processes, in which catalysts, particularly metal fluorides, are exposed to an excess of a HF source.[3a,5b,^7^,10a]

To examine the persistence of the bifluoride moiety in the presence of a base, [[Au(IPr)(NEt_3_)](HF_2_)] (**2 a**) was reacted with a stoichiometric amount of KO*t*Bu. Full conversion to the corresponding monofluoride (**3**) was observed within 4 h. Moreover, addition of diluted NEt_3_**⋅**3 HF (NEt_3_**⋅**2 HF) to **3** rapidly regenerated the metal bifluoride (**2 a**).[20] Hence, these two species were found to be inherently linked (Scheme 4) and in the presence of an excess of NEt_3_**⋅**3 HF or residual H_2_O, a metal fluoride or hydroxide would more than likely have delivered the metal bifluoride in situ. This might have implications for the future design and development of transition metal-catalysed fluorination methodologies.

**Scheme 4:**
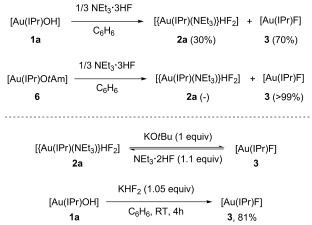
Gold fluoride versus gold bifluoride species.

We next reasoned that if KOR was able to deprotonate the gold bifluoride species, the reverse procedure using gold hydroxide species and KHF_2_ could be possible, thus affording the desired gold fluoride complexes. Gratifyingly, [Au(IPr)OH] complex (**1 a**) was able to deprotonate inexpensive and readily available KHF_2_ and deliver the desired [Au(IPr)F] (**3**) in 81 % yield (Scheme 4).

The newly prepared gold bifluoride complexes were then tested in the catalytic hydrofluorination of alkynes as convenient and air-stable alternatives to the previously described gold catalysts.[10,21] The initial optimisation reactions were performed by using diphenylacetylene (**7 a**) as a model substrate. After an extensive optimisation, complexes bearing bulkier ligands IPr* (**2 e**) and IPr*^Tol^ (**2 f**) proved most effective (see the Supporting Information). The use of a Py or Se moiety instead of NEt_3_ gave poorer results, which could be explained by their stronger coordination to the gold centre. The final optimal conditions comprised the use of complexes **2 e** or **2 f** in CH_2_Cl_2_ at 50 °C in a sealed plastic vial. The use of the NEt_3_**⋅**3 HF (3 equiv)/NH_4_BF_4_ (1.5 equiv) system allowed for lower catalyst loadings and shorter reaction times (see the Supporting Information, Tables 1–4).

The optimised conditions were then applied to various symmetrical alkynes (Scheme 5). Generally, excellent isolated yields were obtained for **8 a**–**i** with a wide range of substitution patterns. The reaction conditions tolerated bulky groups and electron-withdrawing and donating groups in *meta* and *para* positions. However, the use of *ortho*-substituted alkynes led to no reactivity, presumably for steric reasons. Notably, in each case only the *Z* isomer was afforded, with no *E* isomer observed. The use of unsymmetrical alkynes was then examined (Scheme 6). In addition to stereoselectivity issues, these substrates also introduced a regioselectivity challenge, thus four different products could be obtained. Early test reactions showed that only the *Z* isomers were obtained. However, elevated temperatures eroded the regioselectivity and traces of the minor *Z* isomer were observed by ^19^F NMR. Therefore, the reactions were performed at room temperature and the lower reactivity was offset by increasing the reaction time to 5 days. This procedure allowed expedient access to compounds that were otherwise extremely difficult to generate. Catalyst **6 f** was also employed, as preliminary reactions had shown it to lead to improved selectivities. The method differentiated quite selectively between alkyl and aryl groups (**10 a**–**b**) and only one isomer was obtained, even if the alkyl group was a benzyl moiety (**10 e**). More interestingly, substrates **9 c** and **9 d** allowed for the 1,4-addition of fluoride and the desired products were obtained with high selectivity. The formation of enone **10 c** was the only reaction without complete regioselectivity, but the selectivity was still high (especially for this class of substrates) and the major product could be isolated straightforwardly. Finally, **9 f** also afforded the desired product **10 f** in high yield and selectivity. To the best of our knowledge, this last example constitutes the first of this class of substrates to be catalytically hydrofluorinated in an efficient and selective manner.[22]

**Scheme 5:**
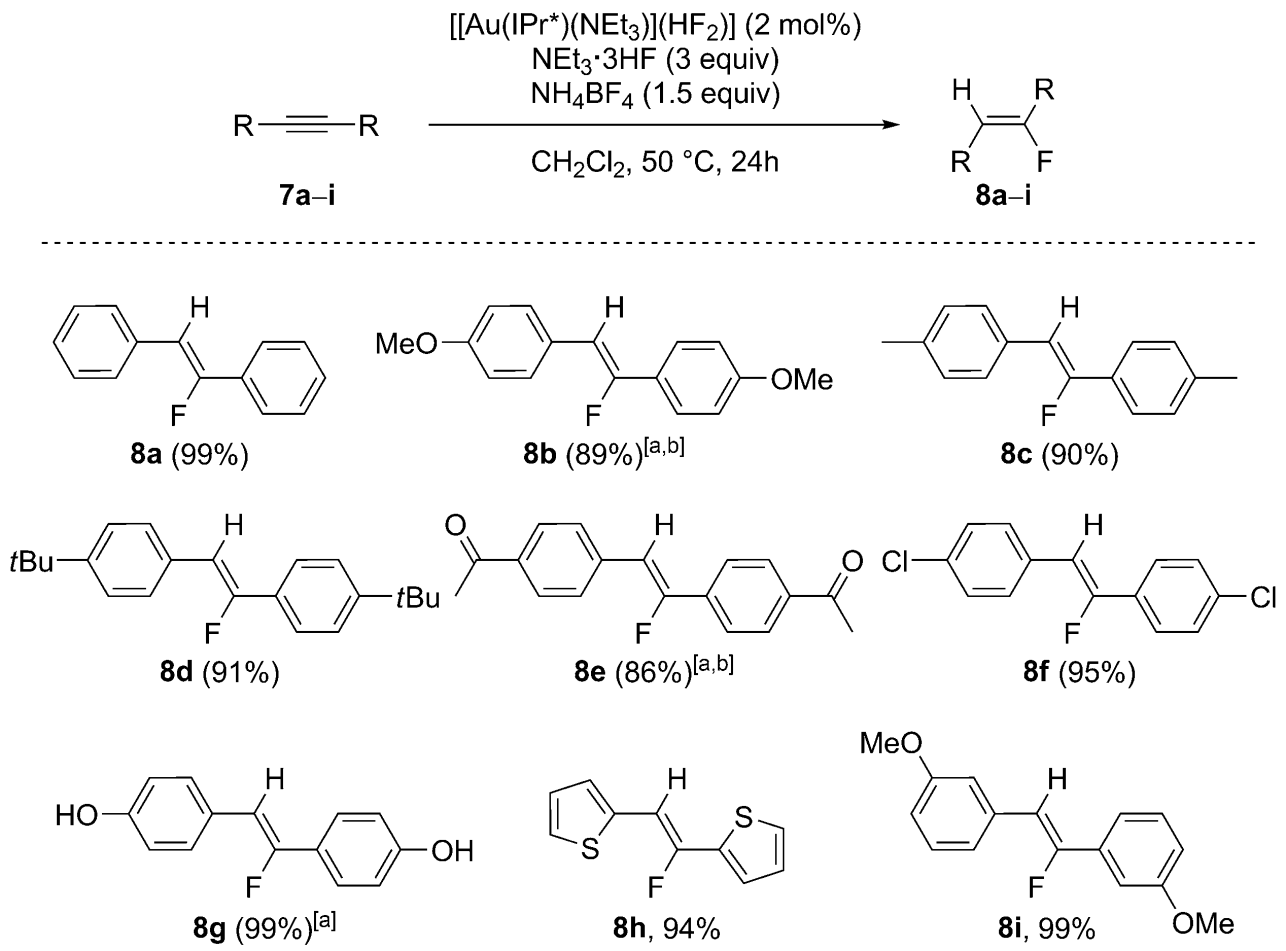
Hydrofluorination of symmetrical alkynes. Reaction conditions: alkynes 7 a–i (0.5 mmol), 2 e (2 mol %), NEt_3_⋅3 HF (1.5 mmol, 3 equiv), NH_4_BF_4_ (0.75 mmol, 1.5 equiv) in CH_2_Cl_2_ (0.7 m) at 50 °C. [a] *t*=48 h; [b] 4 mol % of 2 e used.

**Scheme 6:**
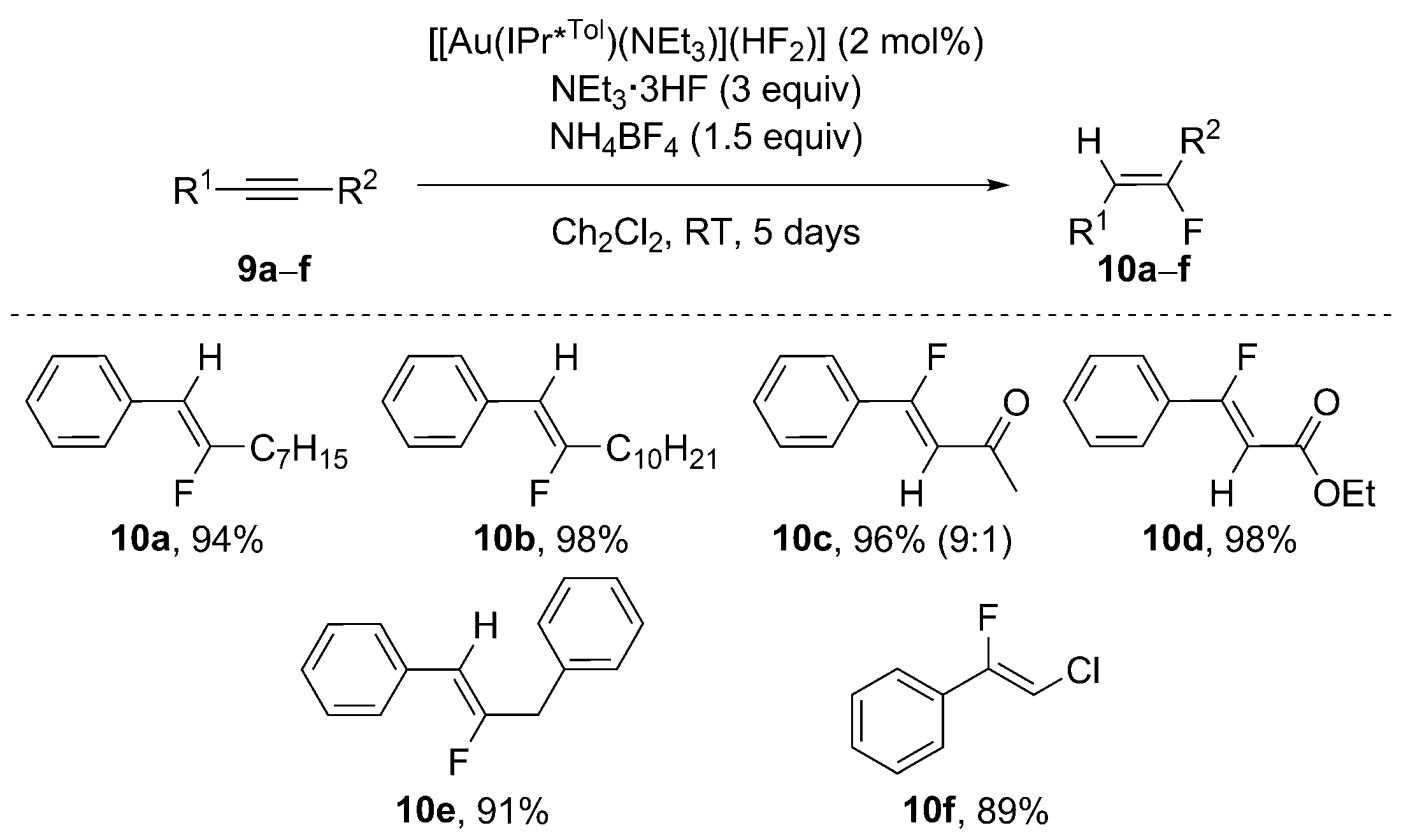
Hydrofluorination of unsymmetrical alkynes. Reaction conditions: alkynes 9 a–f (0.5 mmol), 2 f (2 mol %), NEt_3_⋅3 HF (1.5 mmol, 3 equiv), NH_4_BF_4_ (0.75 mmol, 1.5 equiv) in CH_2_Cl_2_ (0.7 m) at RT.

To fully exploit this strategy, we applied these reaction conditions to a highly functionalised unsymmetrical alkyne. Alkyne **9 g** was synthesised by using a modified literature procedure[23] and employed to prepare a fluorinated combretastatin analogue (Scheme 7).[24] Gratifyingly, 40 % conversion to the desired product **10 g** was observed with high regioselectivity and stereoselectivity (Scheme 7). Only the major regioisomer was isolated in 32 % yield,[25] characterised fully and its correct structure determined (shown in Scheme 7). This non-optimised procedure is the subject of an on-going investigation in our group.

**Scheme 7:**
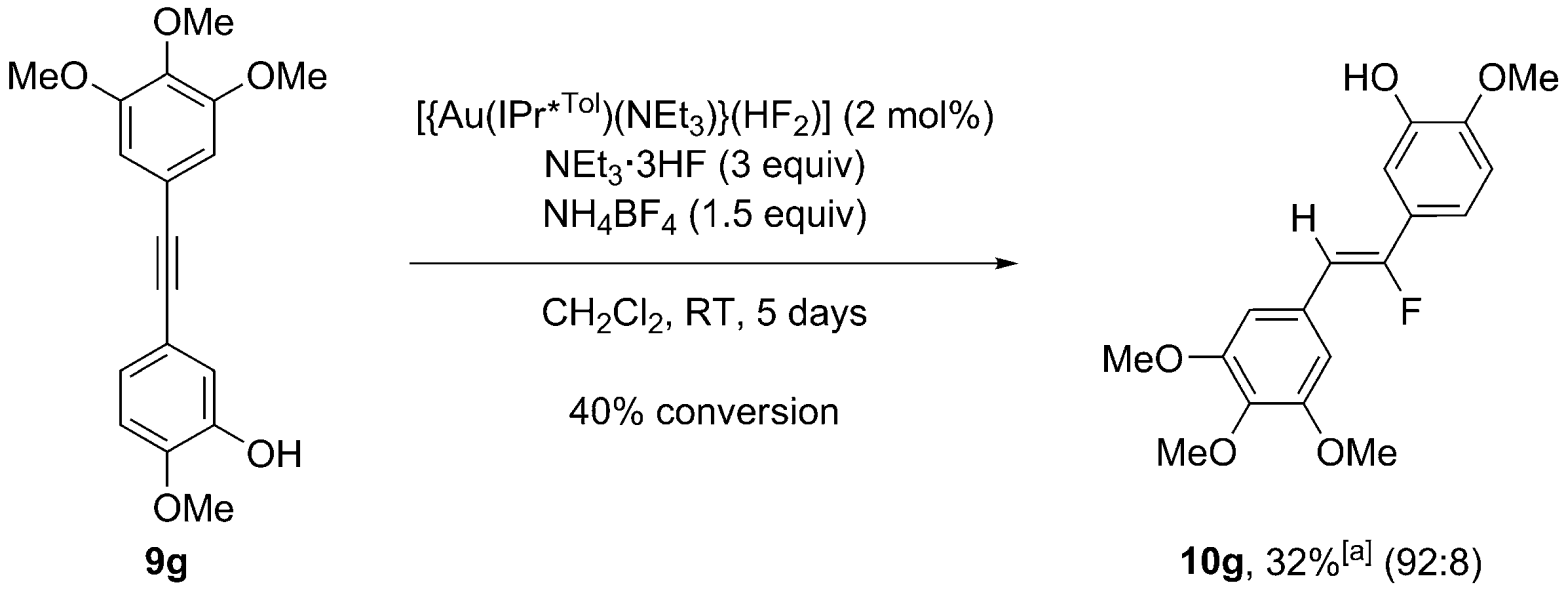
Synthesis and hydrofluorination of alkyne 9 g. Reaction conditions: alkyne 9 g (0.5 mmol), 2 f (2 mol %), NEt_3_⋅3 HF (1.5 mmol, 3 equiv), NH_4_BF_4_ (0.75 mmol, 1.5 equiv) in CH_2_Cl_2_ (0.7 m) at RT. [a] Corrected isolated yield.[25]

We were also interested in the hydrofluorination of alkynyl sulfides **11** to afford fluorovinyl thioethers **12**. The latter are considered putative biomimetic surrogates for the enol(ate) of thioesters.[26] Attempts to hydrofluorinate these substrates by using a range of literature procedures[10a,21a–b] proved unsuccessful and our alternative protocol was subsequently tested on this substrate class (Scheme 8). Reactions were performed at room temperature until complete consumption of the starting material was observed.[27] Gratifyingly, the desired products **12 a**–**c** were obtained in good yields and excellent stereoselectivities. Interestingly, the stereoselectivity proved independent of reaction conditions; the fluorine addition occurred α or β to the sulfur if terminal (**11 a**–**b**) or internal (**11 c**) sulfides were used, respectively.

**Scheme 8:**
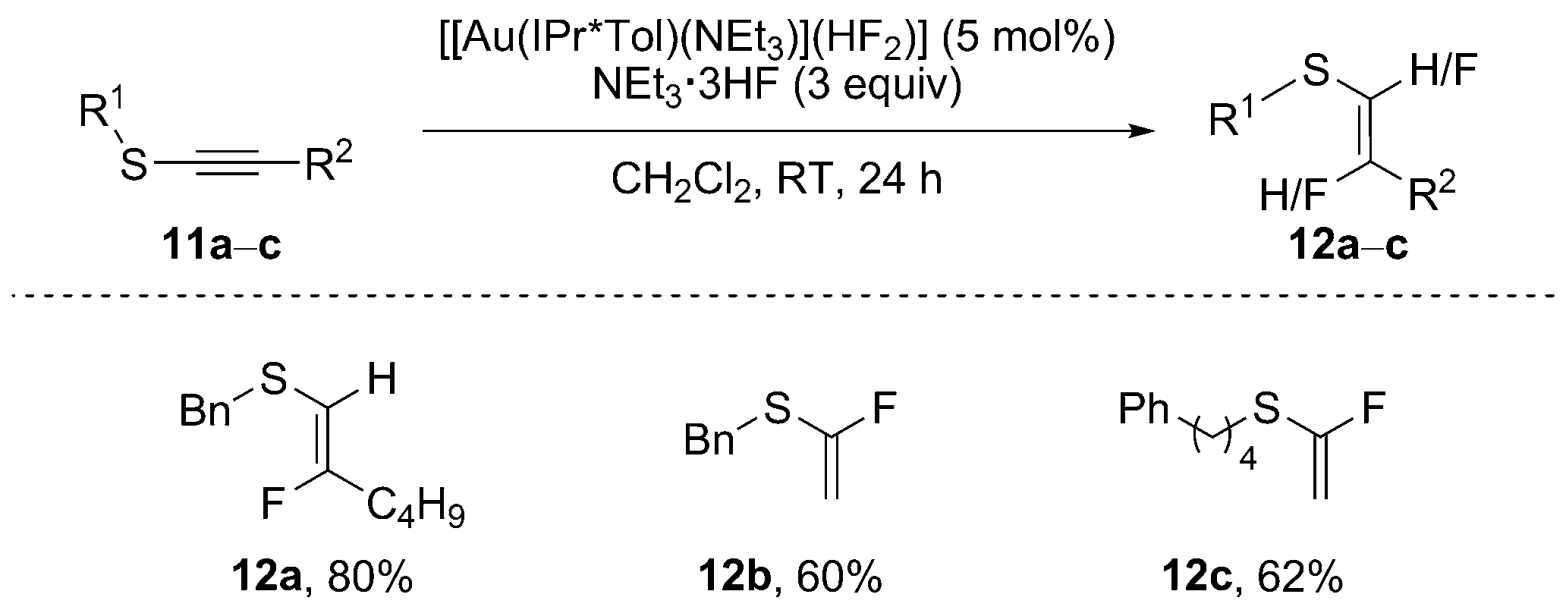
Access to fluorovinyl thioethers through hydrofluorination. Reaction conditions: 12 a–c (0.5 mmol), 2 f (2 mol %), NEt_3_⋅3 HF (1.5 mmol, 3 equiv) in CH_2_Cl_2_ (0.7 m) at RT.

In conclusion, nine novel *N*-heterocyclic carbene gold bifluoride complexes have been prepared from the corresponding gold(I) hydroxides. The methodology is high yielding, reproducible and scalable. Most importantly, these complexes are highly stable to air and moisture and are thus proposed as practical surrogates for the sensitive gold(I) fluorides. A new methodology to access gold(I) fluorides by using inexpensive and benign potassium bifluoride has been developed. The gold(I) fluorides and bifluorides are shown to be in equilibrium and, as a function of reaction conditions, these two species can readily interconvert. The gold(I) bifluorides have proven to be efficient catalysts in the hydrofluorination of symmetrical and unsymmetrical alkynes, thus affording fluorinated stilbene analogues and fluorovinyl thioethers in good to excellent yields with high stereo- and regioselectivity. Highly substituted and functionalised alkynes have been hydrofluorinated. The method is exploited further to selectively access a fluorinated combretastatin analogue in two steps starting from commercially available reagents, thus further affirming the usefulness of the protocol.
