# Supplementary material for: Hydrofluorination of Alkynes Catalysed by Gold Bifluorides
Source: ChemCatChem. 2014 Nov 26;7(2):240–4. doi: 10.1002/cctc.201402891 (PMC4515107; doi:10.1002/cctc.201402891)
Supplement: Supplementary file 1 [file cctc0007-0240-sd1.pdf]

Heterogeneous & Homogeneous & Bio-
CHEMCATCHEM
CATALYSIS

Supporting Information

© Copyright Wiley-VCH Verlag GmbH & Co. KGaA, 69451 Weinheim, 2015

Hydrofluorination of Alkynes Catalysed by Gold Bifluorides

Fady Nahra, Scott R. Patrick, Davide Bello, Marcel Brill, Alan Obled, David B. Cordes, Alexandra M. Z. Slawin, David O'Hagan, and Steven P. Nolan*[a]

cctc_201402891_sm_miscellaneous_information.pdf

General Information **2**

Synthetic Procedures: Bifluoride complexes **2**

Optimisation Reactions: Hydrofluorination of diphenylacetylene (7a) **7**

Synthetic Procedures: Hydrofluorination reaction **9**

References **21**

NMR Spectra **23**

General Information

All reagents were used as received. Reagent grade tetrahydrofuran was distilled and filtered through basic alumina before use. Solvents used for work-up were of technical grade. Deuterated solvents were dried and degassed. CD_2Cl_2 was filtered through basic alumina to remove traces of HCl. Fluorine-containing gold complexes were stored in plastic bottles to avoid reactions with glass. ^{1}H and ^{13}C spectra were recorded on a Bruker Avance 300, Bruker Avance II 400 Ultrashield or Bruker Avance III 500 spectrometer. ^{1}H and ^{13}C NMR chemical shifts are reported relative to $CDCl_3$ (7.26, 77.16 ppm), CD_2Cl_2 (5.32, 53.84 ppm), C_6D_6 (7.16, 128.06) and CD_3CN (1.94, 118.26). Elemental analyses were performed by the London Metropolitan University Elemental Analysis Service. [Au(NHC)OH] (**1a-f**) and [Au(IPr)O^{t}Am] (**6**) complexes were synthesised according to known literature procedures.[1] Se(SIPr) was synthesised according to literature procedure.[2]

Synthetic Procedures: Bifluoride complexes

General procedure for the synthesis of [[Au(NHC)(NEt_3)](HF_2)]

An oven-dried round-bottomed flask was charged with [Au(NHC)OH] (1 equiv.), THF (0.08 M) and triethylamine (0.43 equiv.). The flask was closed with a septum, placed under an atmosphere of nitrogen and stirred vigorously at room temperature. Triethylamine trihydrofluoride (0.87 equiv.) was then added dropwise. The reaction mixture was stirred at room temperature for 4 h and then concentrated at low temperature, until the product started to precipitate. At that point, hexane (10-15 mL per 0.1 mmol) was added to form a white precipitate. The solid was collected by filtration on a Büchner funnel, washed with hexane and then dried *in vacuo*.

[[Au(IPr)(NEt_3)](HF_2)] (2a)

Prepared according to the general procedure to afford the desired product as a white microcrystalline solid (1.0732 g, 89%). ^{1}H NMR (500 MHz, CD_2Cl_2): δ 13.66 (br. s, 1H), 7.57 (t, J = 7.9 Hz, 2H), 7.45 (s, 2H), 7.36 (d, J = 7.9 Hz, 4H), 2.67 (q, J = 7.1 Hz, 6H), 2.49 (hept, J = 6.7 Hz, 4H), 1.29 (d, J = 6.7 Hz, 12H), 1.26 (d, J = 6.7 Hz, 12H), 0.80 (t, J = 7.3 Hz, 9H) ppm. ^{13}C{^{1}H}-DEPTQ NMR (125 MHz, CD_2Cl_2): δ 169.9 ($C_{carbene}$), 146.1 (C_{Ar}), 133.8 (C_{Ar}), 131.6 (C_{Ar}), 124.8 (C_{Ar} + CH_{imid}), 52.2 (CH_2CH_3), 29.2 (CH), 24.6 (CH_3), 24.3

(CH_3), 11.1 (CH_2CH_3) ppm. ^{19}F NMR (470 MHz, CD_2Cl_2): δ -168.9 (br. s) ppm. Anal. Calcd. for $C_{33}H_{52}AuF_2N_3$: C, 54.61; H, 7.22; N, 5.79. Found: C, 54.47; H, 7.34; N, 5.64.

[[Au(SIPr)(NEt$_3$)][(HF$_2$)] (2b)

Prepared according to the general procedure to afford the desired product as a white microcrystalline solid (756 mg, 84%). ^1H NMR (500 MHz, CD_2Cl_2): δ 13.68 (br. s, 1H), 7.47 (t, *J* = 7.8 Hz, 2H), 7.30 (d, *J* = 7.8 Hz, 4H), 4.24 (s, 4H), 3.04 (hept, *J* = 6.8 Hz, 4H), 2.60 (q, *J* = 6.8 Hz, 6H), 1.37 (t, *J* = 6.8 Hz, 24 H), 0.68 (t, *J* = 6.8 Hz, 9H) ppm. $^{13}C\{^1H\}$-DEPTQ NMR (125 MHz, CD_2Cl_2): δ 191.8 ($C_{carbene}$), 147.2 (C_{Ar}), 133.9 (C_{Ar}), 130.8 (CH_{Ar}), 125.1 (CH_{Ar}), 54.1 (CH_{2imid}), 51.4 (CH_2CH_3), 29.3 (CH), 25.2 (CH_3), 24.4 (CH_3), 10.4 (CH_2CH_3) ppm. ^{19}F NMR (470 MHz, CD_2Cl_2): δ -168.9 (br. s) ppm. Anal. Calcd. for $C_{33}H_{54}AuF_2N_3$: C, 54.46; H, 7.48; N, 5.77. Found: C, 54.25; H, 7.54; N, 5.73.

[[Au(IPr^{Cl})(NEt$_3$)](HF$_2$)] (2c)

Prepared according to the general procedure to afford the desired product as a white microcrystalline solid (500.0 mg, 84%). ^1H NMR (500 MHz, CD_3CN): δ 13.50 (br. s, 1H), 7.68 – 7.59 (m, 2H), 7.50 – 7.42 (m, 4H), 2.65 (q, *J* = 7.2 Hz, 6H), 2.55 – 2.45 (m, 4H), 1.28 (d, *J* = 6.9 Hz, 12H), 1.26 (d, *J* = 6.8 Hz, 12H), 0.71 (t, *J* = 7.2 Hz, 9H). $^{13}C\{^1H\}$ NMR (125 MHz, CD_2Cl_2): δ 169.6 ($C_{carbene}$), 146.5 (C_{Ar}), 132.6 (C_{Ar}), 131.0 (CH_{Ar}), 125.3 (CH_{Ar}), 120.8 (CCl_{imid}), 52.2 (CH_2CH_3), 29.6 (CH), 24.8 (CH_3), 23.7(CH_3), 8.7 (CH_2CH_3) ppm. ^{19}F NMR (470 MHz, CD_2Cl_2): δ -169.1 (br. s) ppm. Anal. Calcd. for $C_{33}H_{50}AuCl_2F_2N_3$: C, 49.88; H, 6.34; N, 5.29. Found: C, 49.72; H, 6.49; N, 5.24.

[[Au(IPr^{Me})(NEt$_3$)](HF$_2$)] (2d)

Prepared according to the general procedure to afford the desired product as a white microcrystalline solid (260.1 mg, 80%). ^1H NMR (500 MHz, CD_2Cl_2): δ 13.68 (br. s, 1H), 7.57 (t, *J* = 7.9 Hz, 2H), 7.37 (d, *J* = 7.9 Hz, 4H), 2.65 (q, *J* = 7.2 Hz, 6H), 2.40 (hept, *J* = 7.0 Hz, 4H), 2.06 (s, 6H), 1.28 (m, 24H), 0.76 (t, *J* = 7.0 Hz, 9H) ppm. $^{13}C\{^1H\}$ NMR (125 MHz, CD_2Cl_2): δ 165.6 ($C_{carbene}$), 146.3 (C_{Ar}), 132.3 (C_{Ar}), 131.4 (CH_{Ar}), 128.1 (C_{imid}), 124.9 (CH_{Ar}), 51.8 (CH_2CH_3), 29.0 (CH), 25.2 (CH_3), 23.6 (CH_3), 10.7 (CH_2CH_3), 9.8 (CH_3) ppm. ^{19}F NMR (470 MHz, CD_2Cl_2): δ -170.3 (br. s) ppm. Anal. Calcd. for $C_{35}H_{56}AuF_2N_3$: C, 55.77; H, 7.49; N, 5.57. Found: C, 55.57; H, 7.36; N, 5.71.

[[Au(IPr*)(NEt$_3$)](HF$_2$)] (2e)

Prepared according to the general procedure to afford the desired product as a white microcrystalline solid (471.9 mg, 85%). ^{1}H NMR (MHz, CD_2Cl_2): δ 7.32-7.20 (m, 24H), 7.08-7.03 (m, 12H), 6.90-6.85 (m, 8H), 5.96 (s, 2H), 5.21 (s, 4H), 2.64 (q, J = 7.1 Hz, 6H), 2.31 (s, 6H), 0.76 (t, J = 7.1 Hz, 9H) ppm. ^{1}H NMR (500 MHz, CD_3CN): δ 13.48 (br.s, 1H) ppm. ^{13}C{^{1}H}-DEPTQ NMR (125 MHz, CD_2Cl_2): δ 169.14 ($C_{carbene}$), 143.12 (C_{Ar}), 142.91 (C_{Ar}), 142.36 (C_{Ar}), 141.43 (C_{Ar}), 141.25 (C_{Ar}), 141.07 (C_{Ar}), 133.23 (C_{Ar}), 130.74 (CH_{Ar}), 130.58 (CH_{Ar}), 130.07 (CH_{Ar}), 129.77 (CH_{Ar}), 129.62 (CH_{Ar}), 129.28 (CH_{Ar}), 129.09 (CH_{Ar}), 129.08 (CH_{Ar}), 128.84 (CH_{Ar}), 128.80 (CH_{Ar}), 127.61 (CH_{Ar}), 127.59 (CH_{Ar}), 127.12 (CH_{Ar}), 127.06 (CH_{Ar}), 125.08 (C_{imid}), 52.05 (CH), 51.12 (CH$_2$CH$_3$), 21.92 (CH$_3$), 10.63 (CH$_2$$CH_3$) ppm. ^{19}F NMR (470 MHz, CD_3CN): δ -165.7 (br. s) ppm. Anal. Calcd. for $C_{75}H_{72}AuF_2N_3$: C, 72.04; H, 5.80; N, 3.36. Found: C, 71.86; H, 5.67; N, 3.27.

[[Au(IPr*^{Tol})(NEt$_3$)](HF$_2$)] (2f)

Prepared according to the general procedure to afford the desired product as a white microcrystalline solid (462.4 mg, 84%). ^{1}H NMR (500 MHz, CD_2Cl_2): δ 7.04-6.99 (m, 20H), 6.90 (d, J = 8.0, 8H), 6.73 (d, J = 8.0, 8H), 6.01 (s, 2H), 5.09 (s, 4H), 2.64 (q, J = 7.7 Hz, 6H), 2.33 (s, 12H), 2.30 (s, 18H), 0.76 (t, J = 7.7 Hz, 9H) ppm. ^{1}H NMR (500 MHz, CD_3CN): δ 13.57 (br. s, 1H) ppm.^{13}C{^{1}H} NMR (125 MHz, CD_2Cl_2): δ 168.9 ($C_{carbene}$), 141.4 (C_{Ar}), 141.0 (C_{Ar}), 140.1 (C_{Ar}), 139.6 (C_{Ar}), 137.3 (C_{Ar}), 137.1 (C_{Ar}), 133.2 (C_{Ar}), 130.4 (CH_{Ar}), 129.6 (CH_{Ar}), 129.6 (CH_{Ar}), 129.5 (CH_{Ar}), 129.1 (CH_{Ar}), 125.1 (CH_{imid}), 51.2 (CH$_2$CH$_3$), 51.0 (CH), 21.9 (CH$_3$), 21.1 (CH$_3$), 21.1 (CH$_3$), 10.7 (CH$_2$$CH_3$) ppm. ^{19}F NMR (470 MHz, CD_3CN): δ -166.2 (br. s) ppm. Anal. Calcd. for $C_{83}H_{88}AuF_2N_3$: C, 73.16; H, 6.51; N, 3.08. Found: C, 72.83; H, 6.64; N, 3.17.

General procedure for the synthesis of [[Au(NHC)(L)](HF$_2$)]

<u>Method A:</u>

An oven-dried round-bottomed flask was charged with [Au(NHC)OH] (1 equiv.), THF (0.08 M) and pyridine (0.3 equiv.). The flask was closed with a septum, placed under an atmosphere of nitrogen and stirred vigorously at room temperature. Pyridine hydrofluoride (1 equiv.) was then added dropwise. The reaction mixture was stirred at room temperature for 4 h and then concentrated at low temperature, until the product started to precipitate. At that

point, hexane (10-15 mL per 0.1 mmol) was added to form a white precipitate. The solid was collected by filtration on a Büchner funnel, washed with hexane and then dried *in vacuo*.

Method B:

An oven-dried round-bottomed flask was charged with [Au(NHC)OH] (1 equiv.), THF (0.08 M) and the indicated ligand (1 equiv.). The flask was closed with a septum, placed under an atmosphere of nitrogen and stirred vigorously at room temperature. Triethylamine trihydrofluoride (0.7 equiv.) was then added dropwise. The reaction mixture was stirred at room temperature for 4 h and then concentrated at low temperature, until the product started to precipitate. At that point, hexane (10-15 mL per 0.1 mmol) was added to form a white precipitate. The solid was collected by filtration on a Büchner funnel, washed with hexane and then dried *in vacuo*.

[[Au(IPr)(pyridine)](HF_2)] (4a)

Prepared according to the general procedure to afford the desired product as a white microcrystalline solid. Method A: (99 mg, 85%). Method B: (97 mg, 83%). ^{1}H NMR (500 MHz, CD_2Cl_2): δ 8.12-7.81 (m, 2H), 7.63-7.50 (m, 4H), 7.45 (s, 2H), 7.39 (d, *J* = 7.8 Hz, 4H), 2.57 (hept, *J* = 6.8 Hz, 4H), 1.35 (d, *J* = 6.8 Hz, 12H), 1.29 (d, *J* = 6.8 Hz, 12H) ppm. ^{1}H NMR (500 MHz, CD_3CN): δ 13.18 (br. s, 1H) ppm. ^{13}C{^{1}H} NMR (125 MHz, CD_2Cl_2): δ 168.0 ($C_{carbene}$), 150.8 (CH_{Py}), 146.1 (C_{Ar}), 142.9 (CH_{Py}), 133.7 (C_{Ar}), 131.5 (CH_{imid}), 127.8 (CH_{Py}), 125.1 (CH_{Ar}), 124.9 (CH_{Ar}), 29.2 ($CH(CH_3)_2$), 24.9 ($CH(CH_3)_2$), 24.1 ($CH(CH_3)_2$) ppm. ^{19}F NMR (470 MHz, CD_2Cl_2): δ -170.0 (br. s) ppm. Anal. Calcd. for $C_{32}H_{42}AuF_2N_3$: C, 54.62; H, 6.02; N, 5.97. Found: C, 54.55; H, 6.12; N, 5.90.

Crystals suitable for X-ray diffraction analysis were grown by layering a concentrated THF solution at -20 ^{o}C with hexane overnight.

[[Au(IPr*)(pyridine)](HF_2)] (4b)

Prepared according to the general procedure to afford the desired product as a white microcrystalline solid. Method A: (164.8 mg, 81%). ^{1}H NMR (500 MHz, CD_2Cl_2): δ 8.18 (t, *J* = 7.6 Hz, 1H), 7.67 (t, *J* = 7.6 Hz, 2H), 7.62-7.57 (m, 2H), 7.32-7.26 (m, 12H), 7.18 (t, *J* = 7.4 Hz, 4H), 7.07 (t, *J* = 7.4 Hz, 8H), 6.99 (s, 4H), 6.98-6.91 (m, 16H), 6.31 (s, 2H), 5.23 (s, 4H), 2.28 (s, 6H) ppm. ^{13}C{^{1}H} NMR (125 MHz, CD_2Cl_2): δ 167.8 ($C_{carbene}$), 150.88 (CH_{Py}), 143.2(CH_{Py}), 142.9 (C_{Ar}), 142.5 (C_{Ar}), 141.3 (C_{Ar}), 140.9 (NC_{Ar}), 133.3 (CH_{Ar}), 131.0

(CH_{Ar}), 129.8 (CH_{Ar}), 129.6 (CH_{Ar}), 129.1 (CH_{Ar}), 129.0 (CH_{Ar}), 128.8 (CH_{Py}), 127.6 (CH_{Ar}), 127.4 (CH_{Ar}), 125.3 (CH_{imid}), 51.8 (CH), 21.9 (CH_3) ppm. ^{19}F NMR (470 MHz, CD_2Cl_2): δ -153.2 (br. s) ppm. Anal. Calcd. for $C_{74}H_{62}AuF_2N_3$: C, 72.36; H, 5.09; N, 3.42. Found: C, 72.19; H, 5.22; N, 3.42.

[[Au(IPr)(Se(SIPr))](HF$_2$)] (5)

Prepared according to the general procedure to afford the desired product as a white microcrystalline solid. Method B: (163.5 mg, 90%). ^{1}H NMR (500 MHz, CD_2Cl_2): δ 7.52 – 7.44 (m, 2H), 7.33 – 7.22 (m, 6H), 7.18 (s, 2H), 7.17 – 7.10 (m, 4H), 4.00 (s, 4H), 2.67 (dt, J = 13.7, 6.9 Hz, 4H), 2.43 (dt, J = 13.7, 6.9 Hz, 4H), 1.20 (d, J = 6.9 Hz, 12H), 1.18 (d, J = 6.9 Hz, 12H), 1.09 (d, J = 6.9 Hz, 12H), 0.94 (d, J = 6.8 Hz, 12H) ppm. ^{1}H NMR (500 MHz, CD_3CN): δ 13.49 (br. s, 1H) ppm. ^{13}C{^{1}H} NMR (101 MHz, CD_2Cl_2): δ 181.7 (Se-$C_{carbene}$), 175.6(Au-$C_{carbene}$), 147.4, 146.43, 135.1, 133.6, 131.6, 131.6, 131.4, 126.27, 125.7, 125.2, 125.0, 53.7, 29.6, 29.5, 24.8, 24.7, 24.5, 23.9 ppm. ^{19}F NMR (470 MHz, CD_2Cl_2): δ -170.1 (br. s) ppm. ^{77}Se NMR (95 MHz, CD_3CN): δ 117 ppm. Anal. Calcd. for $C_{54}H_{75}AuF_2N_4Se$: C, 59.28; H, 6.91; N, 5.12. Found: C, 59.05; H, 7.06; N, 5.16.

Crystals suitable for X-ray diffraction analysis were grown by a slow vapour diffusion of hexane into a concentrated solution of **4a** in THF at room temperature.

General procedure for the synthesis of [Au(IPr)F]

A vial was charged with [Au(IPr)OH] (1 equiv.) and KHF_2 (1.05 equiv.) in benzene (0.3 M) and the reaction mixture was stirred at room temperature for 4 h. Once complete, the solvent was removed *in vacuo*. The residue was dissolved in THF and filtered through celite (the celite was washed with THF). The filtrate was concentrated *in vacuo* until the product started to precipitate. At that point, hexane (10-15 mL per 0.1 mmol) was added to form a white precipitate. The solid was collected by filtration on a Büchner funnel, washed with hexane and then dried *in vacuo*. The desired product was obtained as a white microcrystalline solid (325.3 mg, 81%).

[Au(IPr)F] (3)

^{1}H NMR (400 MHz, CD_2Cl_2): δ 7.57 – 7.52 (m, 2H), 7.36 – 7.32 (m, 4H), 7.17 (s, 2H), 2.65 – 2.50 (m, 4H), 1.35 (d, J = 6.9 Hz, 12H), 1.22 (d, J = 6.9 Hz, 12H) ppm. ^{13}C{^{1}H} NMR (101 MHz, CD_2Cl_2): δ 172.7, 146.3, 134.9, 130.8, 124.6, 123.4, 29.2, 24.5, 24.2 ppm. ^{19}F NMR

(377 MHz, CD_2Cl_2): δ -249.8 (s) ppm. Anal. Calcd. for $C_{27}H_{36}AuFN_2$: C, 53.64; H, 6.00; N, 4.63. Found: C, 53.67; H, 6.06; N, 4.82.

Optimisation Reactions: Hydrofluorination of diphenylacetylene (7a)

Table 1. Catalyst screening.

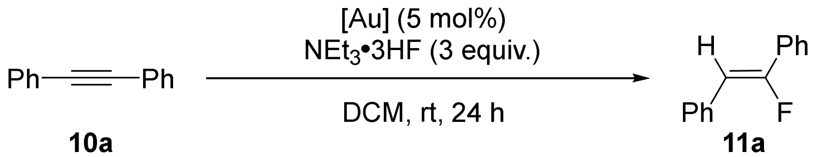

10a **11a**

Entry	[Au]	Conversion (%) [a]
1	-	0
2	[Au(IPr)(NEt_3)][HF_2] (**2a**)	22
3	[Au(SIPr)(NEt_3)][HF_2] (**2b**)	17
4	[Au(IPr^{Cl})(NEt_3)][HF_2] (**2c**)	35
5	[Au(IPr^{Me})(NEt_3)][HF_2] (**2d**)	11
**6**	**[Au(IPr*)(NEt_3)][HF_2] (2e)**	**40**
**7**	**[Au(IPr^{*Tol})(NEt_3)][HF_2] (2f)**	**52**
8	[Au(IPr)(Py)][HF_2] (**4a**)	2
9	[Au(IPr)(Se(SIPr))][HF_2] (**5**)	0

[a] Conversions determined by ^{1}H NMR.

Catalyst **2e** and **2f** proved to be the best catalysts for this reaction. However, due to the better availability and easier synthesis of the IPr* ligand in our lab contrary to the IPr^{*Tol} counterpart, catalyst **2e** was chosen for further optimisation.

Table 2. Solvent effect on the hydrofluorination reaction.

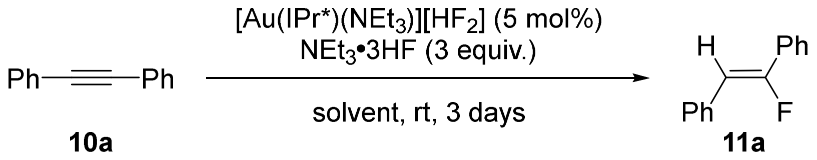

Entry	Solvent	Conversion (%) [a]
**1**	**DCM**	**61**
2	THF	8
**3**	**Toluene**	**35**
4	1,2-DCE	36
5	MeCN	15
6	^{t}AmOH	27

[a] Conversions determined by ^{1}H NMR.

DCM was clearly the best solvent. However toluene was also kept for the temperature screening performed later on (see table 4).

Table 3. Additive screening.

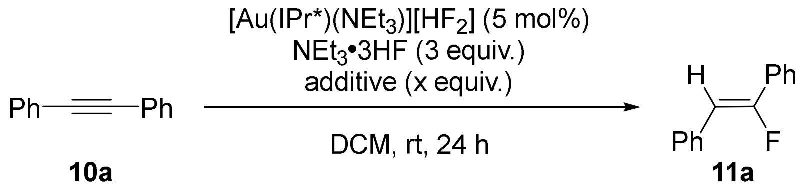

Entry	Additive (x equiv.)	Conversion (%) [a]
**1**	**$N^{t}Bu_4HSO_4$ (0.3)**	**49**
**2**	**NH_4BF_4 (0.3)**	**49**
3	$NaHCO_3$ (0.3)	34
4	Na_2CO_3 (0.3)	24
5	HOTf (1.0)	>99 [b]
6	HOAc (1.0)	>99 [b]
7	TFA (1.0)	12

[a] Conversions determined by ^{1}H NMR. [b] Disappearance of starting alkyne after 24 h; side-product formation observed.

Ammonium tetrafluoroborate was chosen for further optimisation.

Table 4. Final optimisation reactions.

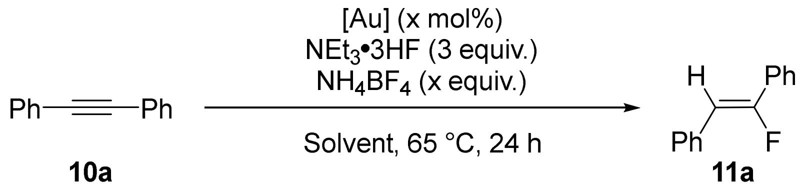

Entry	[Au] (x mol%)	Solvent	NH_4BF_4 (x equiv.)	Conversion (%) ^{a}
1	[Au(IPr*)(NEt_3)][HF_2] (5.0)	Toluene	0	69
2	[Au(IPr*)(NEt_3)][HF_2] (5.0)	Toluene	1.0	90
**3**	**[Au(IPr*)(NEt_3)][HF_2] (5.0)**	**Toluene**	**1.5**	**>99**
**4**	**[Au(IPr*)(NEt_3)][HF_2] (2.0)**	**Toluene**	**1.5**	**>99**
5	[Au(IPr*)(NEt_3)][HF_2] (1.0)	Toluene	1.5	95
6	[Au(IPr*)(NEt_3)][HF_2] (1.0)	Toluene	2.0	88
**7**	**[Au(IPr*^{Tol})(NEt_3)][HF_2] (2.0)**	**Toluene**	**1.5**	**>99**
8	[Au(IPr*^{Tol})(NEt_3)][HF_2] (1.0)	Toluene	1.5	94
**9**^{b}	**[Au(IPr*)(NEt_3)][HF_2] (2.0)**	**DCM**	**1.5**	**>99**
**10**^{b}	**[Au(IPr*^{Tol})(NEt_3)][HF_2] (2.0)**	**DCM**	**1.5**	**>99**

^{a} Conversions determined by ^{1}H NMR. ^{b} T = 50 °C.

Synthetic Procedures: Hydrofluorination reaction

Starting material syntheses

Symmetrical alkynes[3a] and non-symmetrical alkynes[4] were prepared according to known procedures. Analytical data obtained was in accordance with the reported values (**8a-e**,[3a] **8h**,[3a] **8f**,[3b] **8g**,[3c] **8i**[3d] and **10a-b**,[4a] **10e**[4b]).

Procedure for 8a-i:

A flame-dried round bottom flask was charged with $(PPh_3)_2PdCl_2$ (0.15 mmol, 0.05 equiv.), 1,4-Di(diphenylphosphino)butane (0.30 mmol, 0.1 equiv.) and DMSO (0.2 M) under argon. The reaction mixture was stirred while adding the corresponding aryl halide (6 mmol, 2 equiv.) and the 2-butynedioic acid (3 mmol, 1 equiv.), followed by the addition of 1,8-diazabicyclo(5.4.0)undec-7-ene (6 mmol, 2 equiv.). The reaction mixture was then stirred at 110 °C for 6 hours. Afterwards, the reaction mixture was directly poured into saturated aqueous ammonium chloride and extracted with Et_2O. The combined organic phases were washed with brine, dried over $MgSO_4$ and filtered. The solvent was removed under vacuum

and the residue was purified by flash column chromatography (silica gel, pentane or pentane/EtOAc mixture or EtOAc were used as eluent).

Procedure for 10a-b:

A flame-dried Schlenk was charged with phenylacetylene (6 mmol, 1.2 equiv.) and THF (0.2 M) under argon. The reaction mixture was cooled at -78 °C, followed by a dropwise addition of *n*-Butyllithium (1.6 M in hexanes, 5.5 mmol, 1.1 equiv.). The solution was allowed to warm to room temperature before adding the alkyl bromide (5 mmol, 1 equiv.) and tetrabutylammonium iodide (0.5 mmol, 0.1 equiv.). The reaction mixture was then stirred overnight at 80 °C. Afterwards, the mixture was cooled at 0 °C and then poured into a saturated aqueous ammonium chloride solution and extracted with Et_2O. The combined organic phases were washed with brine, dried over $MgSO_4$ and filtered. The solvent was removed under vacuum and the residue was purified by flash column chromatography (silica gel, pentane was used as eluent).

Procedure for 10e:

A flame-dried Schlenk was charged with $Pd(OAc)_2$ (0.05 mmol, 0.01 equiv.), 2-Dicyclohexylphosphino-2′,4′,6′-triisopropylbiphenyl (**XPHOS**) (0.1 mmol, 0.02 equiv.) and THF (0.25 M) under argon. The reaction mixture was then stirred while adding the phenylpropiolic acid (6 mmol, 1.2 equiv.), the corresponding halide (5 mmol, 1 equiv.) and followed by the addition of caesium carbonate (6 mmol, 1.2 equiv.). The reaction mixture was stirred overnight at 80 °C. Afterwards, the mixture was directly poured into water and extracted with Et_2O. The combined organic phases were washed with brine, dried over $MgSO_4$ and filtered. The solvent was removed under vacuum and the residue was purified by flash column chromatography (silica gel, pentane was used as eluent).

Synthesis of 2-methoxy-5-((3,4,5-trimethoxyphenyl)ethynyl)phenol (9g)

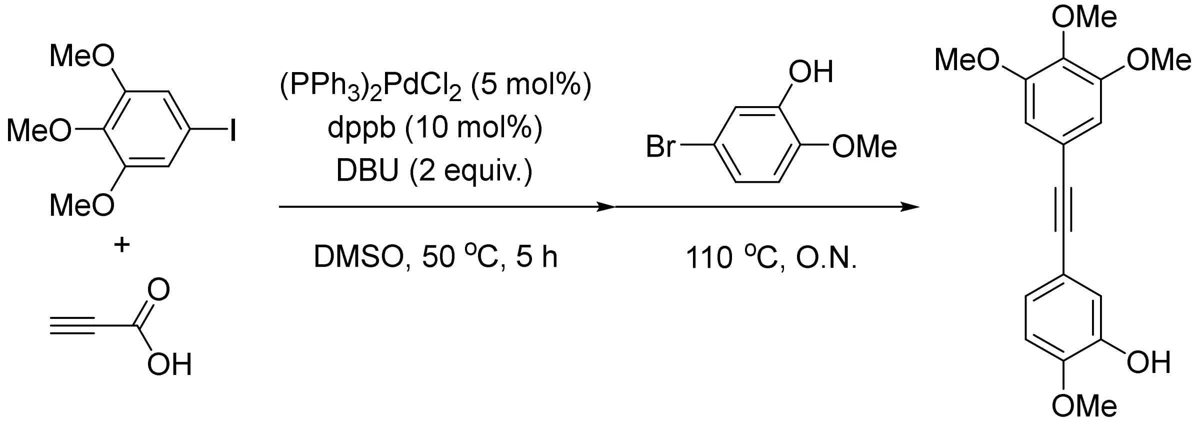

9g, 37%

A flame-dried round bottom flask was charged with $(PPh_3)_2PdCl_2$ (105 mg, 0.15 mmol, 0.05 equiv.), 1,4-Di(diphenylphosphino)butane (128 mg, 0.30 mmol, 0.1 equiv.) and DMSO (10 mL) under argon. The reaction mixture was stirred while adding 5-iodo-1,2,3-trimethoxybenzene (882 mg, 3.0 mmol, 1 equiv.) and the propiolic acid (0.19 mL, 3.0 mmol, 1 equiv.), followed by the addition of 1,8-diazabicyclo(5.4.0)undec-7-ene (0.9 mL 6 mmol, 2 equiv.). The reaction mixture was then stirred at 50 °C for 5 hours. Afterwards, 5-bromo-2-methoxyphenol (609 mg, 3.0 mmol, 1 equiv.) was added and the reaction was stirred overnight at 110 ^{o}C. The reaction mixture was directly poured into saturated aqueous ammonium chloride and extracted with EtOAc. The combined organic phases were washed with brine, dried over $MgSO_4$ and filtered. The solvent was removed under vacuum and the residue was purified by flash column chromatography (silica gel, 7:3 pentane/EtOAc mixture was used as eluent) to obtain the desired product as yellow solid (356 mg, 37%). ^{1}H NMR (400 MHz, $CDCl_3$) δ 7.09 (d, J = 1.9 Hz, 1H), 7.05 (dd, J = 8.3, 1.9 Hz, 1H), 6.80 (d, J = 8.3 Hz, 1H), 6.75 (s, 2H), 5.70 (s, 1H), 3.89 (s, 3H), 3.86 (s, 6H), 3.86 (s, 3H). ^{13}C NMR (101 MHz, $CDCl_3$) δ 153.15, 147.14, 145.46, 138.69, 124.31, 118.63, 117.60, 116.03, 110.61, 108.77, 88.54, 87.98, 77.16, 61.07, 56.23, 56.02.

These data are in good agreement with the literature values.[5]

General procedure for the hydrofluorination reactions of alkynes

A plastic vial was charged with the corresponding alkyne (0.5 mmol, 1 equiv.) under argon. The indicated gold bifluoride catalyst (0.01 mmol, 0.02 equiv.) and NH_4BF_4 (0.75 mmol, 1.5 equiv.) were then added. DCM (0.7M) was added, followed by a dropwise addition of triethylamine trihydrofluoride (1.5 mmol, 3 equiv.). The reaction mixture was then stirred at the indicated temperature (50 ^{o}C for symmetrical alkynes or rt for asymmetrical alkynes) and time (24 hours for symmetrical alkynes or 5 days for asymmetrical alkynes). After total

consumption of the starting material, the reaction mixture was directly purified by flash column chromatography (silica gel, pentane or pentane/EtOAc mixture were used as eluent).

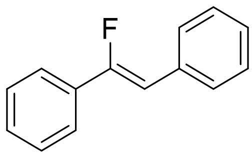

(Z)-(1-fluoroethene-1,2-diyl)dibenzene (8a)

Prepared according to the general procedure. Flash chromatography (silica gel, pentane) provided the desired product in 99% yield. ^{1}H NMR (400 MHz, $CDCl_3$) δ = 7.71 – 7.61 (m, 4H), 7.47 – 7.33 (m, 5H), 7.31 – 7.23 (m, 1H), 6.33 (d, *J* = 39.5 Hz, 1H). ^{13}C NMR (101 MHz, $CDCl_3$) δ = 157.4 (d, *J* = 258.5 Hz), 133.8 (d, *J* = 2.9 Hz), 133.0 (d, *J* = 27.9 Hz), 129.1, 129.1, 128.7, 127.5 (d, *J* = 2.3 Hz), 124.5 (d, *J* = 7.5 Hz), 106.0 (d, *J* = 10.4 Hz). ^{19}F NMR (377 MHz, $CDCl_3$) δ -114.2. HRMS m/z calcd. for $C_{14}H_{12}F$ $[M + H]^+$ 199.0918; found 199.0916.

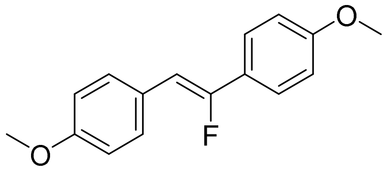

(Z)-4,4'-(1-fluoroethene-1,2-diyl)bis(methoxybenzene) (8b)

Prepared according to the general procedure. Flash chromatography (silica gel, pentane/EtOAc) provided the desired product in 89% yield. ^{1}H NMR (400 MHz, $CDCl_3$) δ 7.64 – 7.51 (m, 4H), 6.91 (dd, *J* = 8.7, 6.9 Hz, 4H), 6.13 (d, *J* = 40.1 Hz, 1H), 3.85 (s, 3H), 3.83 (s, 3H). ^{13}C NMR (101 MHz, $CDCl_3$) δ 160.2, 158.7 (d, *J* = 3.0 Hz), 156.2 (d, *J* = 255.1 Hz), 123.1 (d, *J* = 8.0 Hz), 126.9 (d, *J* = 2.8 Hz), 125.9 (d, *J* = 28.4 Hz), 125.7 (d, *J* = 7.4 Hz), 114.2, 103.8 (d, *J* = 11.1 Hz), 55.4, 55.4. ^{19}F NMR (377 MHz, $CDCl_3$) δ -116.2. HRMS m/z calcd. for $C_{16}H_{16}FO_2$ $[M + H]^+$ 259.1129; found 259.1128.

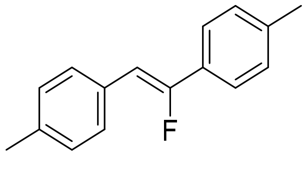

(Z)-4,4'-(1-fluoroethene-1,2-diyl)bis(methylbenzene) (8c)

Prepared according to the general procedure. Flash chromatography (silica gel, pentane) provided the desired product in 90% yield. ^{1}H NMR (400 MHz, $CDCl_3$) δ 7.55 – 7.50 (m,

4H), 7.23 – 7.15 (m, 4H), 6.23 (d, *J* = 39.9 Hz, 1H), 2.38 (s, 3H), 2.36 (s, 3H). ^{13}C NMR (101 MHz, $CDCl_3$) δ 157.1 (d, *J* = 257.0 Hz), 139.0, 137.1 (d, *J* = 2.3 Hz), 131.2 (d, *J* = 2.8 Hz), 130.4 (d, *J* = 28.0 Hz), 129.4, 128.9 (d, *J* = 7.9 Hz), 124.3 (d, *J* = 7.4 Hz), 105.1 (d, *J* = 10.7 Hz), 21.4, 21.4. ^{19}F NMR (377 MHz, $CDCl_3$) δ -114.9. HRMS m/z calcd. for $C_{16}H_{16}F$ [M + H]$^+$ 227.1231; found 227.1228.

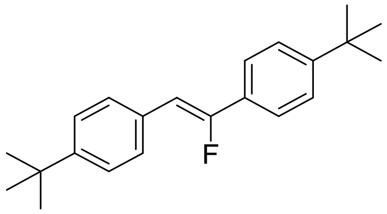

(Z)-4,4'-(1-fluoroethene-1,2-diyl)bis(tert-butylbenzene) (8d)

Prepared according to the general procedure. Flash chromatography (silica gel, pentane) provided the desired product in 91% yield. ^{1}H NMR (400 MHz, $CDCl_3$) δ 7.68 – 7.53 (m, 4H), 7.50 – 7.38 (m, 4H), 6.27 (d, *J* = 39.9 Hz, 1H), 1.35 (s, 9H), 1.35 (s, 9H). ^{13}C NMR (101 MHz, $CDCl_3$) δ 157.2 (d, *J* = 257.2 Hz), 152.3, 150.4, 131.2 (d, *J* = 2.8 Hz), 130.4 (d, *J* = 28.0 Hz), 128.8 (d, *J* = 7.8 Hz), 125.6, 124.2 (d, *J* = 7.3 Hz), 105.1 (d, *J* = 10.7 Hz), 34.9, 34.8, 31.4, 31.4. ^{19}F NMR (471 MHz, $CDCl_3$) δ -114.9. HRMS m/z calcd. for $C_{22}H_{28}F$ [M + H]$^+$ 311.2170; found 311.2172.

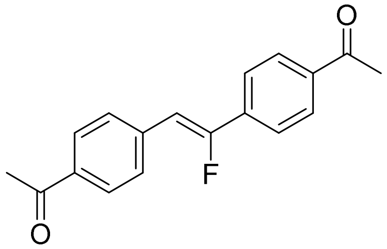

(Z)-1,1'-((1-fluoroethene-1,2-diyl)bis(4,1-phenylene))bis(ethan-1-one) (8e)

Prepared according to the general procedure. Flash chromatography (silica gel, pentane/EtOAc) provided the desired product in 86% yield. ^{1}H NMR (400 MHz, $CDCl_3$) δ 8.04 – 7.95 (m, 4H), 7.79 – 7.71 (m, 4H), 6.49 (d, *J* = 38.6 Hz, 1H), 2.63 (s, 3H), 2.62 (d, *J* = 2.7 Hz, 3H). ^{13}C NMR (101 MHz, $CDCl_3$) δ 197.6, 197.3, 157.8 (d, *J* = 262.1 Hz), 137.9 (d, *J* = 3.2 Hz), 136.6 (d, *J* = 27.6 Hz), 137.6, 136.1 (d, *J* = 2.1 Hz), 129.4 (d, *J* = 8.4 Hz), 128.9, 124.7 (d, *J* = 7.6 Hz), 107.5 (d, *J* = 9.9 Hz), 26.8, 26.7. ^{19}F NMR (377 MHz, $CDCl_3$) δ -111.3. HRMS m/z calcd. for $C_{18}H_{16}FO_2$ [M + H]$^+$ 283.1129; found 283.1130.

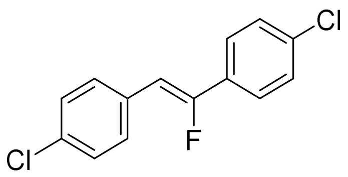

(Z)-4,4'-(1-fluoroethene-1,2-diyl)bis(chlorobenzene) (8f)

Prepared according to the general procedure. Flash chromatography (silica gel, pentane) provided the desired product in 95% yield. ^{1}H NMR (400 MHz, $CDCl_3$) δ 7.62 – 7.51 (m, 4H), 7.43 – 7.31 (m, 4H), 6.25 (d, *J* = 38.9 Hz, 1H). ^{13}C NMR (101 MHz, $CDCl_3$) δ 156.8 (d, *J* = 258.8 Hz), 135.3, 133.3 (d, *J* = 3.6 Hz), 132.0 (d, *J* = 2.9 Hz), 131.2 (d, *J* = 28.4 Hz), 130.3 (d, *J* = 8.3 Hz), 129.1 (d, *J* = 1.7 Hz), 129.0, 105.4 (d, *J* = 10.4 Hz). ^{19}F NMR (377 MHz, $CDCl_3$) δ -113.7. HRMS m/z calcd. for $C_{14}H_9Cl_2F$ $[M]^+$ 266.0060; found 266.0061.

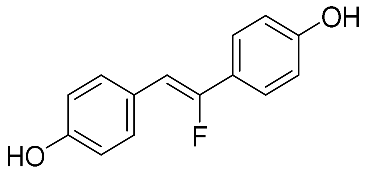

(Z)-4,4'-(1-fluoroethene-1,2-diyl)diphenol (8g)

Prepared according to the general procedure. Flash chromatography (silica gel, pentane/EtOAc) provided the desired product in 99% yield. ^{1}H NMR (500 MHz, d_6-DMSO) δ 9.81 (s, 1H), 9.57 (s, 1H), 7.45 (dd, *J* = 27.2, 8.6 Hz, 4H), 6.79 (dd, *J* = 23.5, 8.5 Hz, 4H), 6.36 (d, *J* = 43.2 Hz, 1H). ^{13}C NMR (126 MHz, d_6-DMSO) δ 158.1, 156.5 (d, *J* = 2.0 Hz), 155.1 (d, *J* = 251.8 Hz), 129.9 (d, *J* = 7.6 Hz), 125.3 (d, *J* = 7.4 Hz), 124.8 (d, *J* = 1.4 Hz), 123.4 (d, *J* = 28.2 Hz), 115.6, 115.5, 103.0 (d, *J* = 9.7 Hz). ^{19}F NMR (471 MHz, d_6-DMSO) δ -117.9. HRMS m/z calcd. for $C_{14}H_{12}FO_2$ $[M + H]^+$ 231.0816; found 231.0812.

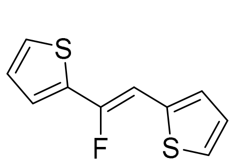

(Z)-2,2'-(1-fluoroethene-1,2-diyl)dithiophene (8h)

Prepared according to the general procedure. Flash chromatography (silica gel, pentane/EtOAc) provided the desired product in 94% yield. ^{1}H NMR (400 MHz, $CDCl_3$) δ 7.34 – 7.28 (m, 3H), 7.19 – 7.06 (m, 1H), 7.06 – 6.97 (m, 2H), 6.47 (d, *J* = 37.6 Hz, 1H). ^{13}C NMR (101 MHz, $CDCl_3$) δ 151.7 (d, *J* = 254.1 Hz), 135.7 (d, *J* = 4.7 Hz), 135.5 (d, *J* = 32.7 Hz), 127.9, 127.1 (d, *J* = 4.0 Hz), 127.0, 126.3 (d, *J* = 9.4 Hz), 125.9, 124.7 (d, *J* = 3.5 Hz),

100.1 (d, *J* = 14.0 Hz). ^{19}F NMR (377 MHz, $CDCl_3$) δ -105.7. HRMS m/z calcd. for $C_{10}H_8FS_2$ [M + H]$^+$ 211.0046; found 211.0044.

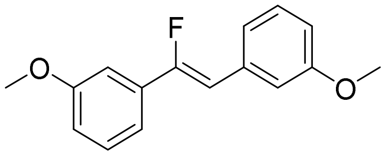

(Z)-3,3'-(1-fluoroethene-1,2-diyl)bis(methoxybenzene) (8i)

Prepared according to the general procedure. Flash chromatography (silica gel, pentane/EtOAc) provided the desired product in 99% yield. ^{1}H NMR (500 MHz, $CDCl_3$) δ 7.35 – 7.27 (m, 2H), 7.26 – 7.19 (m, 3H), 7.19 – 7.15 (m, 1H), 6.92 (ddd, *J* = 8.2, 2.5, 0.8 Hz, 1H), 6.84 (ddd, *J* = 8.2, 2.6, 0.8 Hz, 1H), 6.29 (d, *J* = 39.2 Hz, 1H), 3.86 (s, 3H), 3.85 (s, 3H). ^{13}C NMR (126 MHz, $CDCl_3$) δ 159.9 (d, *J* = 1.9 Hz), 159.8, 157.8 (d, *J* = 259.1 Hz), 135.0 (d, *J* = 2.8 Hz), 134.3 (d, *J* = 27.9 Hz), 129.8, 129.6, 121.8 (d, *J* = 7.4 Hz), 117.0 (d, *J* = 7.4 Hz), 114.9, 114.3 (d, *J* = 8.9 Hz), 113.5 (d, *J* = 1.8 Hz), 110.0 (d, *J* = 8.0 Hz), 106.2 (d, *J* = 10.2 Hz), 55.5, 55.4. ^{19}F NMR (282 MHz, $CDCl_3$) δ -113.3. HRMS m/z calcd. for $C_{16}H_{16}FO_2$ [M + H]$^+$ 259.1129; found 259.1128.

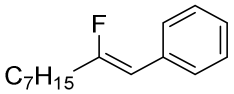

(Z)-(2-fluoronon-1-en-1-yl)benzene (10a)

Prepared according to the general procedure. Flash chromatography (silica gel, pentane) provided the desired product in 94% yield. ^{1}H NMR (400 MHz, $CDCl_3$) δ 7.49 – 7.44 (m, 2H), 7.35 – 7.28 (m, 2H), 7.23 – 7.15 (m, 1H), 5.46 (d, *J* = 39.6 Hz, 1H), 2.32 (dt, *J* = 18.2, 7.5 Hz, 2H), 1.59 (dd, *J* = 14.8, 7.3 Hz, 2H), 1.44 – 1.23 (m, 8H), 0.89 (t, *J* = 6.8 Hz, 3H). ^{13}C NMR (101 MHz, $CDCl_3$) δ 161.5 (d, *J* = 266.8 Hz), 134.1 (d, *J* = 2.2 Hz), 128.5, 128.4 (d, *J* = 7.4 Hz), 126.7 (d, *J* = 2.0 Hz), 105.8 (d, *J* = 8.8 Hz), 33.2 (d, *J* = 26.4 Hz), 31.9, 29.2, 29.1, 26.5, 22.8, 14.2. ^{19}F NMR (377 MHz, $CDCl_3$) δ -100.7. HRMS m/z calcd. for $C_{15}H_{22}F$ [M + H]$^+$ 221.1700; found 221.1698.

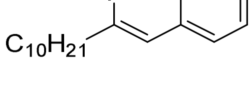

(Z)-(2-fluorododec-1-en-1-yl)benzene (10b)

Prepared according to the general procedure. Flash chromatography (silica gel, pentane) provided the desired product in 98% yield. ^1H NMR (400 MHz, $CDCl_3$) δ 7.54 – 7.42 (m, 2H), 7.37 – 7.27 (m, 2H), 7.22 – 7.13 (m, 1H), 5.46 (d, *J* = 39.6 Hz, 1H), 2.32 (dt, *J* = 18.2, 7.5 Hz, 2H), 1.65 – 1.55 (m, 2H), 1.45 – 1.23 (m, 14H), 0.89 (t, *J* = 6.7 Hz, 3H). ^{13}C NMR (101 MHz, $CDCl_3$) δ 161.5 (d, *J* = 266.7 Hz), 134.1 (d, *J* = 2.2 Hz), 128.5, 128.4 (d, *J* = 7.4 Hz), 105.8 (d, *J* = 8.9 Hz), 33.2 (d, *J* = 26.4 Hz), 33.1, 32.1, 29.7, 29.7, 29.5, 29.5, 29.1, 26.5, 22.8, 14.3. ^{19}F NMR (377 MHz, $CDCl_3$) δ -100.6. HRMS m/z calcd. for $C_{18}H_{28}F$ $[M + H]^+$ 263.2170; found 263.2168.

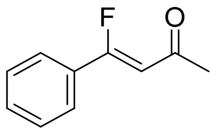

(Z)-4-fluoro-4-phenylbut-3-en-2-one (10c)

Prepared according to the general procedure. Flash chromatography (silica gel, pentane/EtOAc) provided the desired product in 96% yield. ^1H NMR (400 MHz, $CDCl_3$) δ 7.70 – 7.64 (m, 2H), 7.55 – 7.39 (m, 3H), 6.07 (d, *J* = 38.7 Hz, 1H), 2.51 (d, *J* = 4.1 Hz, 3H). ^{13}C NMR (101 MHz, $CDCl_3$) δ 196.7 (d, *J* = 1.3 Hz), 165.8 (d, *J* = 273.8 Hz), 131.8, 130.6 (d, *J* = 27.5 Hz), 129.1 (d, *J* = 1.8 Hz), 126.0 (d, *J* = 8.1 Hz), 107.3 (d, *J* = 10.1 Hz), 31.6 (d, *J* = 6.5 Hz). ^{19}F NMR (377 MHz, $CDCl_3$) δ -96.3. HRMS m/z calcd. for $C_{10}H_{10}FO$ $[M + H]^+$ 165.0710; found 165.0708.

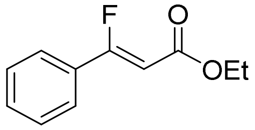

ethyl (Z)-3-fluoro-3-phenylacrylate (10d)

Prepared according to the general procedure. Flash chromatography (silica gel, pentane/EtOAc) provided the desired product in 98% yield. ^1H NMR (500 MHz, $CDCl_3$) δ 7.69 – 7.60 (m, 2H), 7.52 – 7.38 (m, 3H), 5.90 (d, *J* = 33.3 Hz, 1H), 4.26 (q, *J* = 7.1 Hz, 2H), 1.33 (t, *J* = 7.1 Hz, 3H). ^{13}C NMR (126 MHz, $CDCl_3$) δ 167.5, 164.8 (d, *J* = 139.7 Hz), 131.6, 130.8 (d, *J* = 26.2 Hz), 129.0 (d, *J* = 1.6 Hz), 125.8 (d, *J* = 7.9 Hz), 97.3 (d, *J* = 6.8 Hz), 60.6, 14.4. ^{19}F NMR (377 MHz, $CDCl_3$) δ -96.1. HRMS m/z calcd. for $C_{11}H_{12}FO_2$ $[M + H]^+$ 195.0816; found 195.0817.

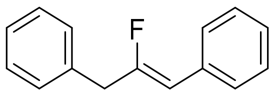

(Z)-(2-fluoroprop-1-ene-1,3-diyl)dibenzene (10e)

Prepared according to the general procedure. Flash chromatography (silica gel, pentane) provided the desired product in 91% yield. ^{1}H NMR (400 MHz, $CDCl_3$) δ 7.47 – 7.42 (m, 2H), 7.37 – 7.15 (m, 8H), 5.50 (d, *J* = 38.8 Hz, 1H), 3.63 (d, *J* = 17.0 Hz, 2H). ^{13}C NMR (101 MHz, $CDCl_3$) δ 159.6 (d, *J* = 267.3 Hz), 136.3 (d, *J* = 1.0 Hz), 133.7 (d, *J* = 2.6 Hz), 129.1, 128.8, 128.5, 128.5, 127.1, 127.1 (d, *J* = 2.1 Hz), 107.5 (d, *J* = 8.2 Hz), 39.8 (d, *J* = 27.9 Hz). ^{19}F NMR (376 MHz, $CDCl_3$) δ -100.1. HRMS m/z calcd. for $C_{15}H_{14}F$ $[M + H]^+$ 213.1074; found 213.1070.

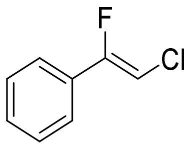

(Z)-(2-chloro-1-fluorovinyl)benzene (10f)

Prepared according to the general procedure. Flash chromatography (silica gel, pentane) provided the desired product in 89% yield. ^{1}H NMR (400 MHz, $CDCl_3$) δ 7.52 – 7.45 (m, 2H), 7.41 – 7.35 (m, 3H), 6.11 (d, *J* = 24.3 Hz, 1H). ^{13}C NMR (101 MHz, $CDCl_3$) δ 158.35 (d, *J* = 253.9 Hz), 130.43 (d, *J* = 26.2 Hz), 129.92, 128.89 (d, *J* = 1.5 Hz), 124.21 (d, *J* = 6.1 Hz), 98.03 (d, *J* = 20.5 Hz). ^{19}F NMR (377 MHz, $CDCl_3$) δ -113.6.

(Z)-5-(1-fluoro-2-(3,4,5-trimethoxyphenyl)vinyl)-2-methoxyphenol (10g)

Prepared according to the general procedure. Flash chromatography (silica gel, pentane/EtOAc, 7:3) provided the desired product in 32% yield. ^{1}H NMR (400 MHz, $CDCl_3$) δ 7.19 (d, *J* = 2.2 Hz, 1H), 7.15 (dd, *J* = 8.4, 2.2 Hz, 1H), 6.89 – 6.86 (m, 3H), 6.10 (d, *J* = 39.1 Hz, 1H), 5.67 (s, 1H), 3.93 (s, 3H), 3.90 (s, 6H), 3.87 (s, 3H). ^{13}C NMR (101 MHz, $CDCl_3$) δ 156.68 (d, *J* = 257.1 Hz), 153.13, 147.38, 145.68 (d, *J* = 2.4 Hz), 129.53 (d, *J* = 2.8 Hz), 126.33 (d, *J* = 28.6 Hz), 124.23, 116.57 (d, *J* = 8.2 Hz), 110.51, 106.05 (d, *J* = 8.5 Hz), 104.56 (d, *J* = 10.2 Hz), 60.95, 56.16, 56.11, 56.04. ^{19}F NMR (376 MHz, $CDCl_3$) δ -114.1. HRMS m/z calcd. for $C_{18}H_{20}FO_5$ $[M + H]^+$ 335.1289; found 335.1290.

Thio enols: Starting material synthesis

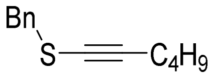

Benzyl(hex-1-yn-1-yl)sulfane (11a)

1-Hexyne (2.0 mL, 17.4 mmol, 1.0 equiv.) was dissolved in diethyl ether (40 mL) and the resulting mixture cooled to -78 ºC. Butyllithium (1.6 M in hexanes, 10.9 mL, 17.4 mmol, 1.0 equiv.) was slowly added dropwise with stirring, and the mixture stirred at -78 ºC for a further 15 minutes. Molecular sulphur (556.8 mg, 17.4 mmol, 1.0 equiv.) was then added portionwise at -78 ºC, and the resulting mixture was stirred for a further hour and allowed to warm to room temperature. The mixture was then cooled to 0 ºC, benzyl bromide (2.1 mL, 17.4 mmol, 1.0 equiv.) was added dropwise, and the resulting mixture was stirred for 16 hours and allowed to warm to room temperature. It was then treated with a sat. aq. solution of sodium hydrogen carbonate (20 mL), the layers were separated and the aqueous layer was extracted with diethyl ether (3 x 10 mL). The combined organic layers were dried over magnesium sulfate, filtered and concentrated under reduced pressure to furnish a yellow oil. Purification by silica gel column chromatography, eluting with hexane, furnished the title compound as a slightly yellow oil (2.7 g, 78% yield). ^{1}H NMR (500 MHz, $CDCl_3$) δ 7.37 – 7.28 (m, 5H), 3.91 (s, 2H), 2.29 (t, J = 7.0 Hz, 2H), 1.51 – 1.41 (m, 2H), 1.40 – 1.32 (m, 2H), 0.90 (t, J = 7.3 Hz, 3H).

These data are in good agreement with the literature values.[6]

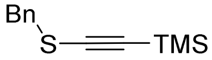

Benzylthioethynyl trimethylsilane

Trimethylsilyl acetylene (5.5 mL, 39.0 mmol, 1.0 equiv.) was dissolved in diethyl ether (50 mL) and the resulting mixture was cooled to -78 ºC. Butyllithium (1.6 M in hexanes, 24.0 mL, 39.0 mmol, 1.0 equiv.) was slowly added dropwise with stirring, and the mixture was stirred at -78 ºC for a further 15 minutes. Molecular sulphur (1.25 g, 39 mmol, 1.0 equiv.) was then added portionwise at -78 ºC, and the resulting mixture was stirred for a further hour and allowed to warm to room temperature, after which the sulphur had completely dissolved to yield an orange colour. The mixture was then cooled to 0 ºC, benzyl bromide (4.6 mL, 39.0 mmol, 1.0 equiv.) was added dropwise, and the resulting mixture was stirred for 16 hours and allowed to warm to room temperature. The resulting turbid mixture was concentrated under

reduced pressure to yield a yellow paste, which was extracted with hexane (30 mL) and filtered. This procedure was repeated three times, and the combined hexane layers were concentrated under reduced pressure to furnish the title compound (orange oil, 7.7 g, 89% yield). The material obtained was used in the next synthetic step without any further purification. ^{1}H NMR (500 MHz, $CDCl_3$) δ 7.35-7.30 (m, 5H), 3.94 (s, 2H), 0.14 (s, 9H).
These data are in good agreement with the literature values.[7]

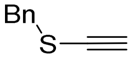

Benzyl ethynyl sulfane (11b)

Benzylthioethynyl trimethylsilane (4.0 g, 18.1 mmol, 1.0 equiv.) was dissolved in a 3:1 mixture of tetrahydrofuran and methanol (40 mL), and tetrabutylammonium fluoride trihydrate (33.0 mg, 0.104 mmol, 0.006 equiv.) was added in one portion. The resulting mixture was stirred at room temperature for 16 hours. It was then concentrated under reduced pressure. The residue was taken up in dichloromethane (30 mL), washed with brine (3 × 10 mL), dried over magnesium sulfate, filtered and concentrated under reduced pressure. The title compound was furnished as a light orange oil (2.48 g, 92% yield). The material obtained was used in the next synthetic step without any further purification. ^{1}H NMR (500 MHz, $CDCl_3$) δ 7.35-7.28 (m, 5H), 3.96 (s, 2H), 2.82 (s, 1H).
These data are in good agreement with the literature values.[7]

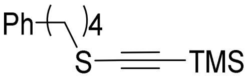

4-Phenylbutyl-thioethynyl trimethylsilane

Trimethylsilyl acetylene (3.5 mL, 24.9 mmol, 1.1 equiv.) was dissolved in diethyl ether (30 mL) and the resulting mixture was cooled to -78 ºC. Butyllithium (1.6 M in hexanes, 15.6 mL, 24.9 mmol, 1.1 equiv.) was slowly added dropwise with stirring, and the mixture was stirred at -78 ºC for a further 15 minutes. Molecular sulphur (797 mg, 24.9 mmol, 1.1 equiv.) was then added portionwise at -78 ºC, and the resulting mixture was stirred for a further hour and allowed to warm to room temperature, after which the sulphur had completely dissolved to yield an orange colour. The mixture was then cooled to 0 ºC and a solution of 4-phenylbutyl bromide (4.83 g, 22.7 mmol, 1.0 equiv.) in dry diethyl ether (10 mL) was added dropwise, the resulting mixture was then stirred for 16 hours and allowed to warm to room

temperature. It was then washed with a sat. aq. solution of sodium hydrogen carbonate (2 x 10 mL), brine (10 mL), dried over magnesium sulfate, and filtered and concentrated under reduced pressure. The material obtained was purified by silica gel flash column chromatography, eluting with hexane, to furnish the title compound as a colourless oil (5.3 g, 90% yield). ^{1}H NMR (500 MHz, $CDCl_3$) δ 7.30 – 7.27 (m, 2H), 7.20 – 7.18 (m, 3H), 2.74 (t, *J* = 6.7, 2H), 2.65 (t, *J* = 7.1 2H), 1.79 – 1.75 (m, 4H), 0.16 (s, 9H).
These data are in good agreement with the literature values.[8]

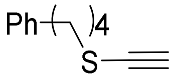

4-Phenylbutyl ethynyl sulfane (11c)
4-Phenylbutyl-thioethynyl trimethylsilane (500 mg, 1.9 mmol, 1.0 equiv.) was dissolved in a 3:1 mixture of tetrahydrofuran and methanol (5 mL), and tetrabutylammonium fluoride trihydrate (48.0 mg, 0.152 mmol, 0.08 equiv.) was added in one portion. The resulting mixture was stirred at room temperature for 16 hours. It was then concentrated under reduced pressure, the residue taken up in dichloromethane (10 mL) and the resulting solution washed with brine (3 × 5 mL), dried over magnesium sulfate, filtered and concentrated under reduced pressure, to furnish a yellow oil. Purification by silica gel column chromatography, eluting with hexane, afforded the title compound as a colourless oil (298 mg, 82% yield); ^{1}H NMR (500 MHz, $CDCl_3$) δ 7.31 - 7.28 (m, 2H), 7.22 - 7.19 (m, 3H), 2.76 (t, *J* = 6.9, 2H), 2.76 (s, 1H), 2.66 (t, *J* = 7.2, 2H), 1.85 - 1.73 (m, 4H).
These data are in good agreement with the literature values.[8]

Thio enols: Hydrofluorination reactions

General procedure for the hydrofluorination reactions
A plastic vial was charged with the corresponding alkyne (1 equiv.) under argon. The indicated gold bifluoride catalyst (0.05 equiv.) was then added. DCM (0.7M) was added, followed by a dropwise addition of triethylamine trihydrofluoride (1.5 mmol, 3 equiv.). The reaction mixture was then stirred at room temperature until total consumption of the starting material. Afterwards, DCM was added followed by a sat. aq. solution of $NaHCO_3$. The two layers were separated and the aqueous phase was extracted 3 times with DCM. The combined organic layers were washed with brine, dried over $MgSO_4$ and concentrated *in vacuo*. The

obtained residue was purified by flash column chromatography (silica gel, pentane was used as eluent).

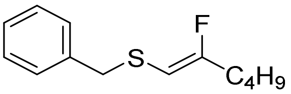

(Z)-benzyl(2-fluorohex-1-en-1-yl)sulfane (12a)

Prepared according to the general procedure. Flash chromatography (silica gel, pentane) provided the desired product in 80% yield. ^{1}H NMR (400 MHz, $CDCl_3$) δ 7.42 – 7.19 (m, 5H), 4.99 (d, *J* = 34.6 Hz, 1H), 3.82 (s, 2H), 2.17 (dt, *J* = 17.6, 7.4 Hz, 2H), 1.48 – 1.37 (m, 2H), 1.34 – 1.22 (m, 2H), 0.88 (t, *J* = 7.3 Hz, 3H). ^{13}C NMR (101 MHz, $CDCl_3$) δ 161.8 (d, *J* = 257.7 Hz), 138.0, 129.0, 128.7, 127.3, 98.1 (d, *J* = 18.2 Hz), 38.2 (d, *J* = 0.8 Hz), 32.2 (d, *J* = 26.2 Hz), 28.3, 22.0, 13.8. ^{19}F NMR (377 MHz, $CDCl_3$) δ -95.9. HRMS m/z calcd. for $C_{13}H_{18}FS$ $[M + H]^+$ 225.1108; found 225.1106.

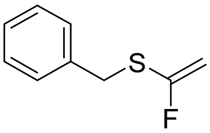

benzyl(1-fluorovinyl)sulfane (12b)

Prepared according to the general procedure. Flash chromatography (silica gel, pentane) provided the desired product in 60% yield. ^{1}H NMR (500 MHz, $CDCl_3$) *δ* 7.34 - 7.24 (m, 5H), 4.91 (dd, *J* = 11.8, *J* = 3.1, 1H), 4.65 *J* = 44.1, *J* = 3.1, 1H), 3.94 (s, 2H). ^{19}F NMR (471 MHz, $CDCl_3$) *δ* -80.9. These data are in good agreement with the literature values.[8]

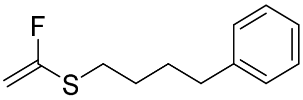

(1-fluorovinyl)(4-phenylbutyl)sulfane (12c)

Prepared according to the general procedure. Flash chromatography (silica gel, pentane) provided the desired product in 62% yield. ^{1}H NMR (500 MHz, $CDCl_3$) *δ* 7.31 – 7.26 (m, 2H), 7.22 – 7.15 (m, 3H), 4.93 (dd, *J* = 11.9, 3.0, 1H), 4.72 (dd, *J* = 44.4, 3.0, 1H), 2.76 (t, *J* = 6.8, 2H), 2.64 (t, *J* = 7.2, 2H), 1.79 – 1.64 (m, 4H). ^{19}F NMR (471 MHz, $CDCl_3$) *δ* -81.6. These data are in good agreement with the literature values.[8]

References

1. F. Nahra, S. R. Patrick, A. Collado, S. P. Nolan, *Polyhedron* **2014**, *In Press*, doi: 10.1016/j.poly.2014.06.017.
2. D. J. Nelson, F. Nahra, S. R. Patrick, D. B. Cordes, A. M. Z. Slawin, Steven P. Nolan, *Organometallics* **2014**, *33,* 3640.
3. a) K. Park, G. Bae, J. Moon, J. Choe, K. H. Song, S. Lee, *J. Org. Chem.* **2010,** *75*, 6244; b) S. Peng, L. Wang, J. Huang, S. Sun, H. Guo, J. Wang, *Adv. Synth. Catal.* **2013**, *355*, 2550; c) N.G. Pschirer, U.H.F. Bunz, *Tetrahedron Lett.* **1999**, *40*, 2481; d) K. Park, G. Bae, A. Park, Y. Kim, J. Choe, K. Ho Song, S. Lee, *Tetrahedron Lett.* **2011**, *52*, 576
4. a) M. Buck, J. M. Chong, *Tetrahedron Lett.* **2001,** *42*, 5825; b) W.-W. Zhang, X.-G. Zhang, J.-H. Li, *J. Org. Chem.* **2010,** *75*, 5259.
5. C. Belger, B. Plietker, *Chem. Commun.* **2012**, *48*, 5419.
6. W. Zheng, Y. Hong, P. Wang, F. Zheng, Y. Zhang, W. Wang, *Tetrahedron Letters* **2013,** *54*, 3643.
7. W. W. Seidel, M. J. Meel, M. Schaffrath, T. Pape, *Eur. J. Org. Chem.* **2007,** *2007*, 3526.
8. Bello, D.; Cormanich, R. A.; O'Hagan, D., *Aust. J. Chem.* **2014**, *In Press*, doi: 10.1071/CH14298 .

NMR Spectra

[[Au(IPr)(NEt$_3$)](HF$_2$)]: ^{1}H NMR

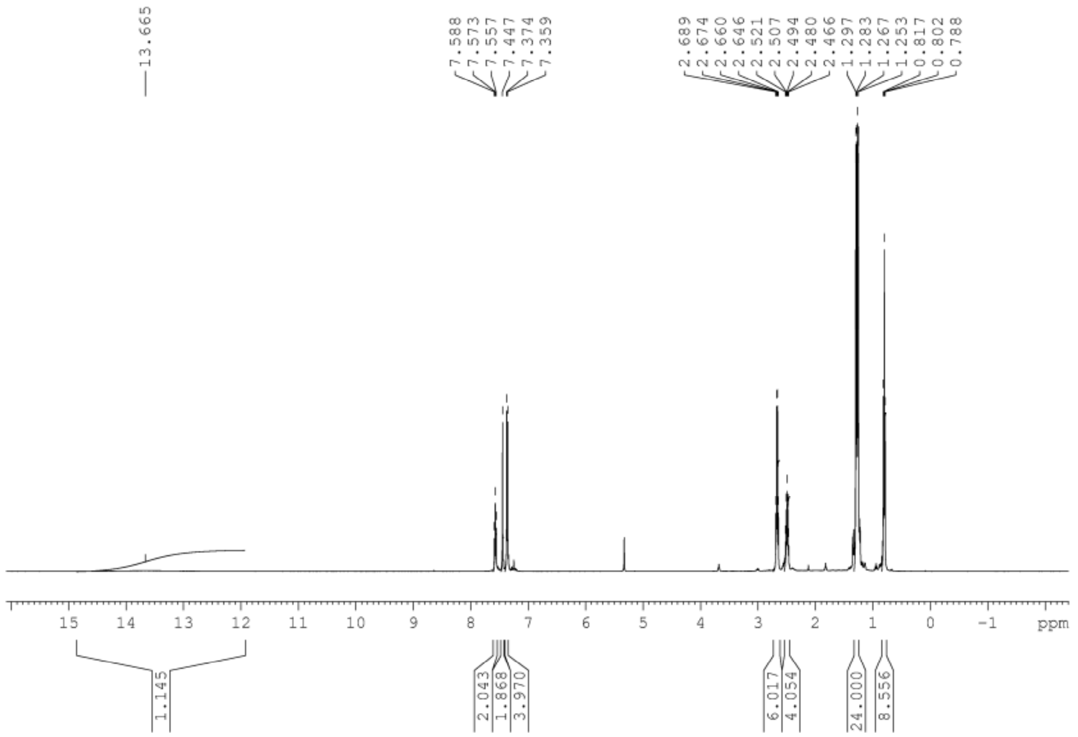

[[Au(IPr)(NEt$_3$)](HF$_2$)]: ^{13}C{^{1}H} NMR

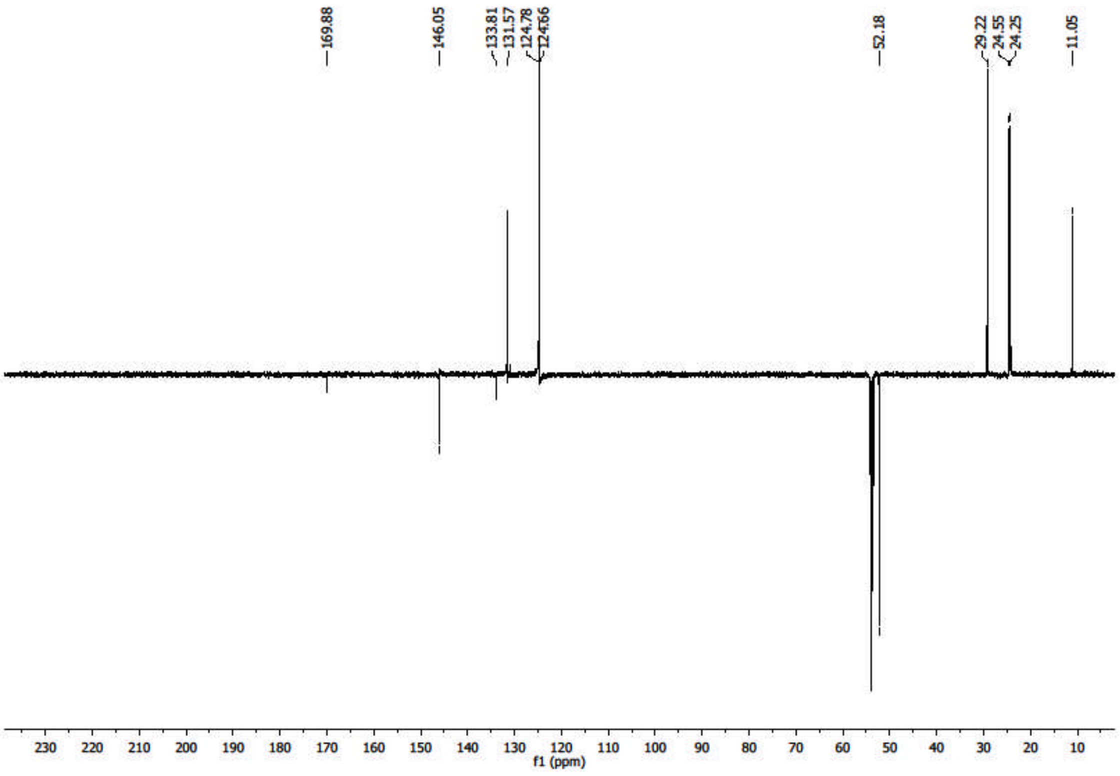

[[Au(IPr)(NEt_3)](HF_2)]: ^{19}F NMR

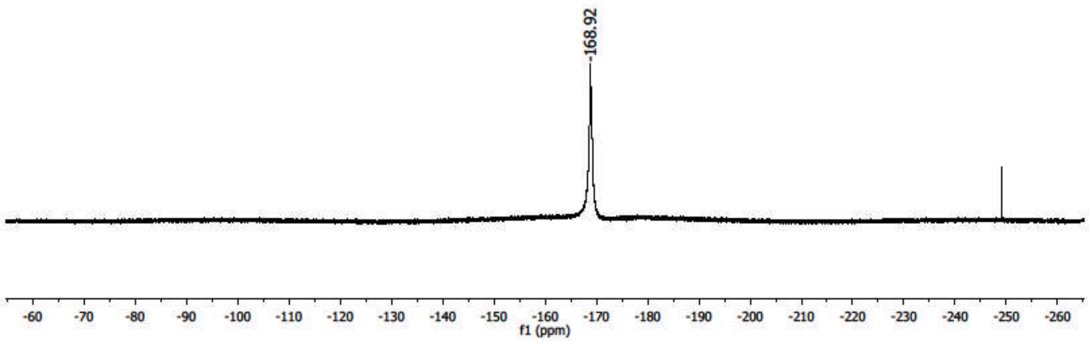

[[Au(SIPr)(NEt_3)](HF_2)]: ^{1}H NMR

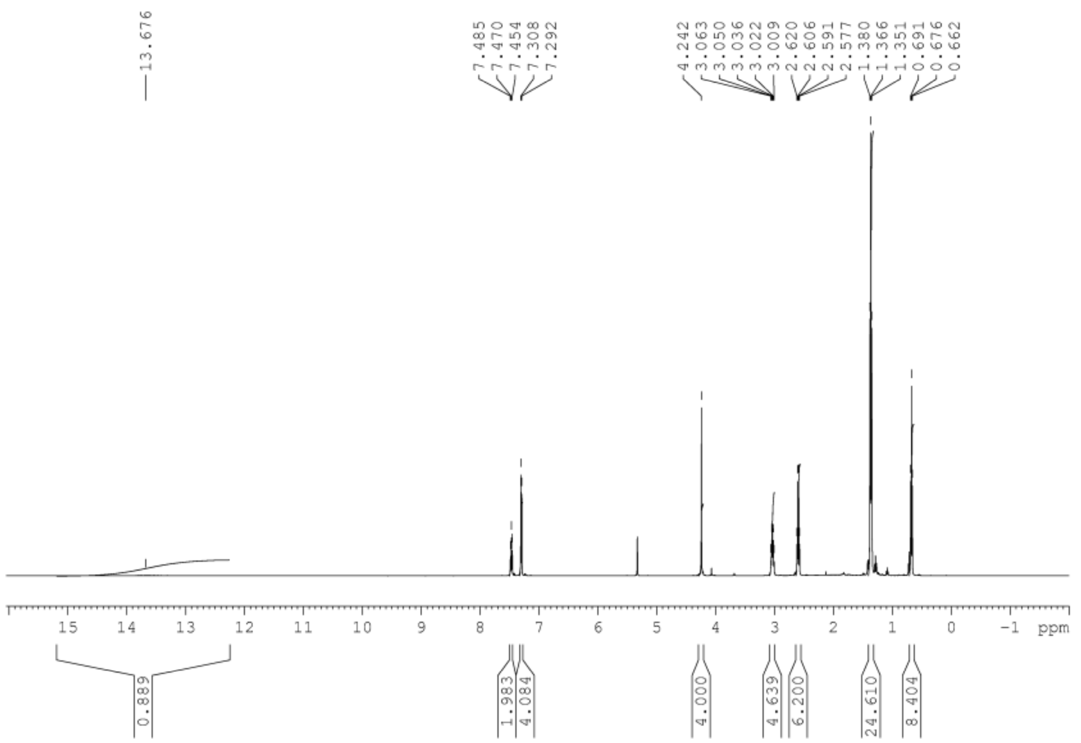

[[Au(SIPr)(NEt_3)](HF_2)]: ^{13}C{^{1}H}-DEPTQ NMR

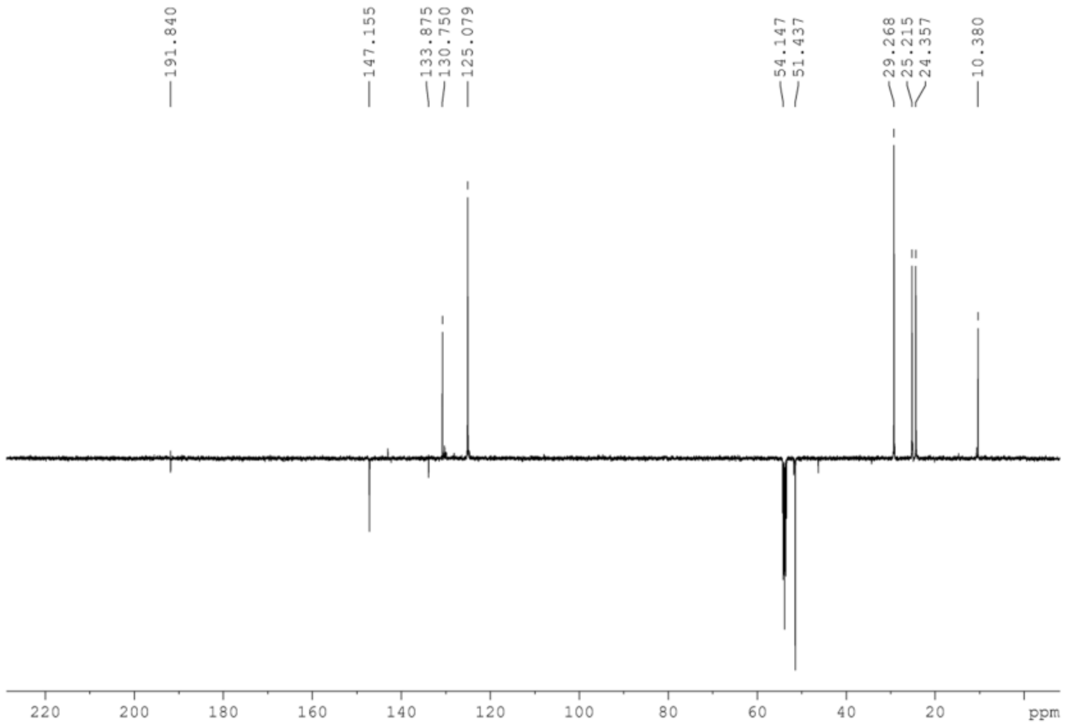

[[Au(SIPr)(NEt$_3$)](HF$_2$)]: ^{19}F NMR

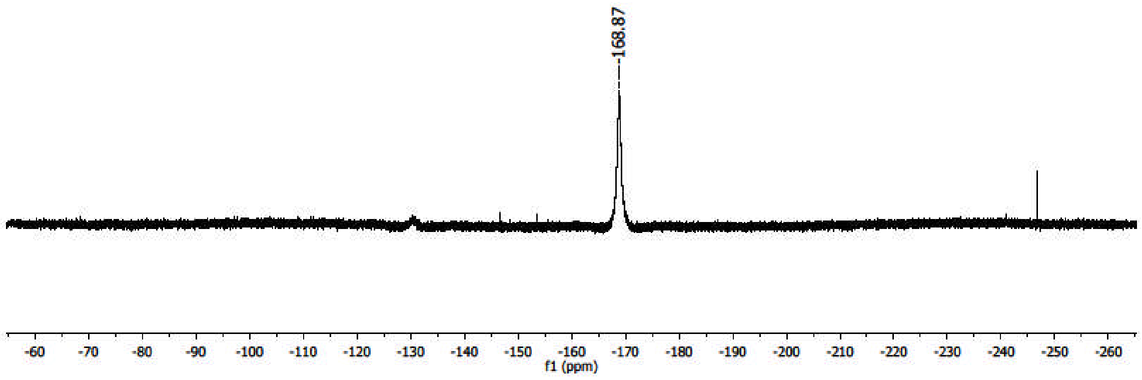

[[Au(IPr^{Cl})(NEt$_3$)](HF$_2$)]: ^{1}H NMR

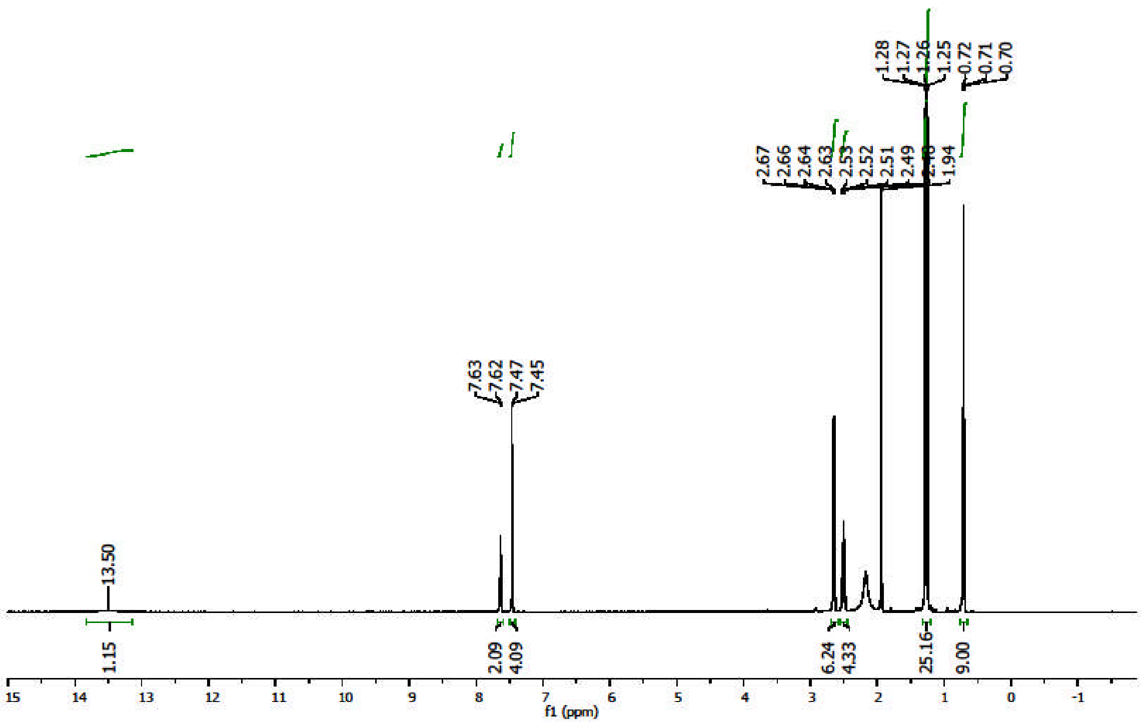

[[Au(IPr^{Cl})(NEt$_3$)](HF$_2$)]: ^{13}C{^{1}H} NMR

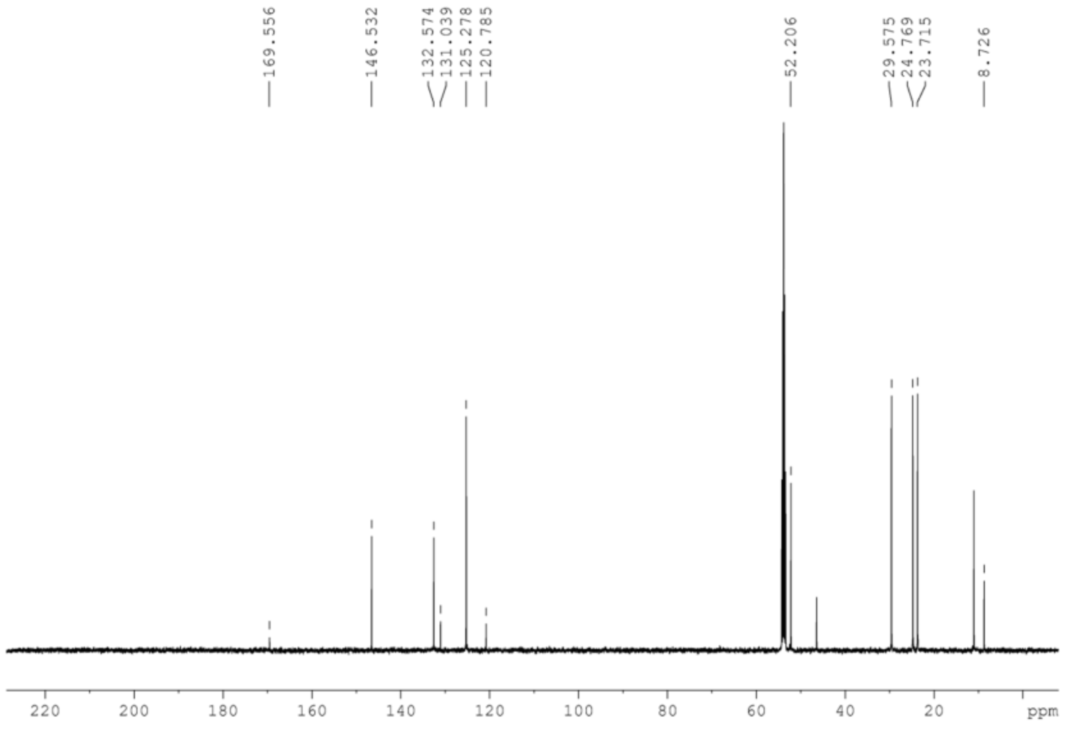

[[Au(IPr^{Cl})(NEt$_3$)](HF$_2$)]: ^{19}F NMR

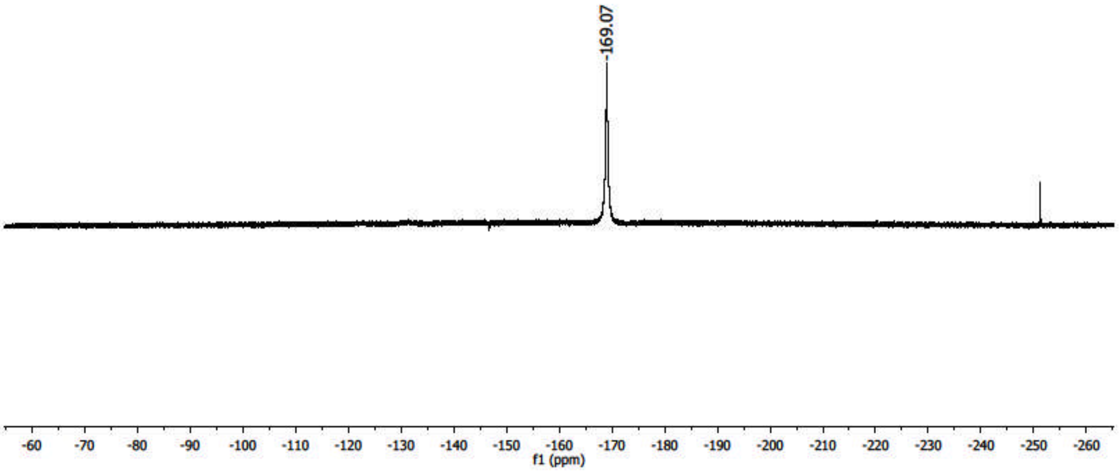

[[Au(IPr^{Me})(NEt$_3$)](HF$_2$)]: ^{1}H NMR

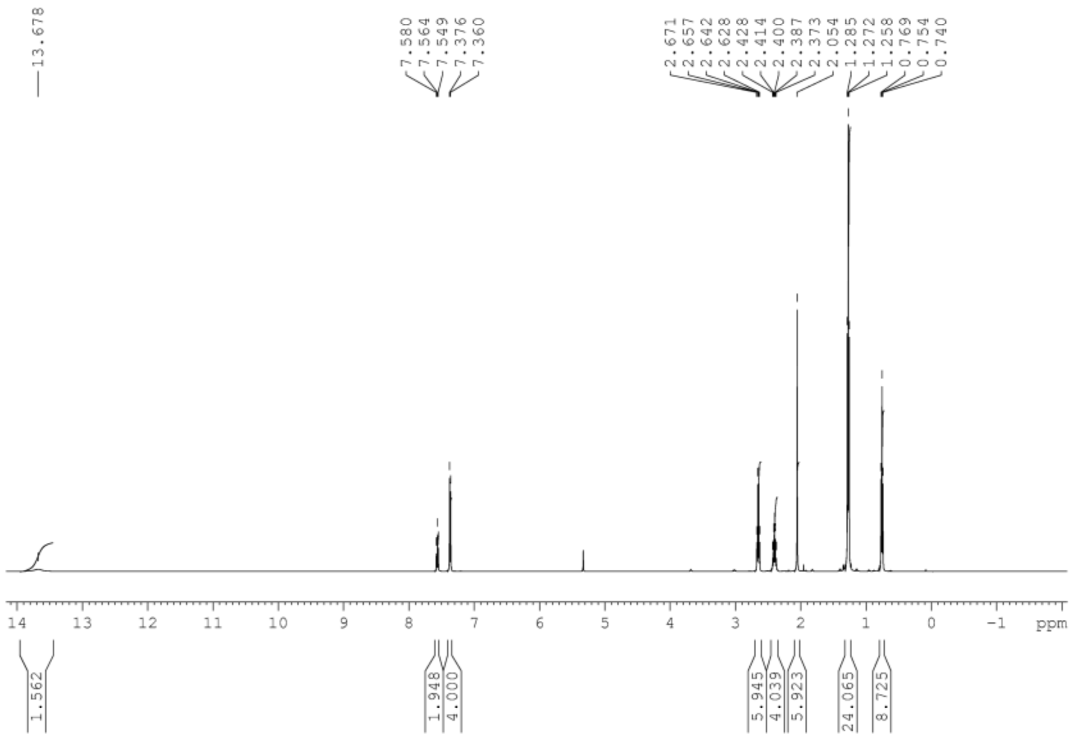

[[Au(IPr^{Me})(NEt$_3$)](HF$_2$)]: ^{13}C{^{1}H} NMR

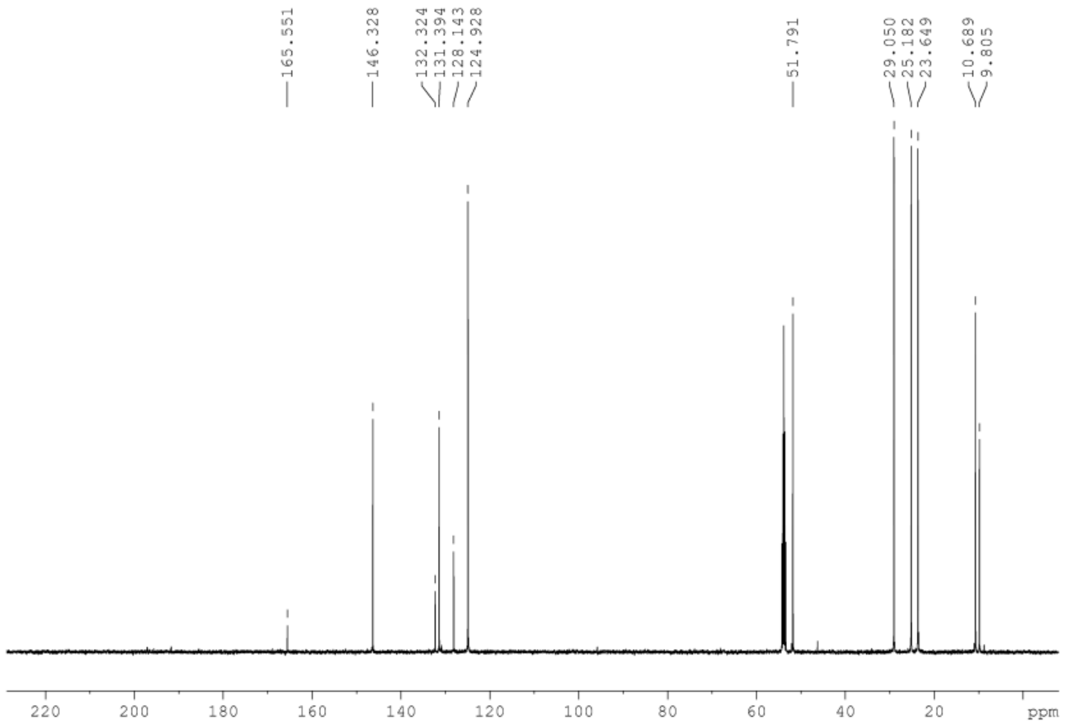

[[Au(IPr^{Me})(NEt$_3$)](HF$_2$)]: ^{19}F NMR

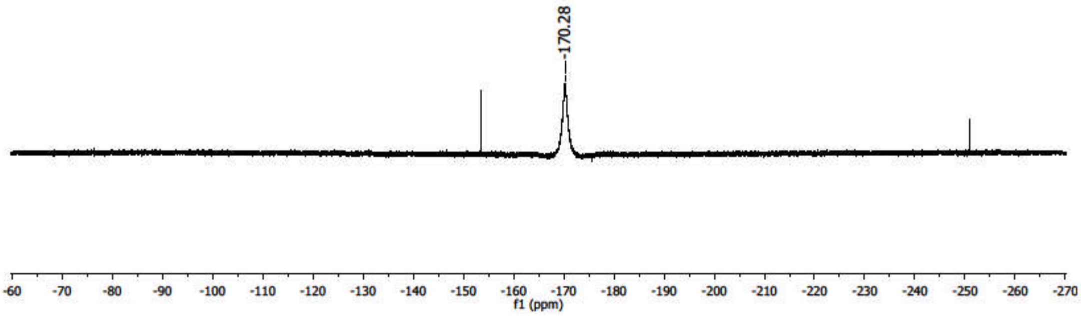

[[Au(IPr*)(NEt$_3$)](HF$_2$)]: ^{1}H NMR

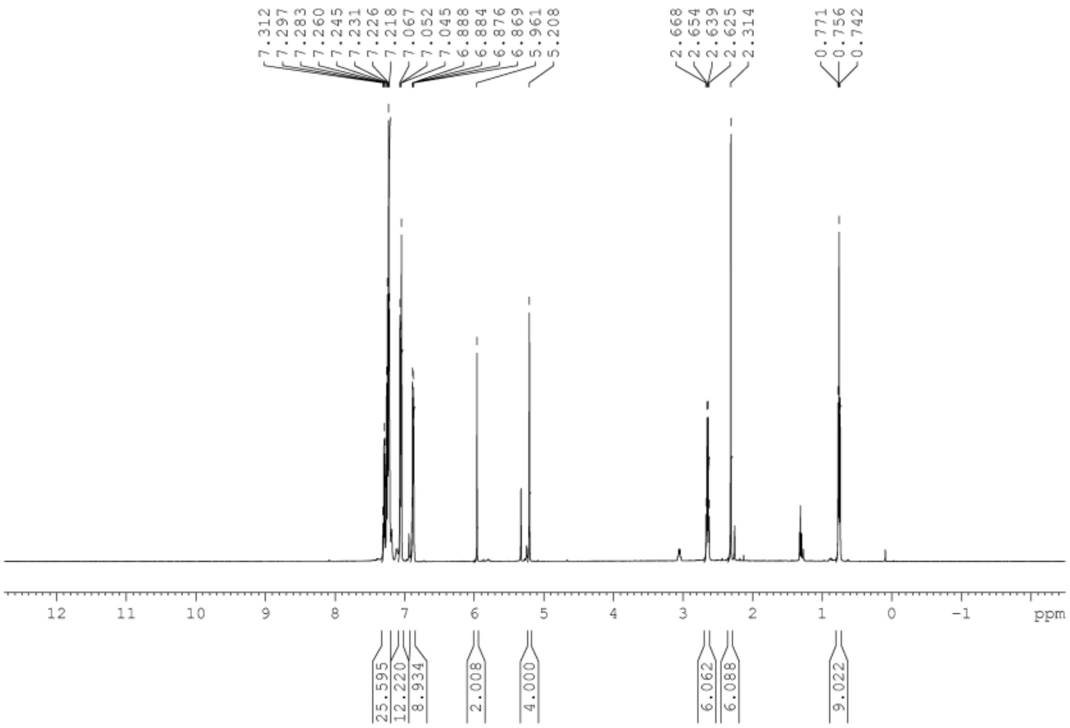

[[Au(IPr*)(NEt$_3$)](HF$_2$)]: ^{13}C{^{1}H} NMR

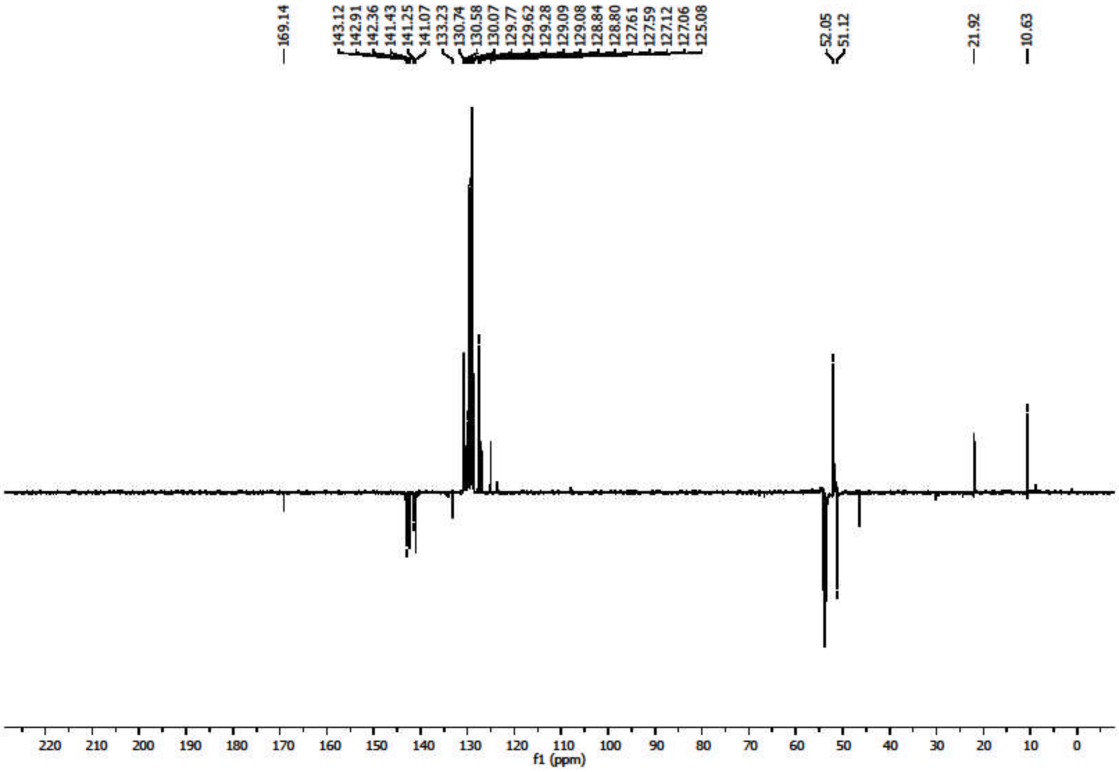

[[Au(IPr*)(NEt$_3$)](HF$_2$)]: ^{19}F NMR

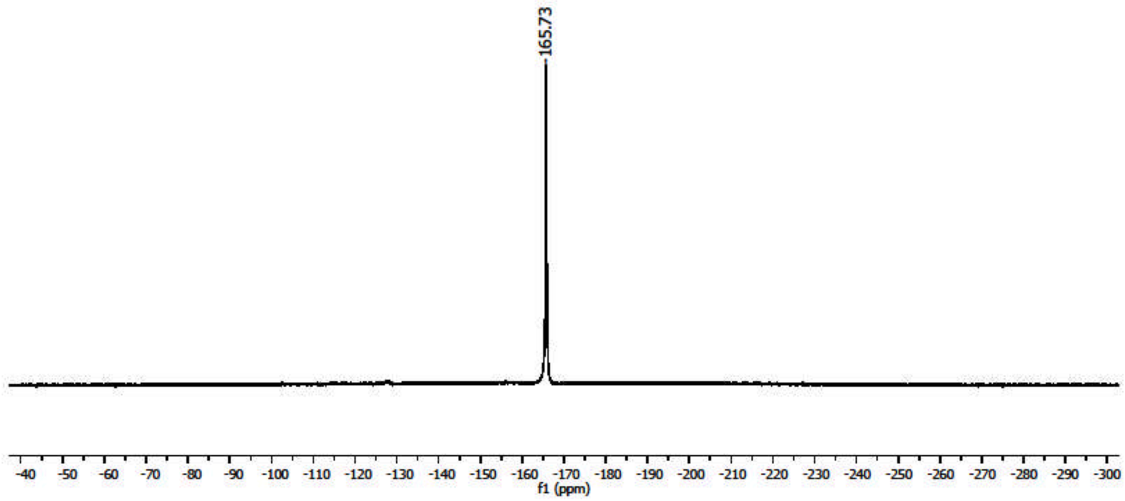

$[[Au(IPr^{*Tol})(NEt_3)](HF_2)]$: ^{1}H NMR

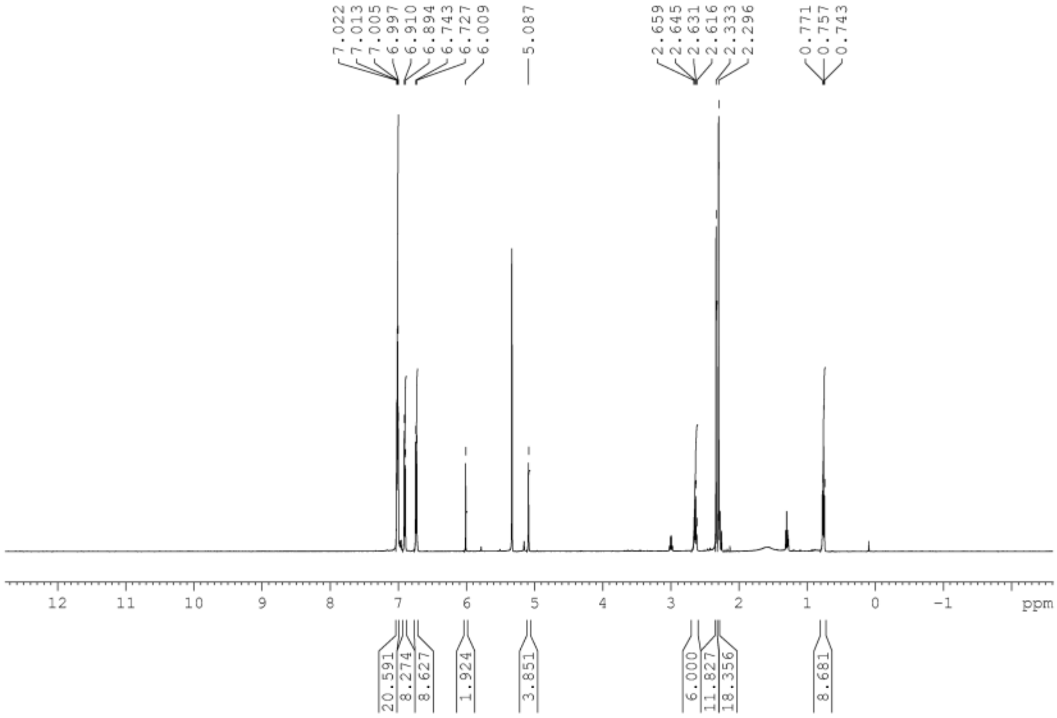

$[[Au(IPr^{*Tol})(NEt_3)](HF_2)]$: $^{13}C\{^{1}H\}$ NMR

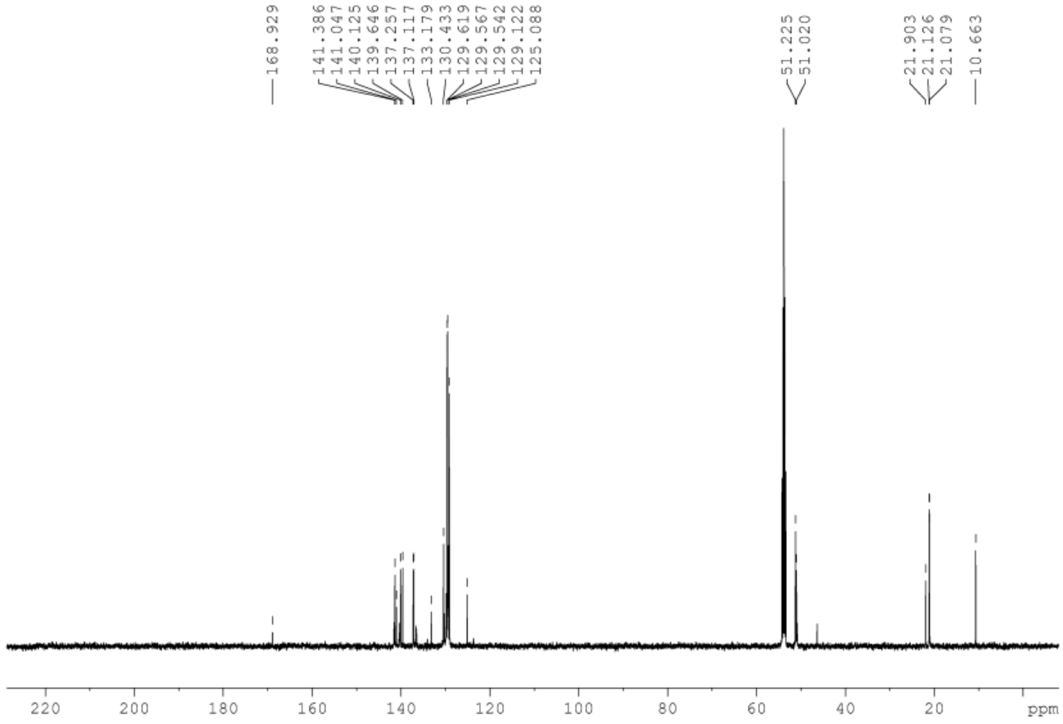

$[[Au(IPr^{*Tol})(NEt_3)](HF_2)]$: ^{19}F NMR

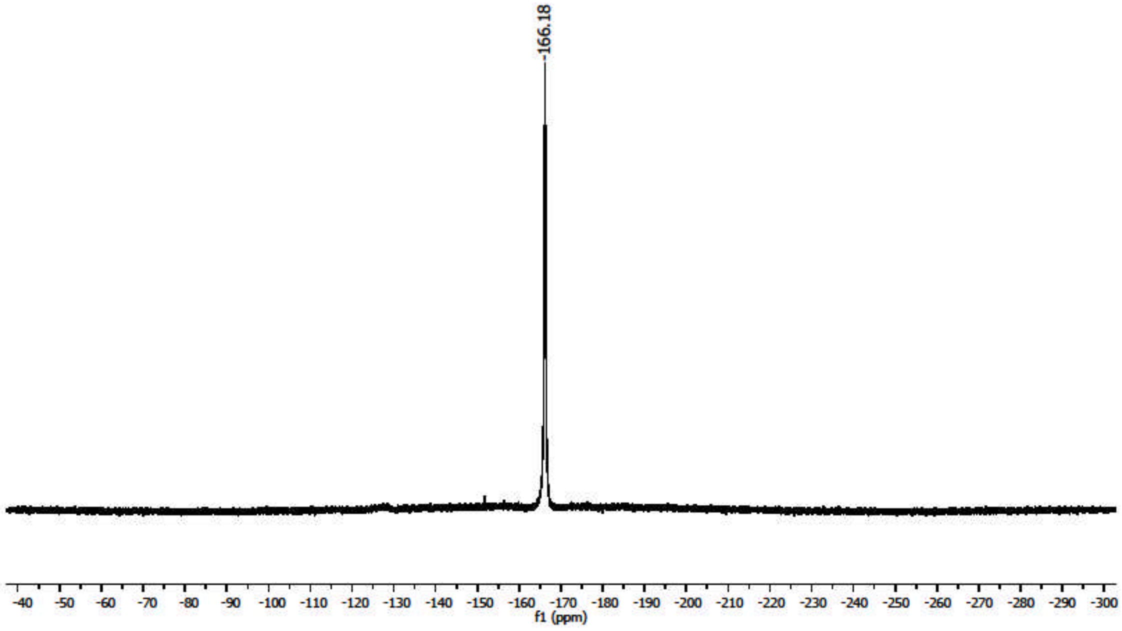

[[Au(IPr)(Py)](HF$_2$)]: ^{1}H NMR

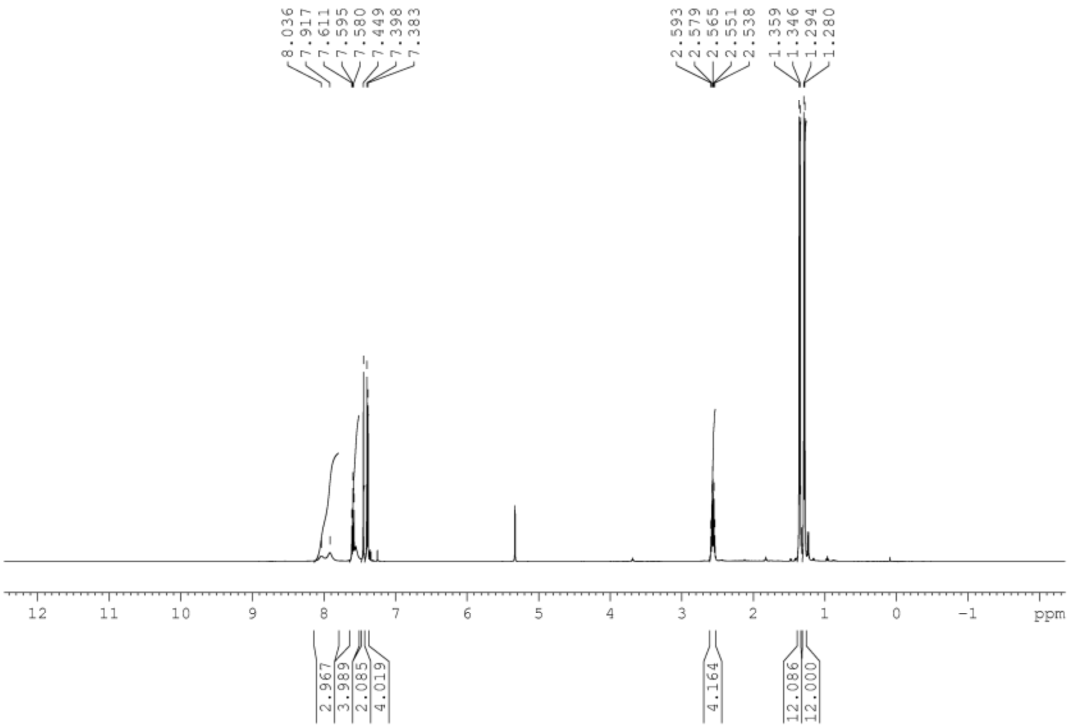

[[Au(IPr)(Py)](HF$_2$)]: ^{13}C{^{1}H} NMR

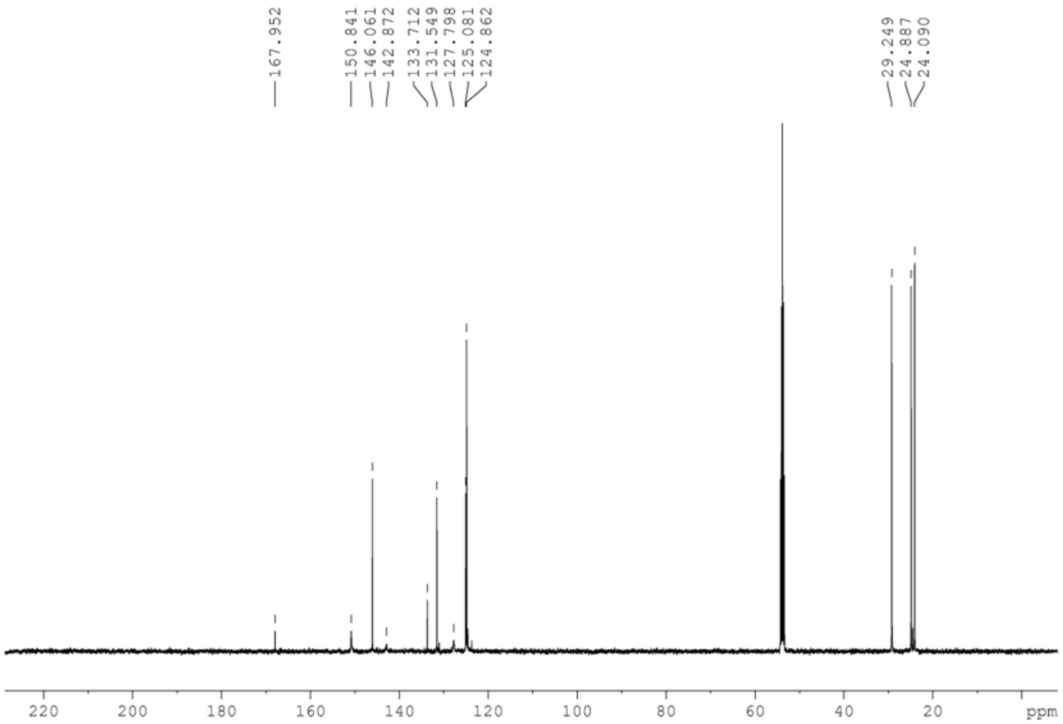

[Au(IPr)(Py)(HF_2)]: ^{19}F NMR

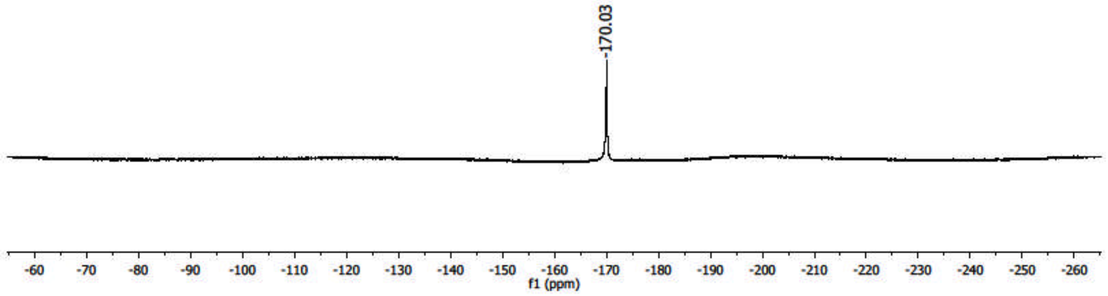

[[Au(IPr*)(Py)](HF_2)]: ^{1}H NMR

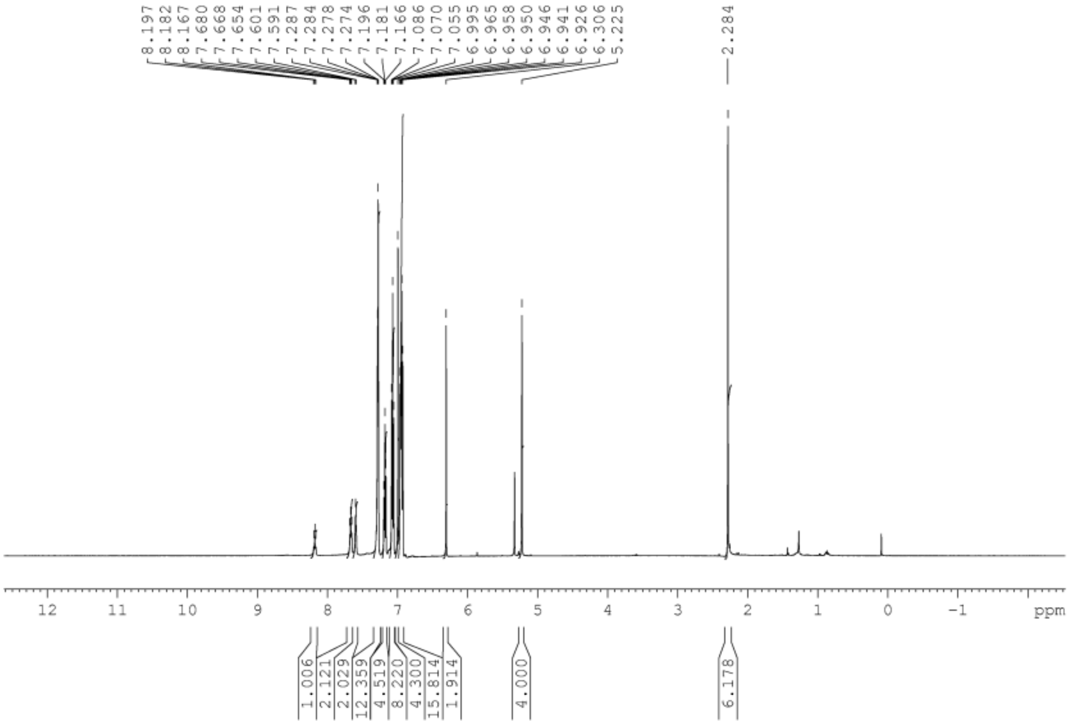

[[Au(IPr*)(Py)](HF_2)]: ^{13}C{^{1}H} NMR

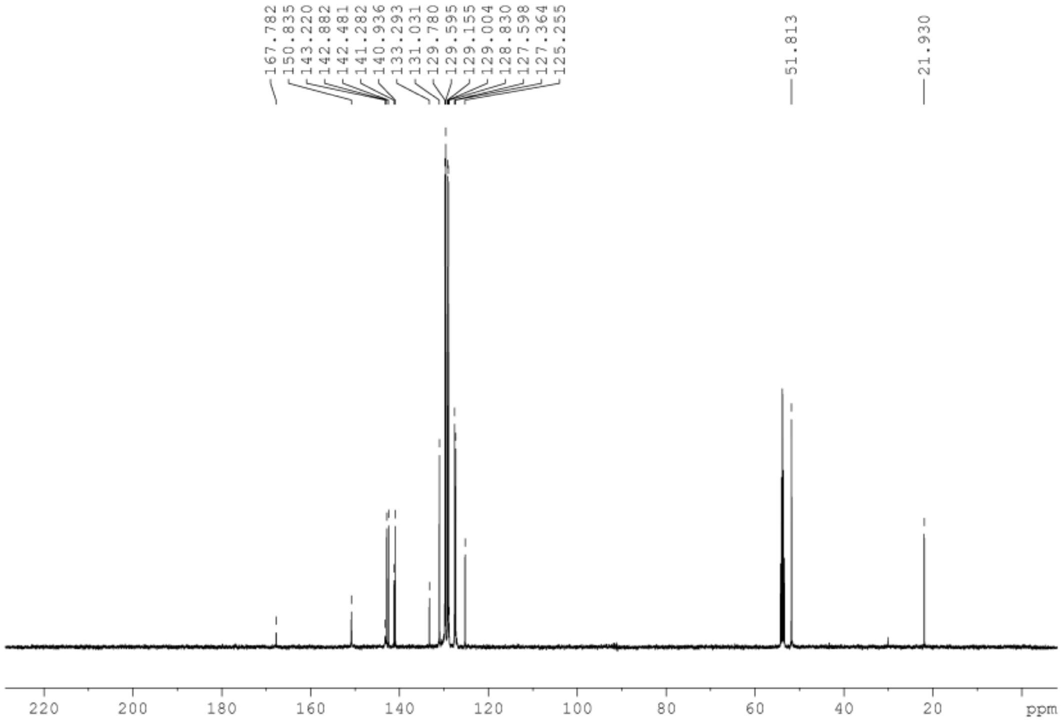

[[Au(IPr*)(Py)](HF$_2$)]: ^{19}F NMR

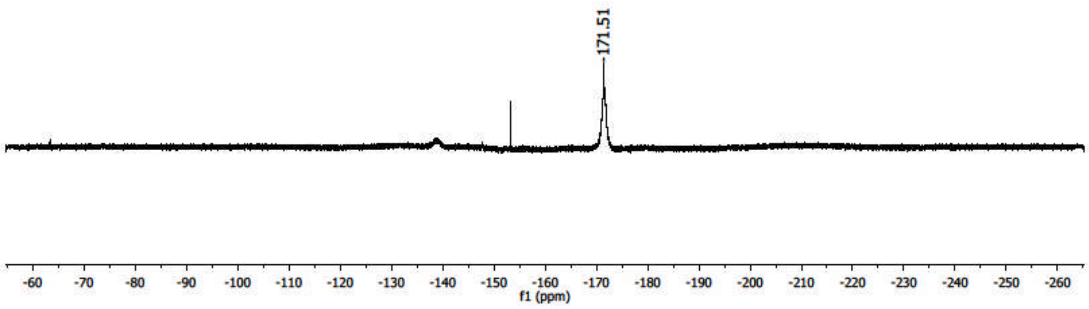

[[Au(IPr)(Se(SIPr))](HF$_2$)]: ^{1}H NMR

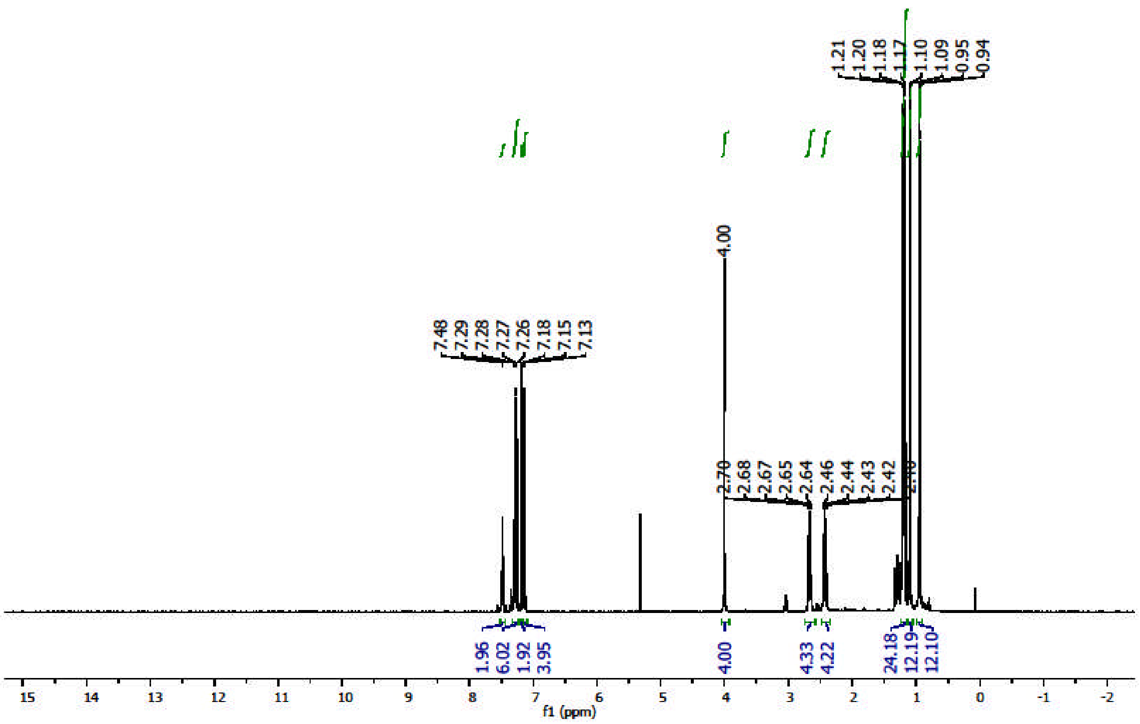

[[Au(IPr)(Se(SIPr))](HF$_2$)]: ^{13}C{^{1}H} NMR

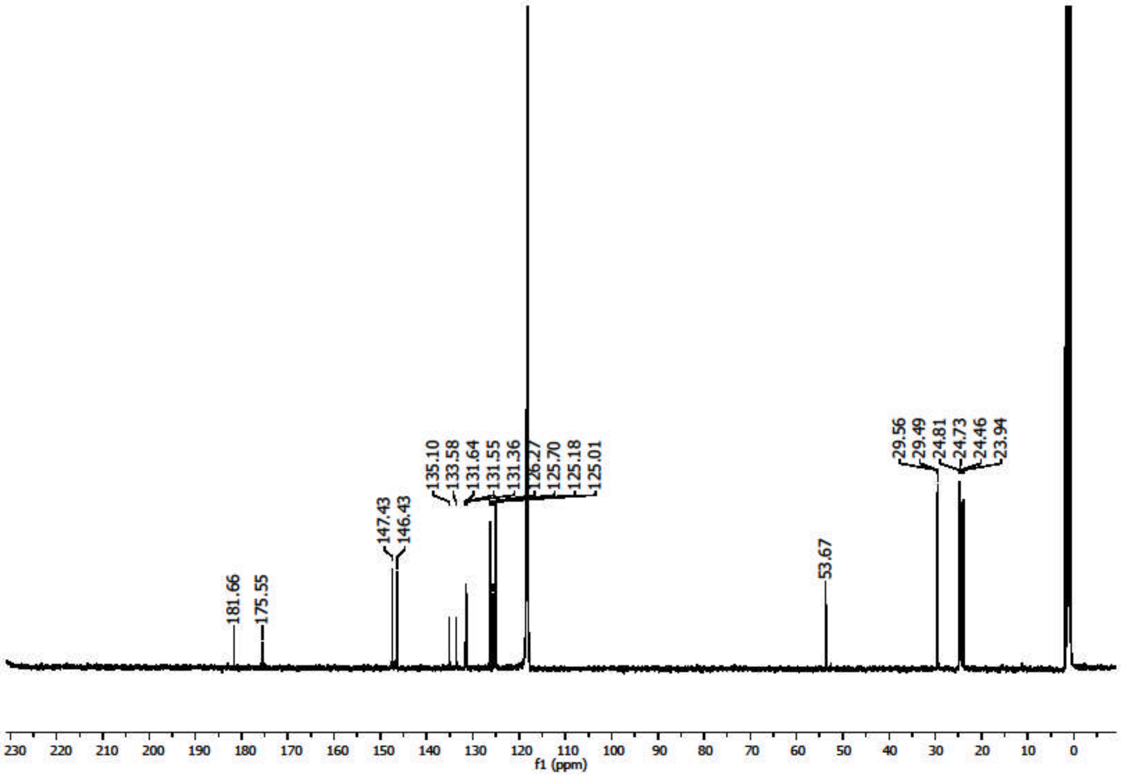

[[Au(IPr)(Se(SIPr))](HF$_2$)]: ^{19}F NMR

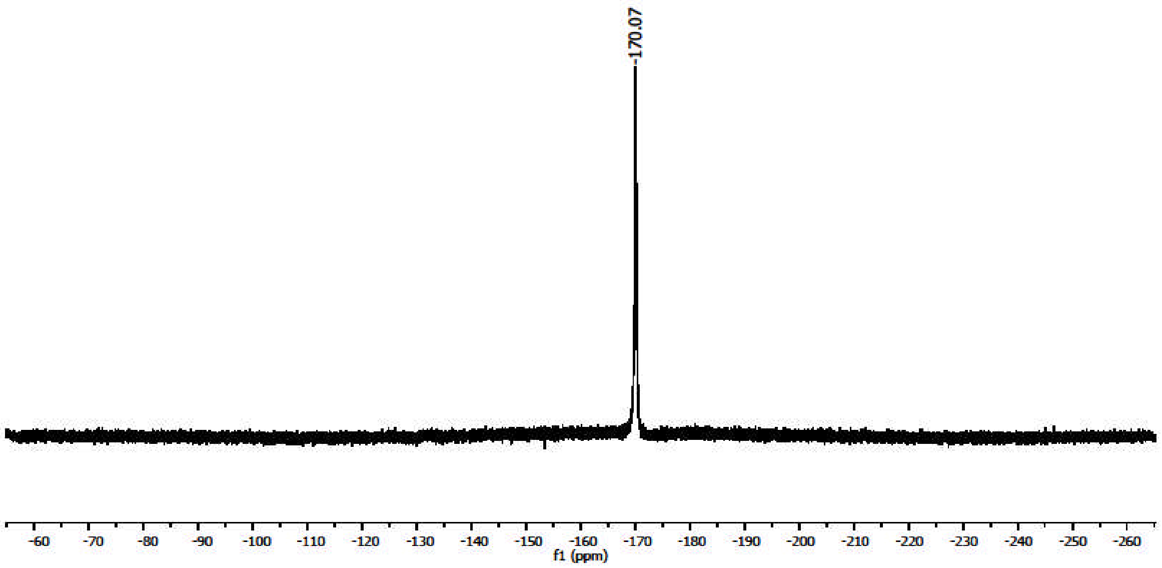

[[Au(IPr)(Se(SIPr))](HF$_2$)]: ^{77}Se NMR

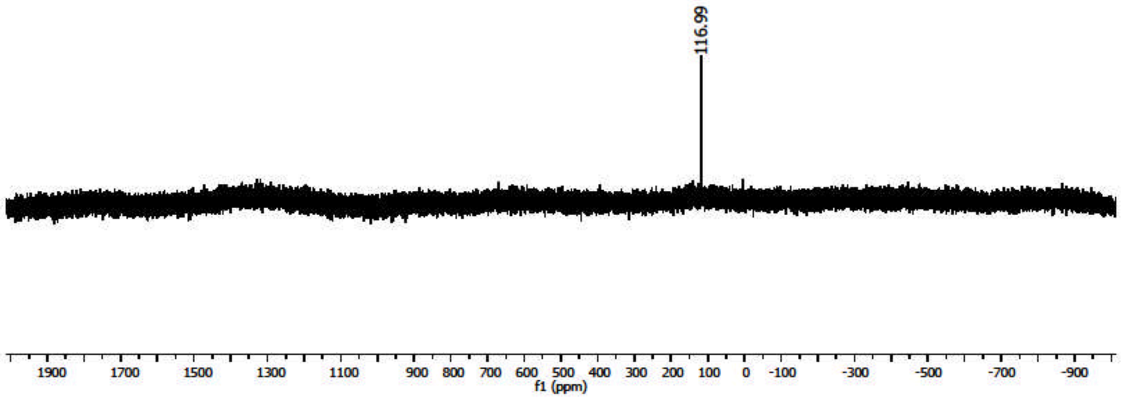

[Au(IPr)F]: ^{1}H NMR

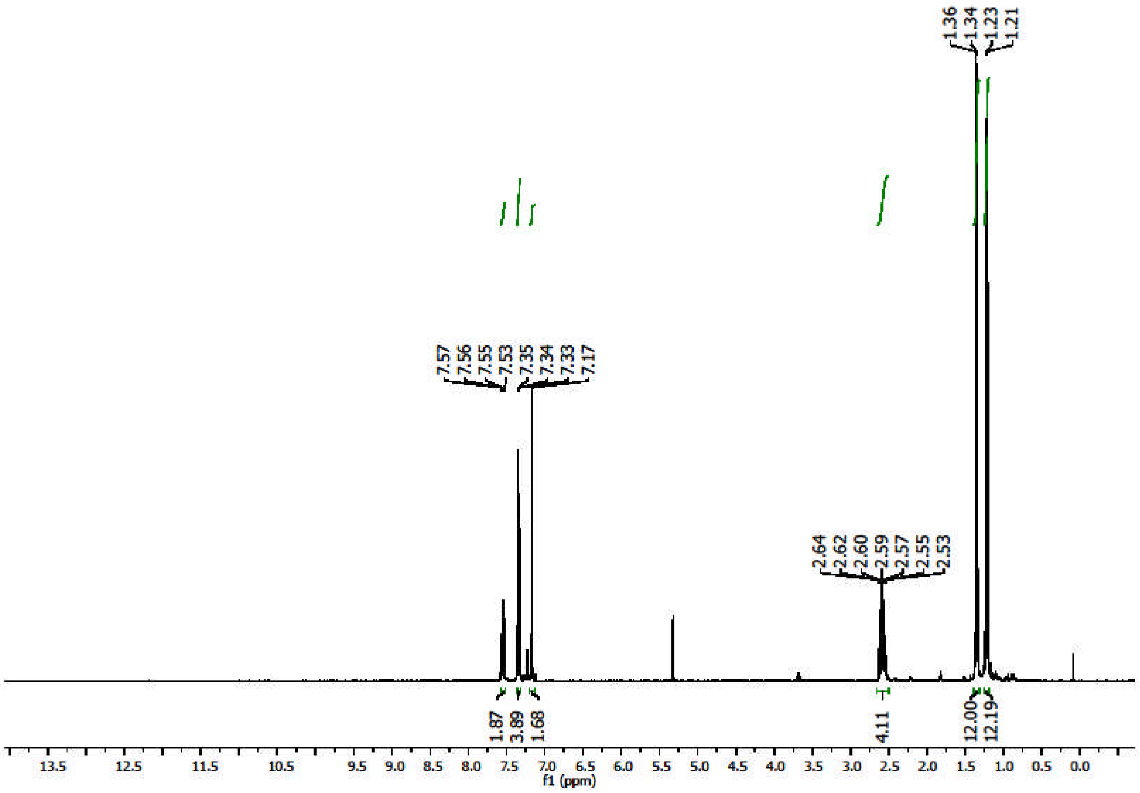

[Au(IPr)F]: ^{13}C{^{1}H} NMR

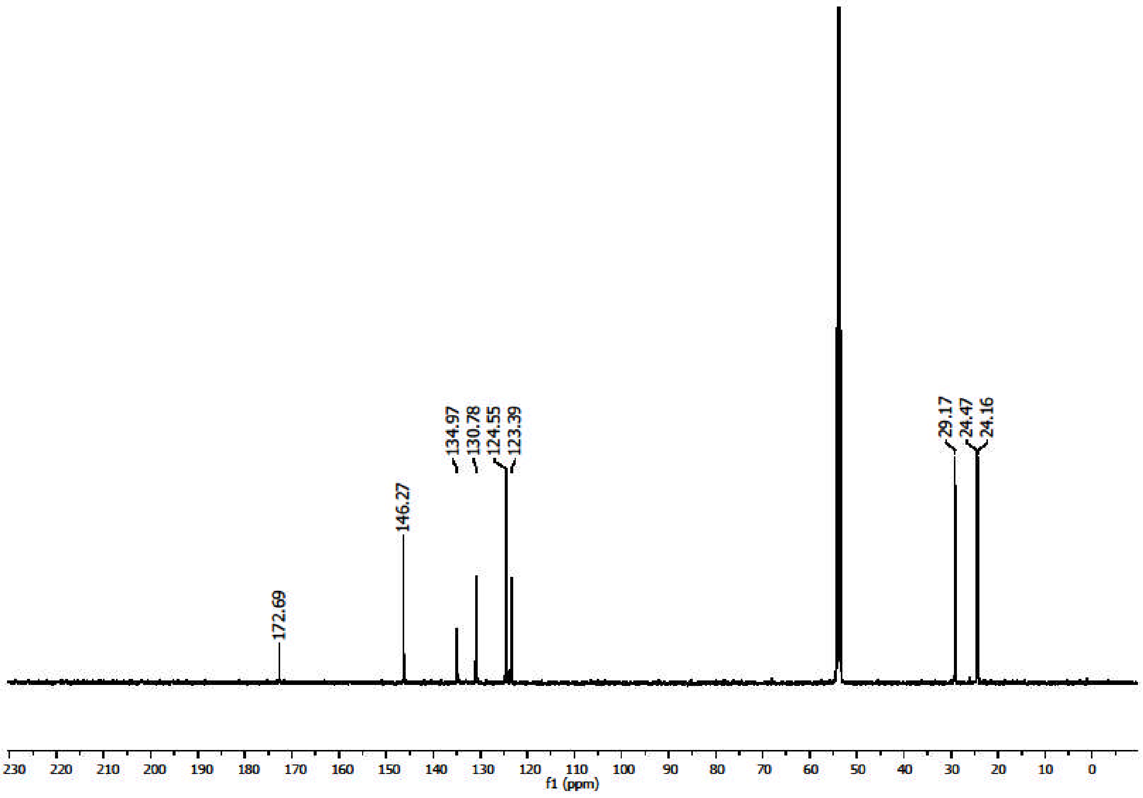

[Au(IPr)F]: ^{19}F NMR

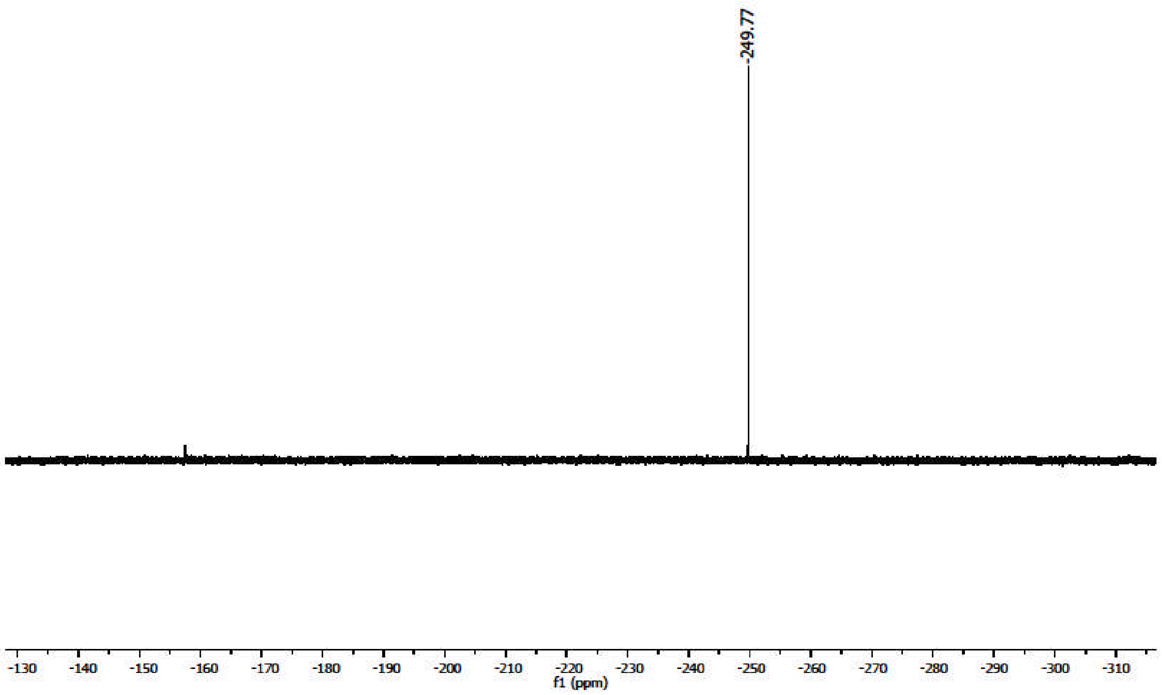

8a: ^{1}H NMR

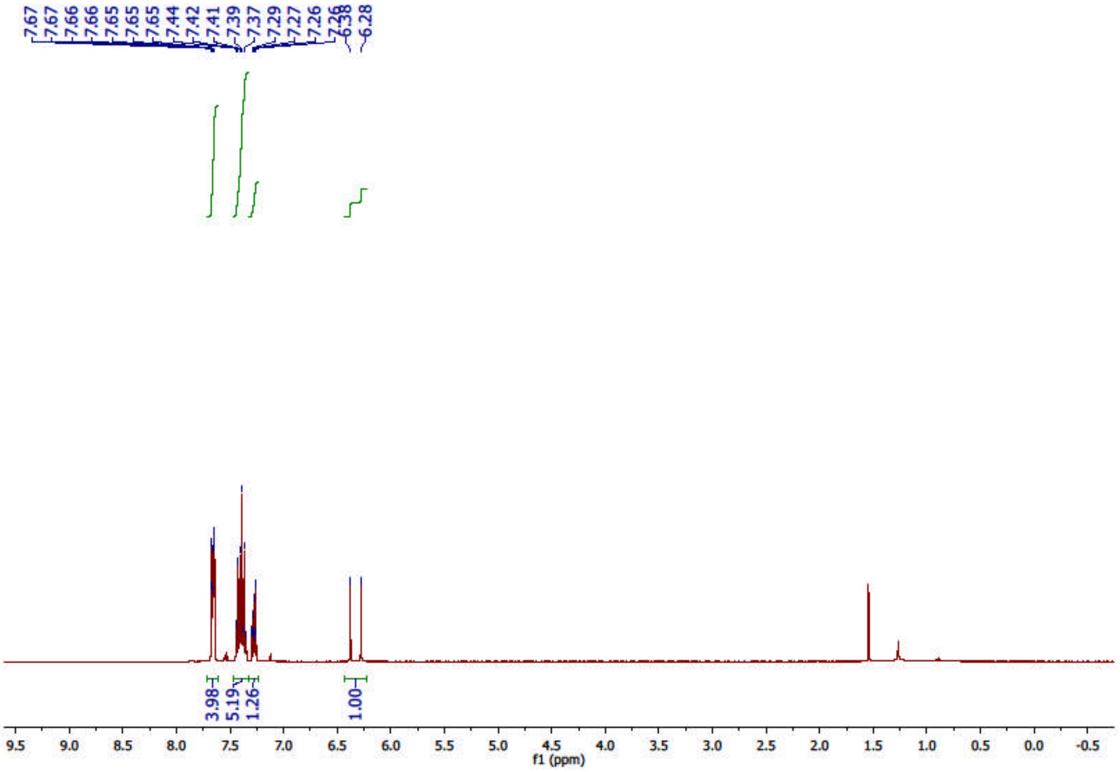

8a: ^{13}C NMR

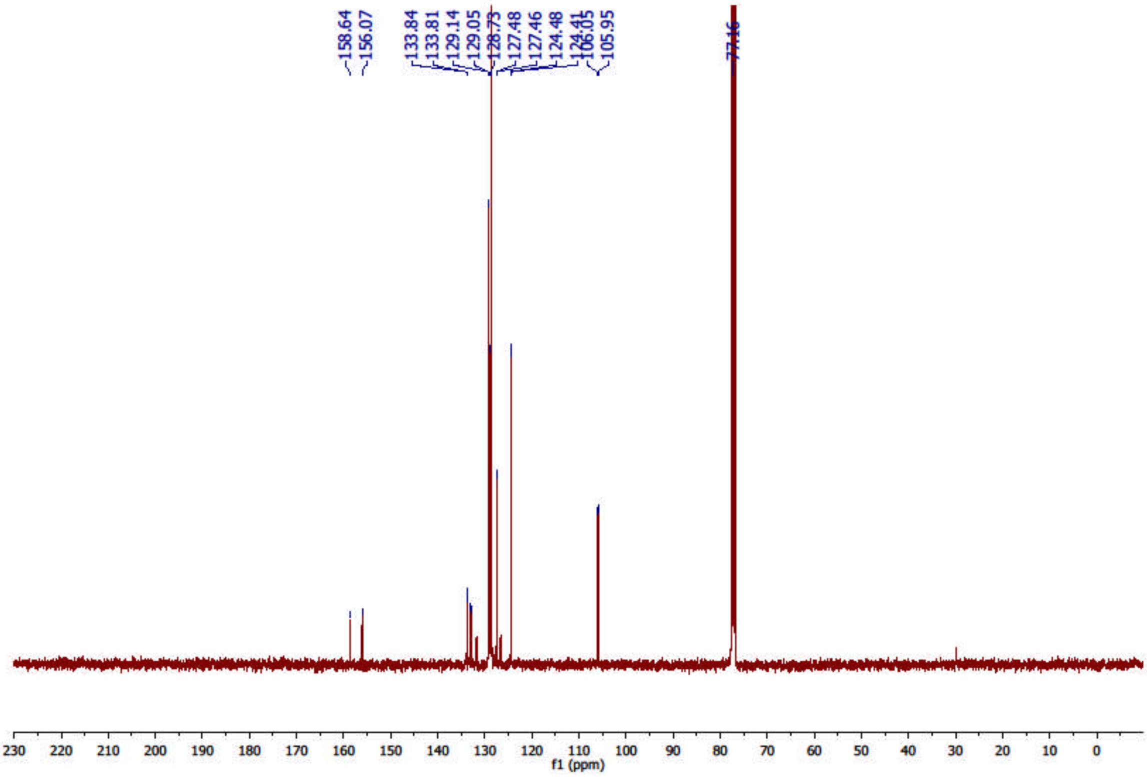

8a: ^{19}F NMR

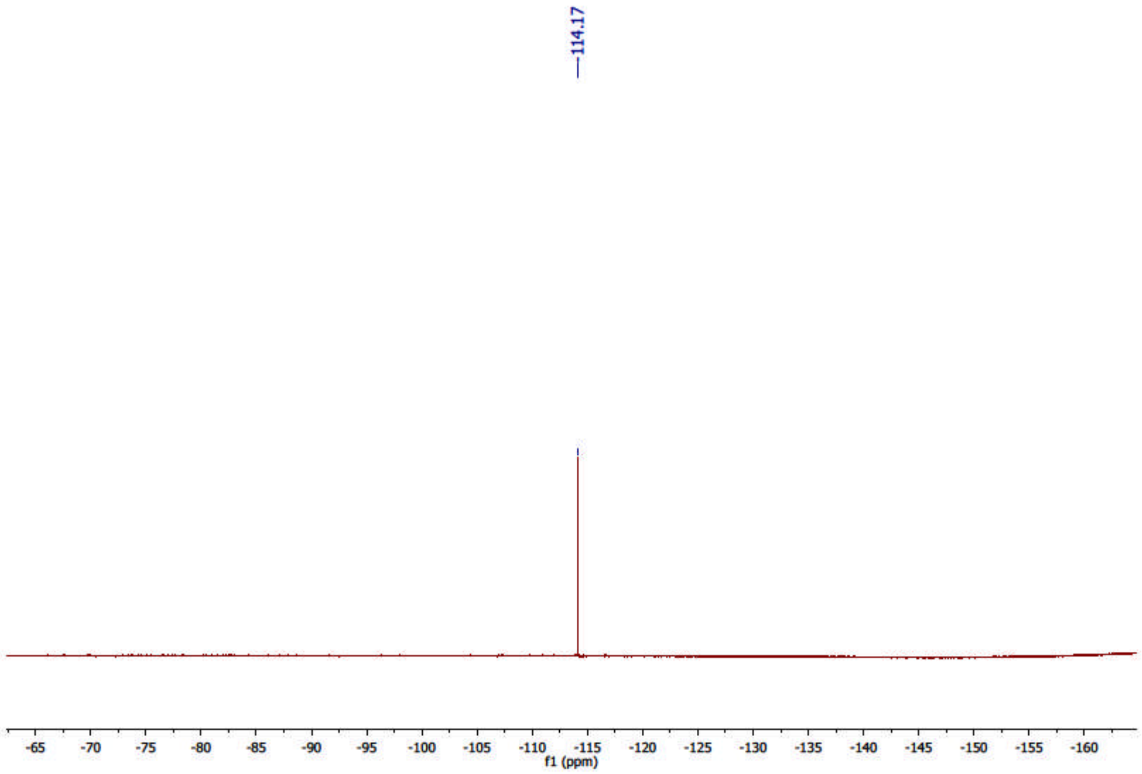

8b: ^{1}H NMR

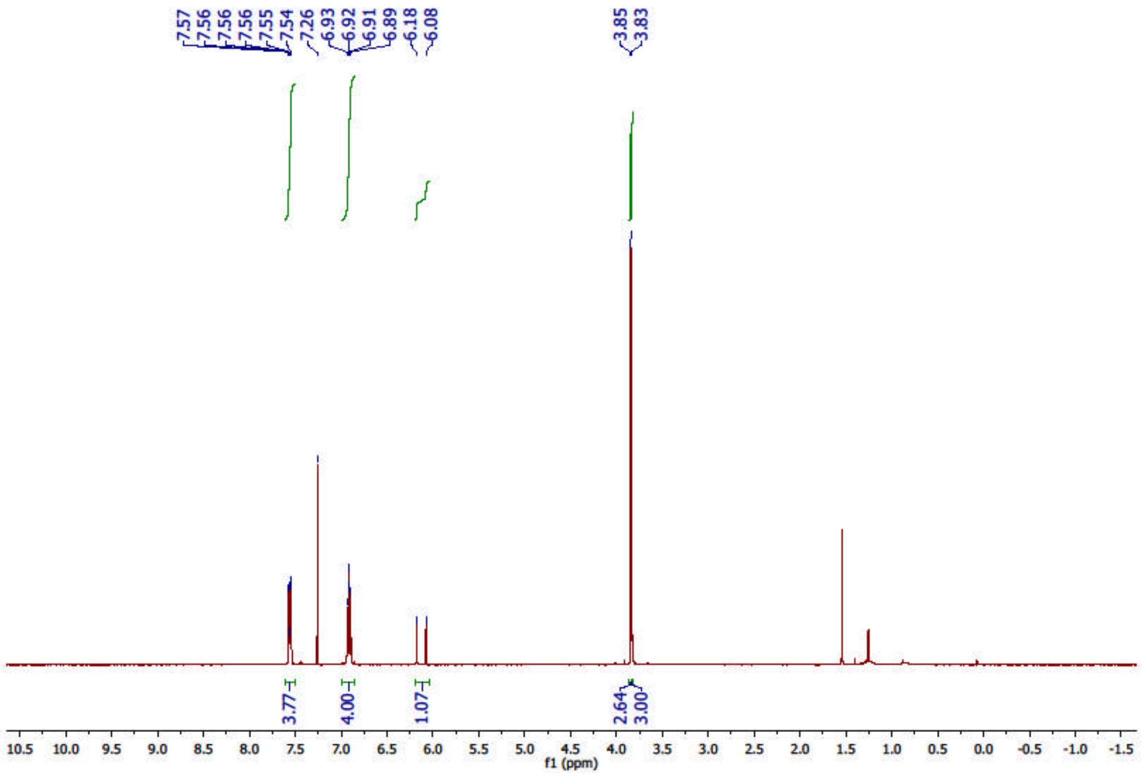

8b: ^{13}C NMR

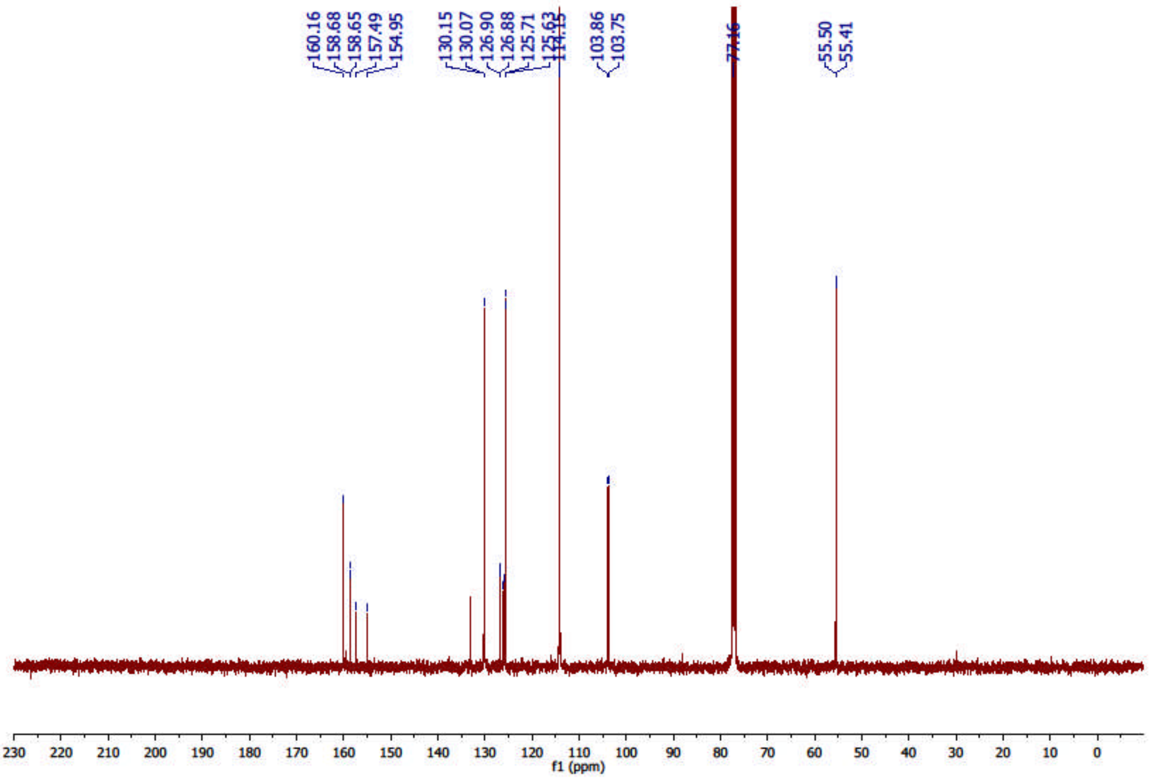

8b: ^{19}F NMR

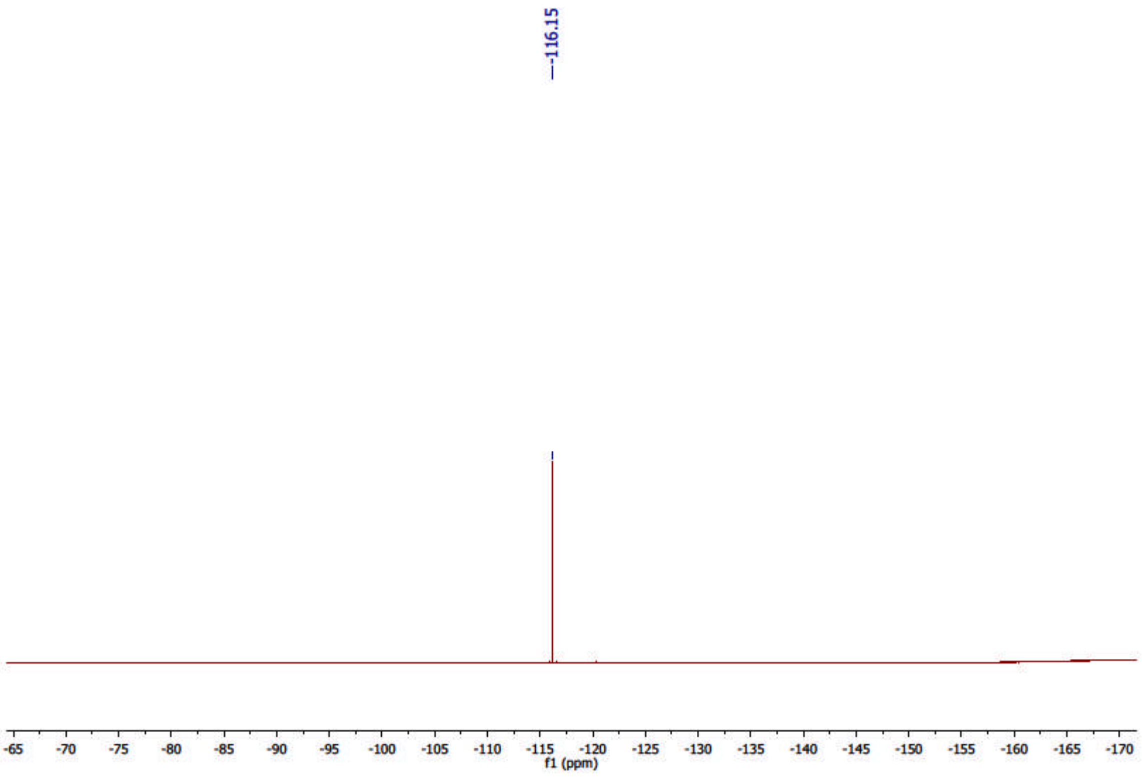

8c: ^{1}H NMR

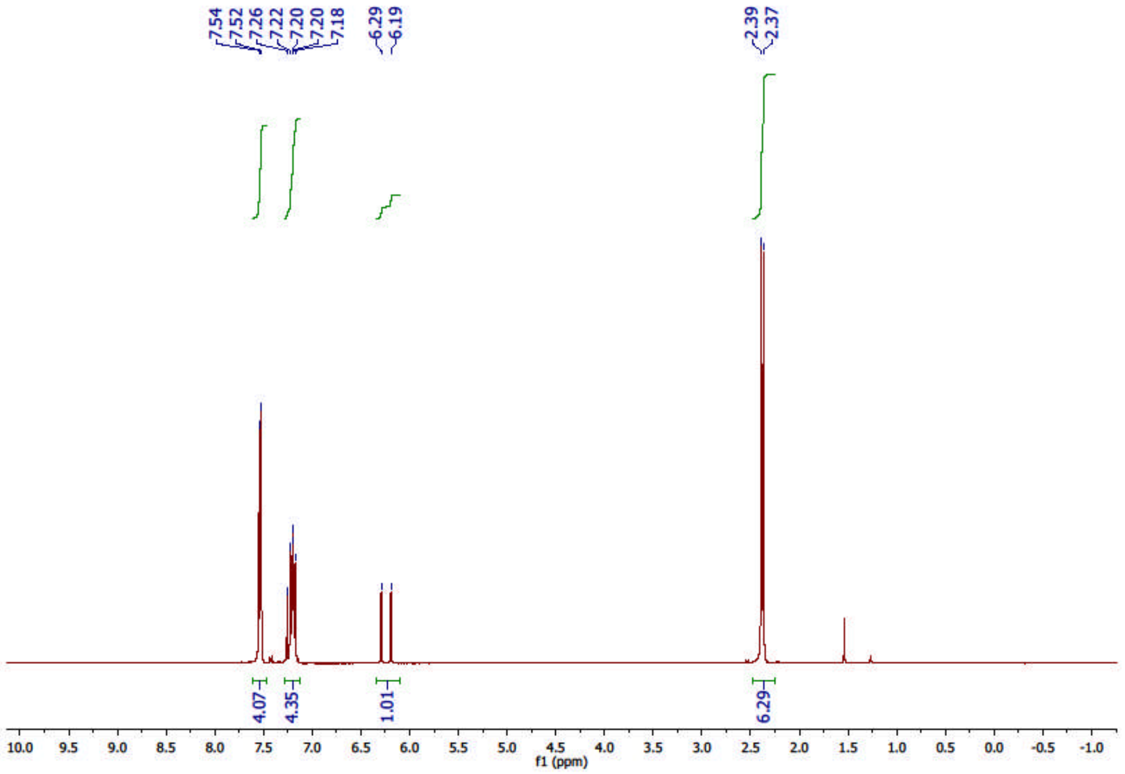

8c: ^{13}C NMR

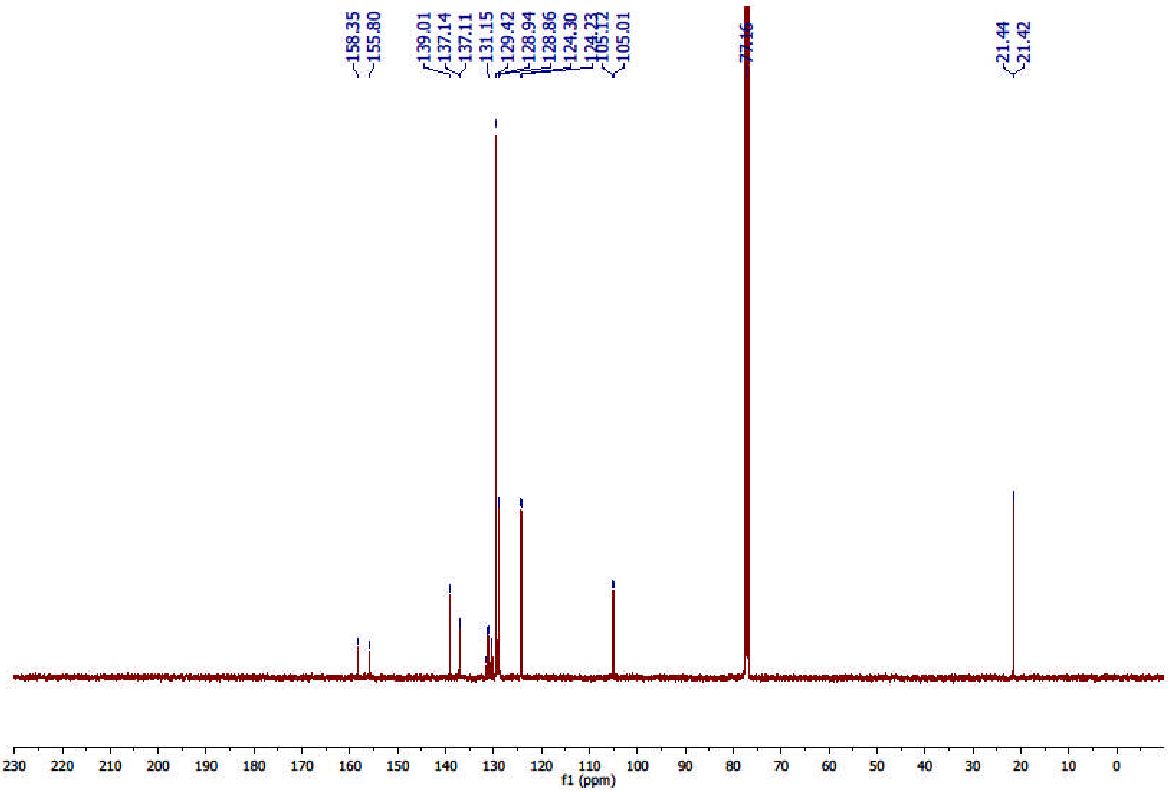

8c: ^{19}F NMR

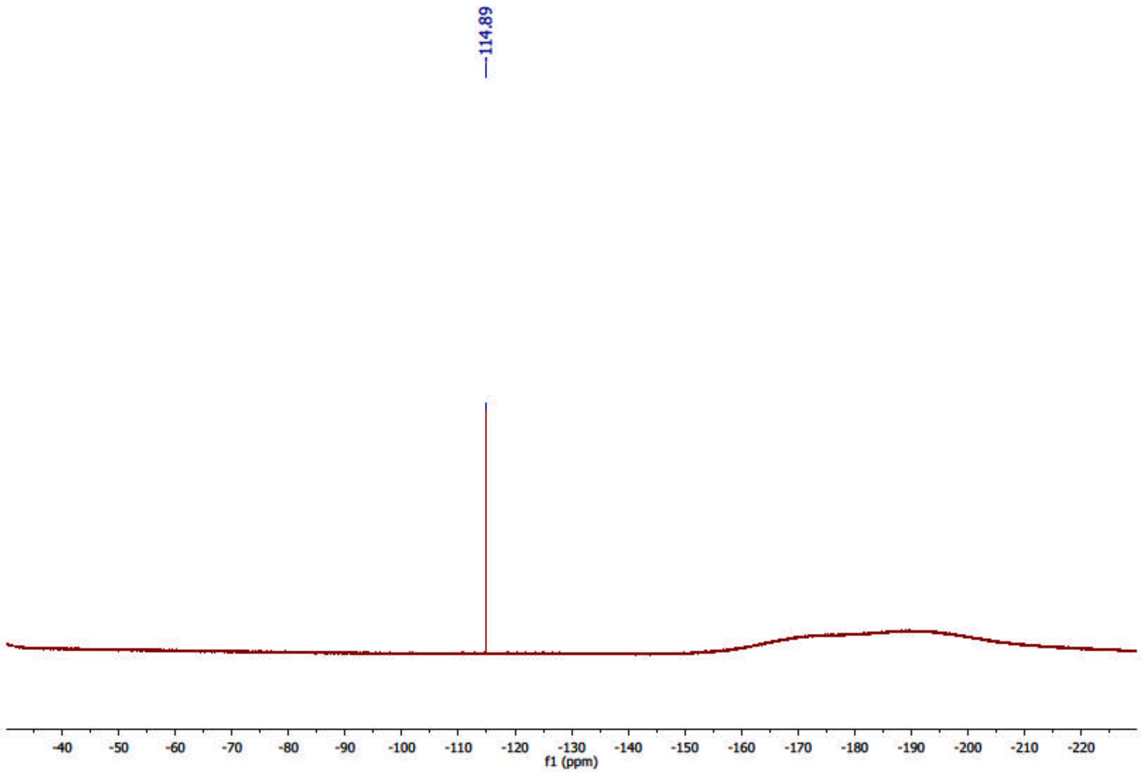

8d: ^{1}H NMR

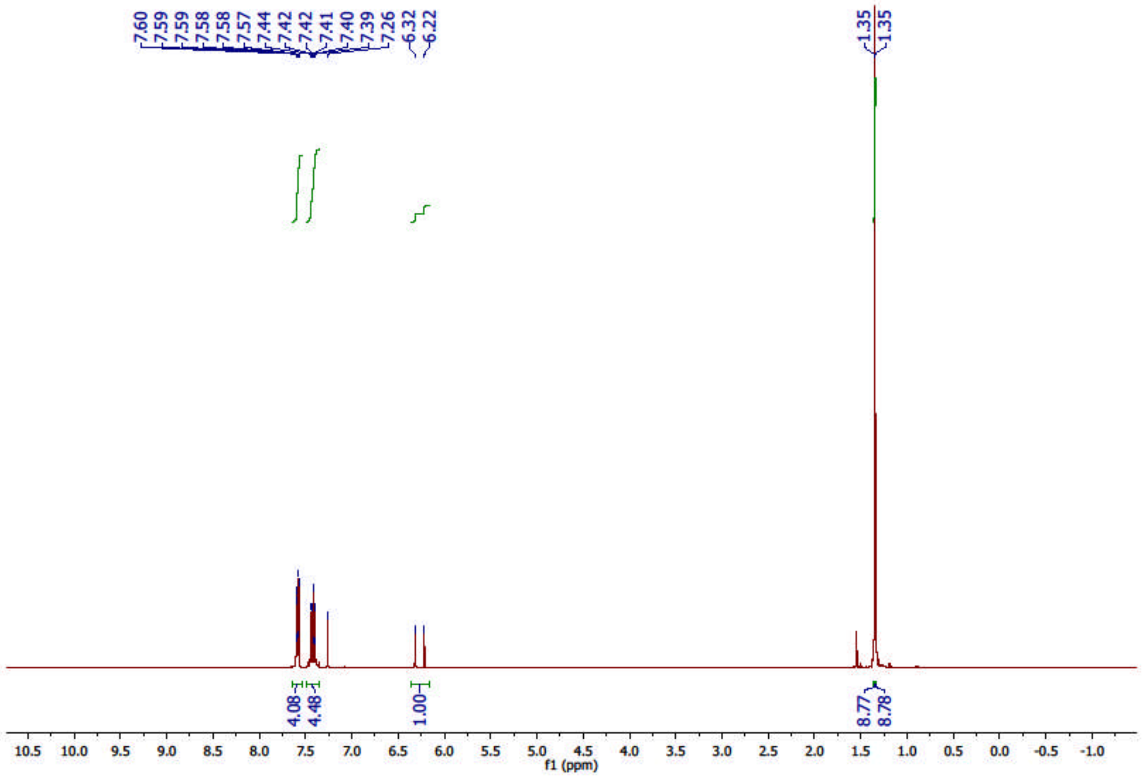

8d: ^{13}C NMR

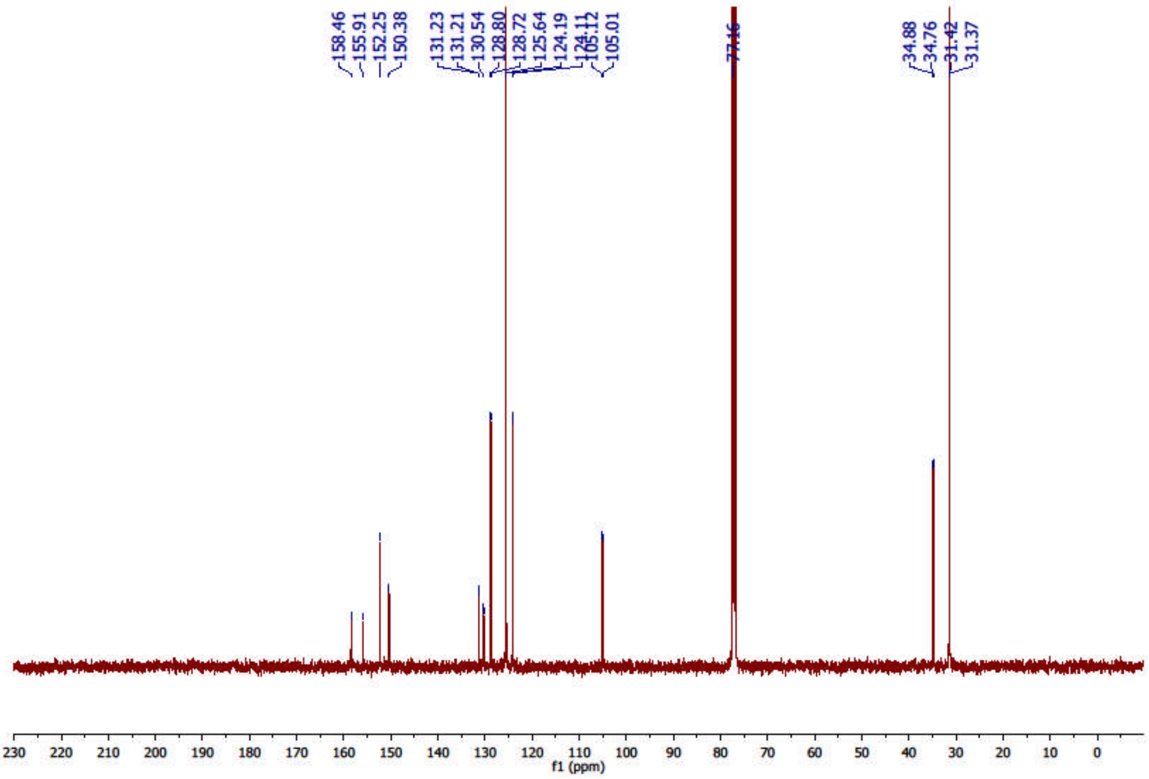

8d: ^{19}F NMR

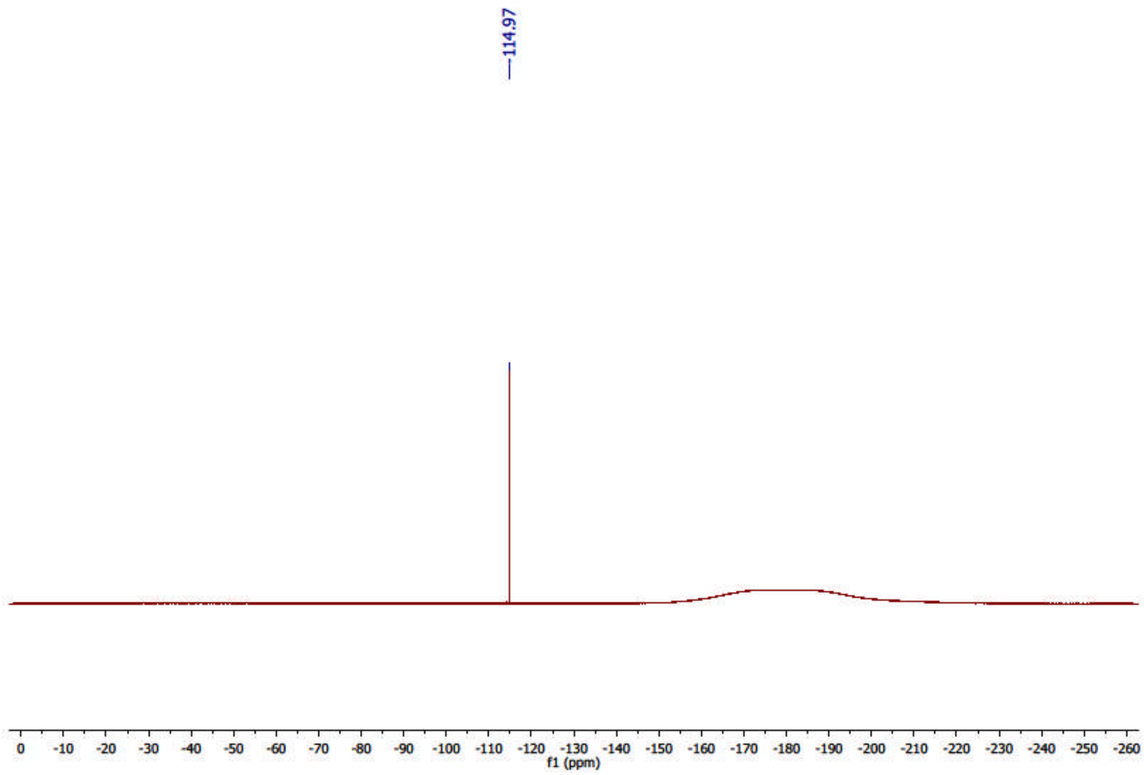

8e: ^{1}H NMR

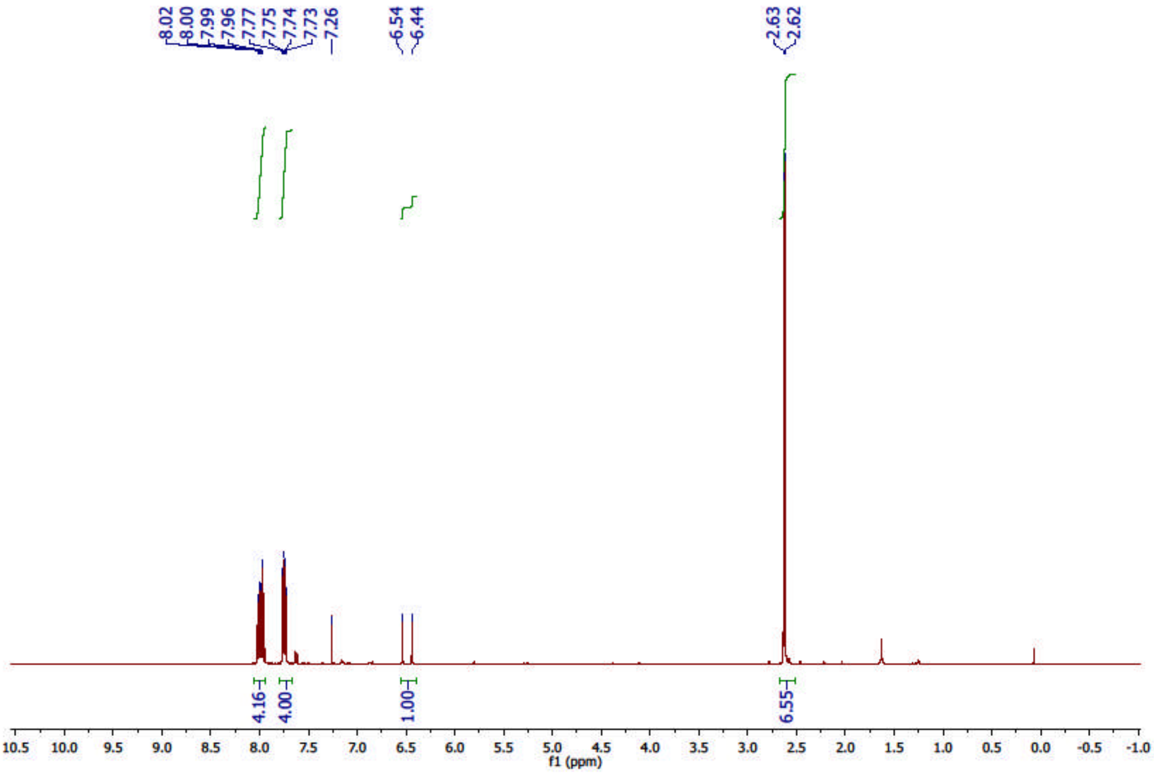

8e: ^{13}C NMR

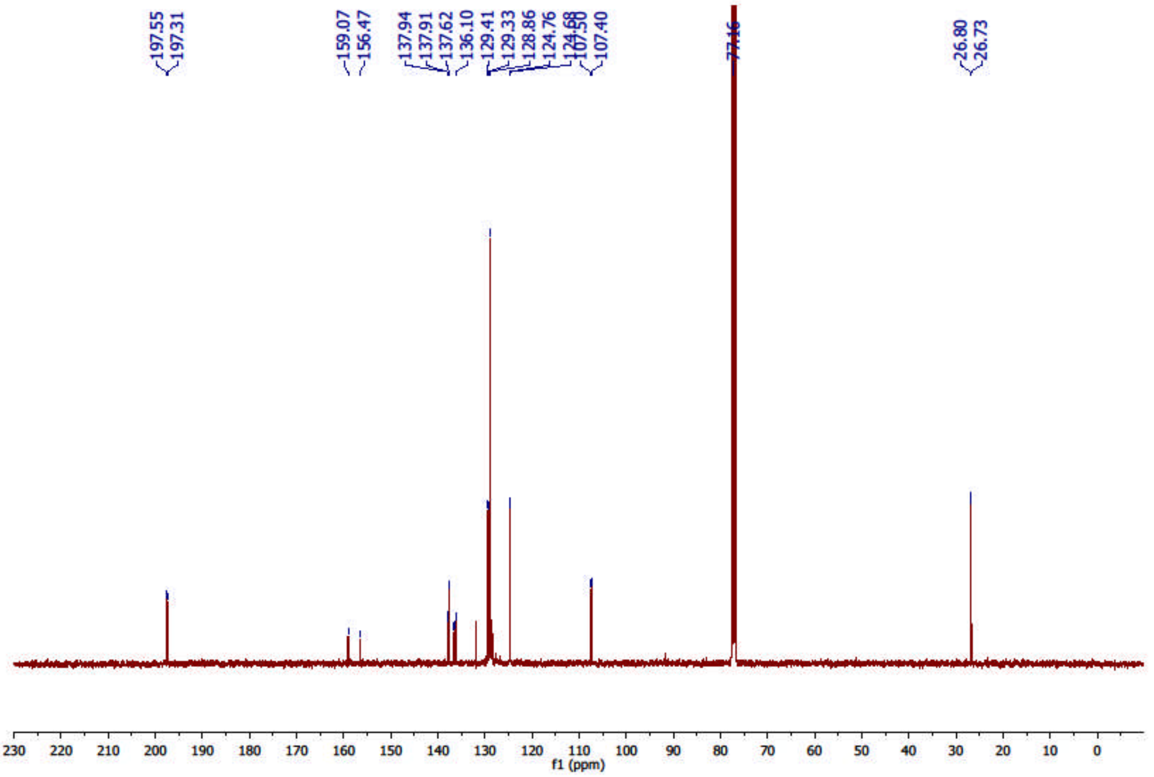

8e: ^{19}F NMR

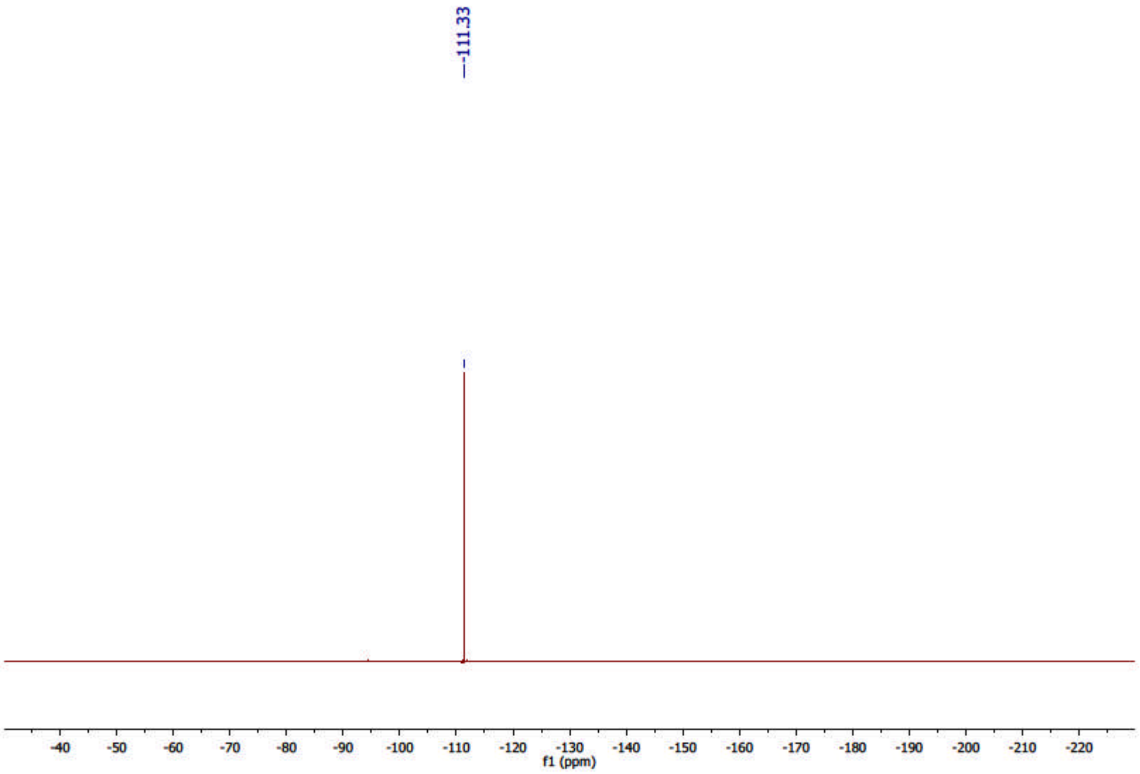

8f: ^{1}H NMR

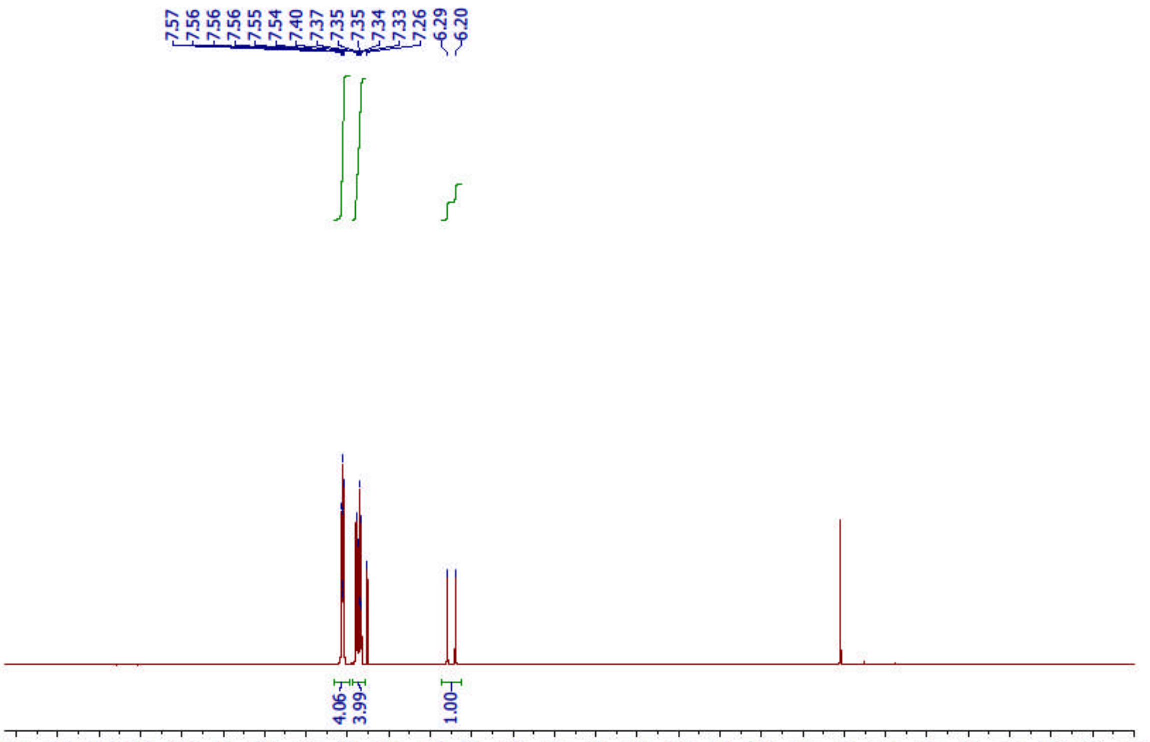

8f: ^{13}C NMR

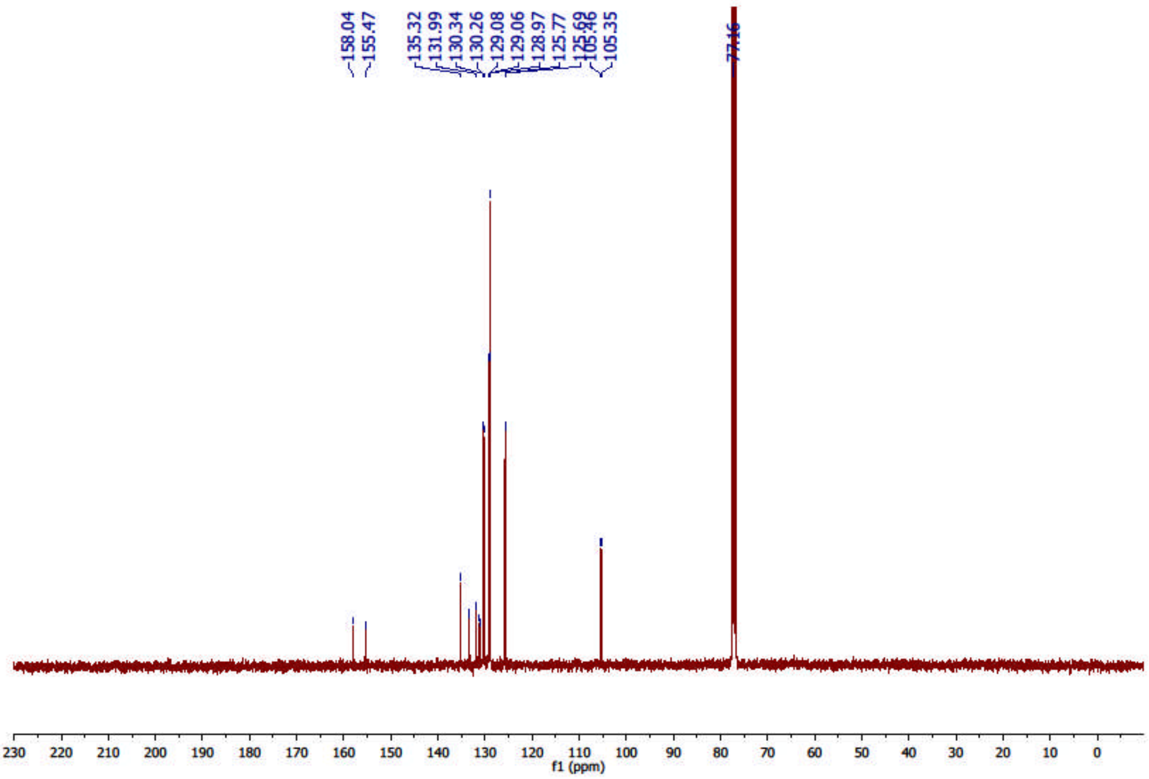

8f: ^{19}F NMR

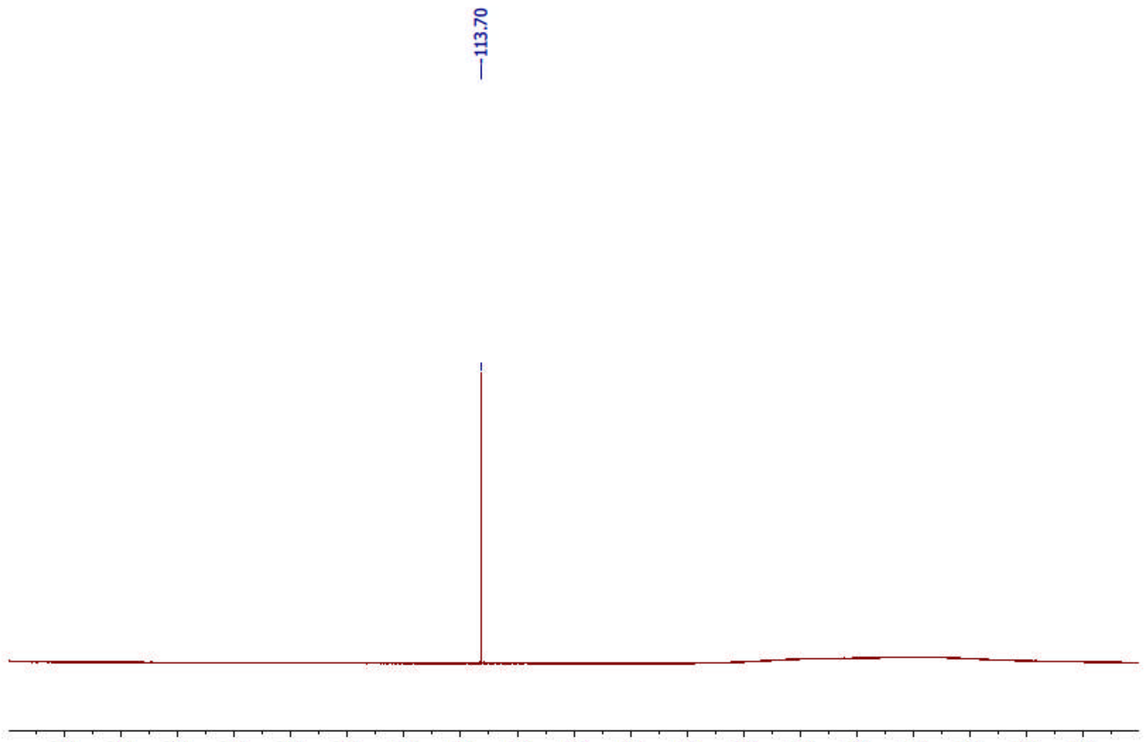

8g: ^{1}H NMR

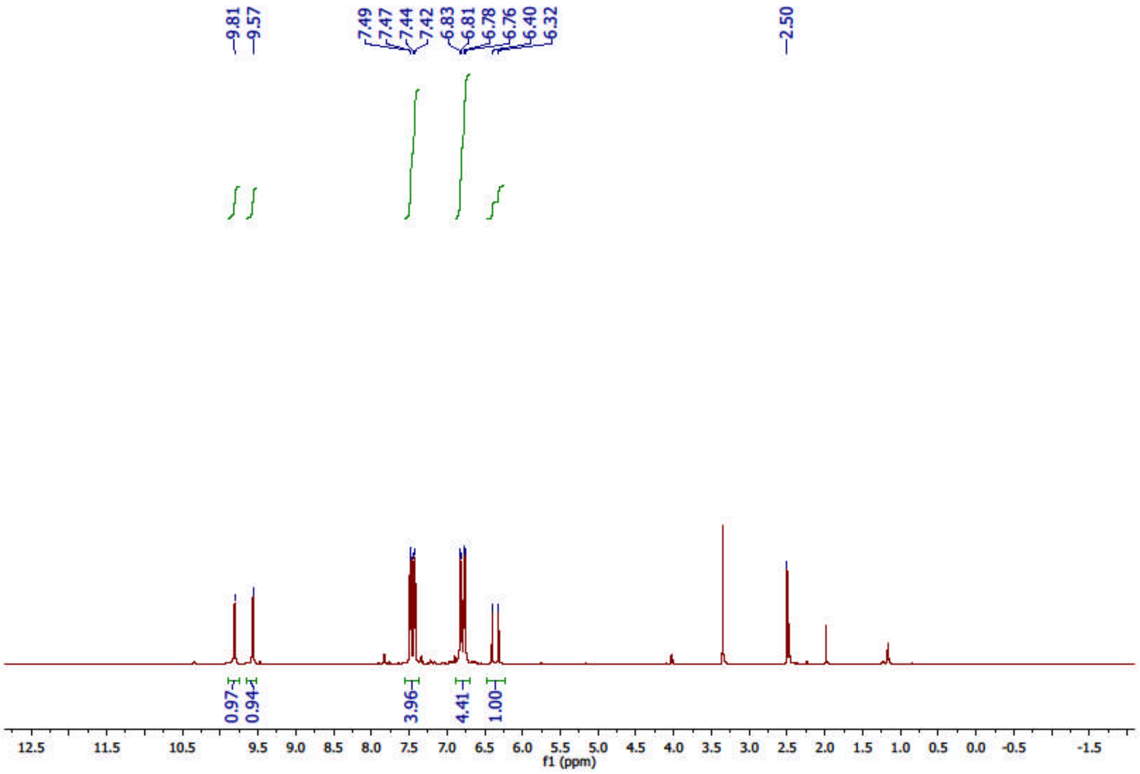

8g: ^{13}C NMR

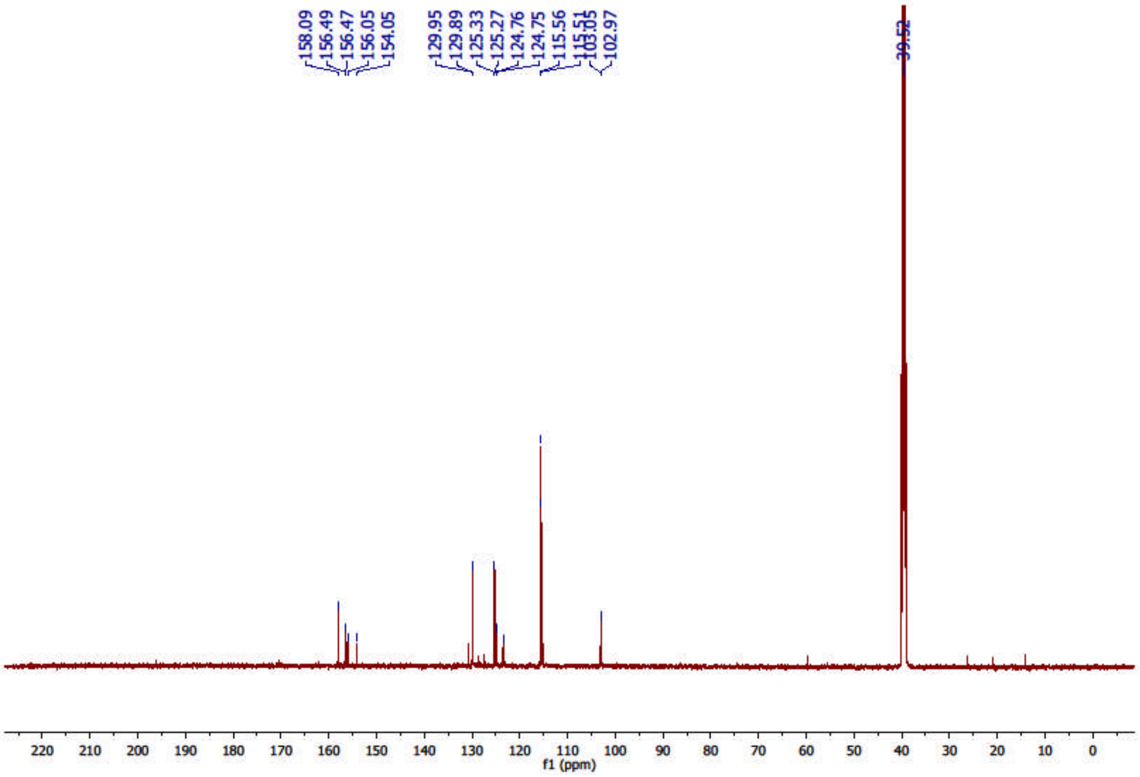

8g: ^{19}F NMR

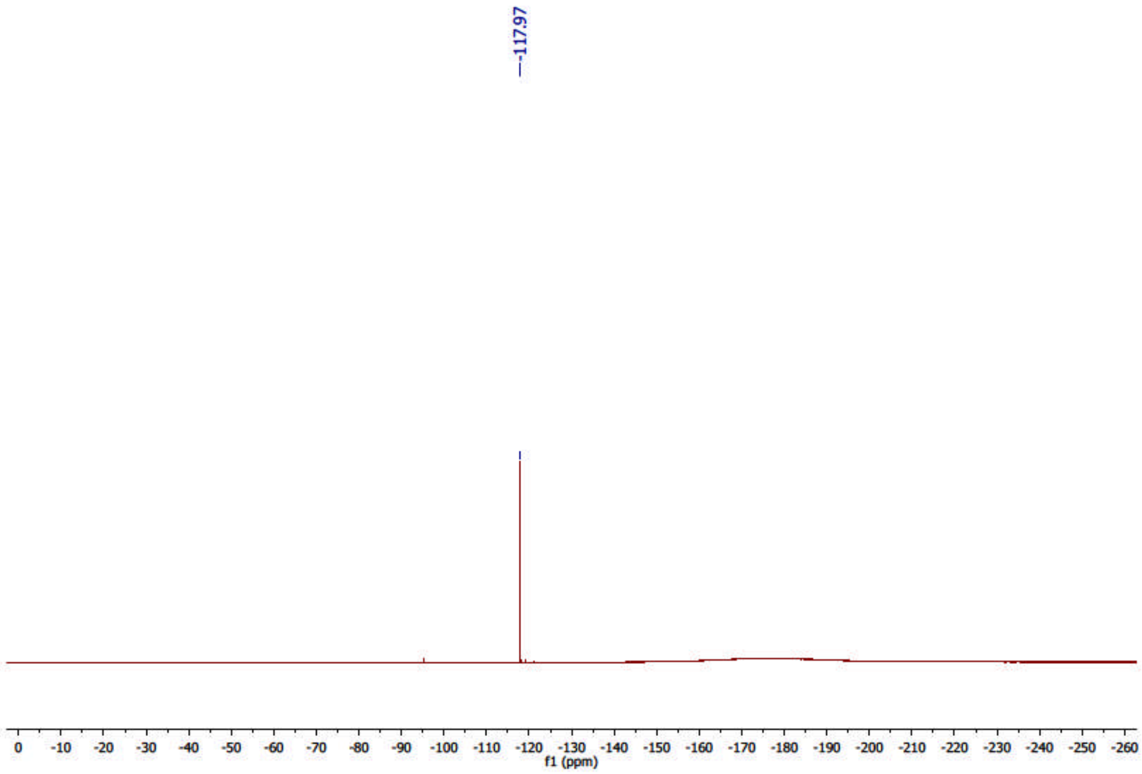

8h: ^{1}H NMR

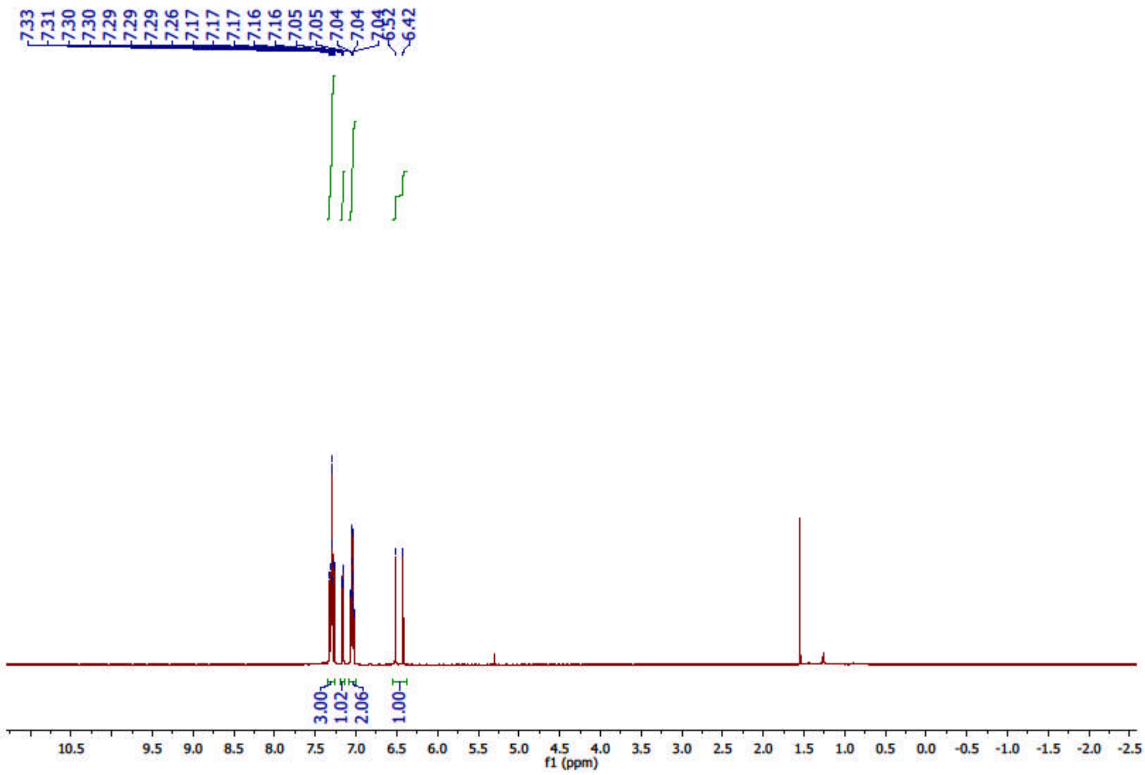

8h: ^{13}C NMR

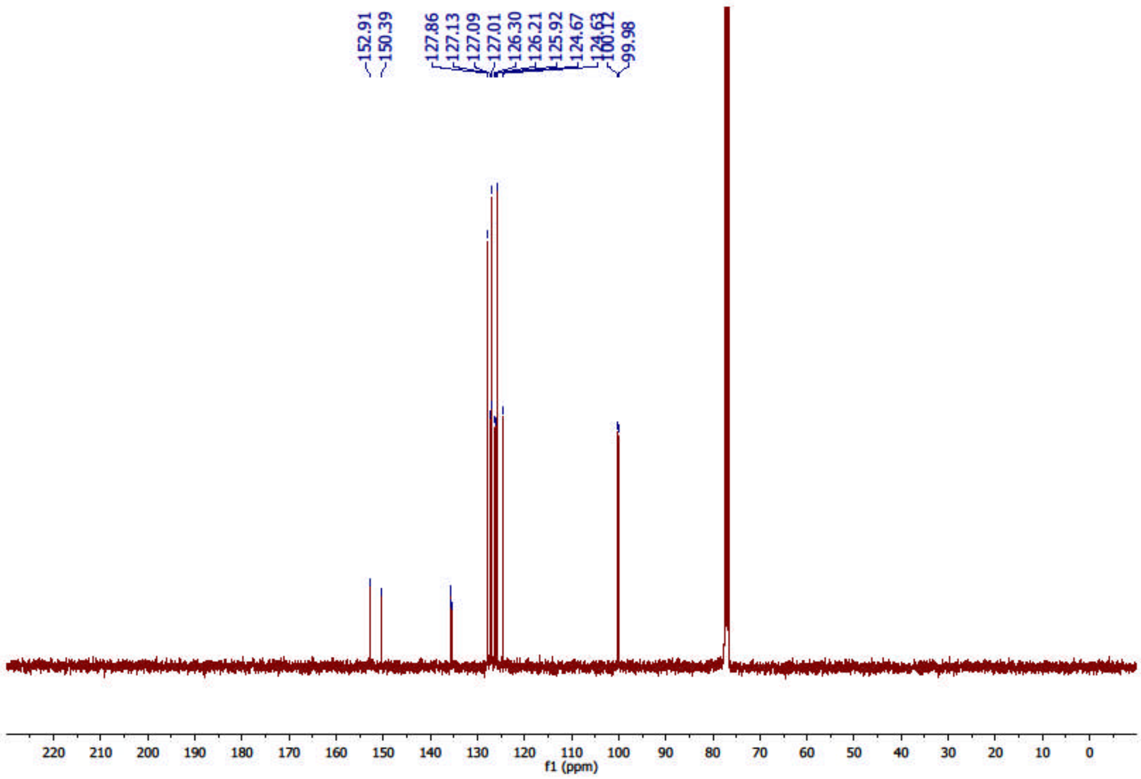

8h: ^{19}F NMR

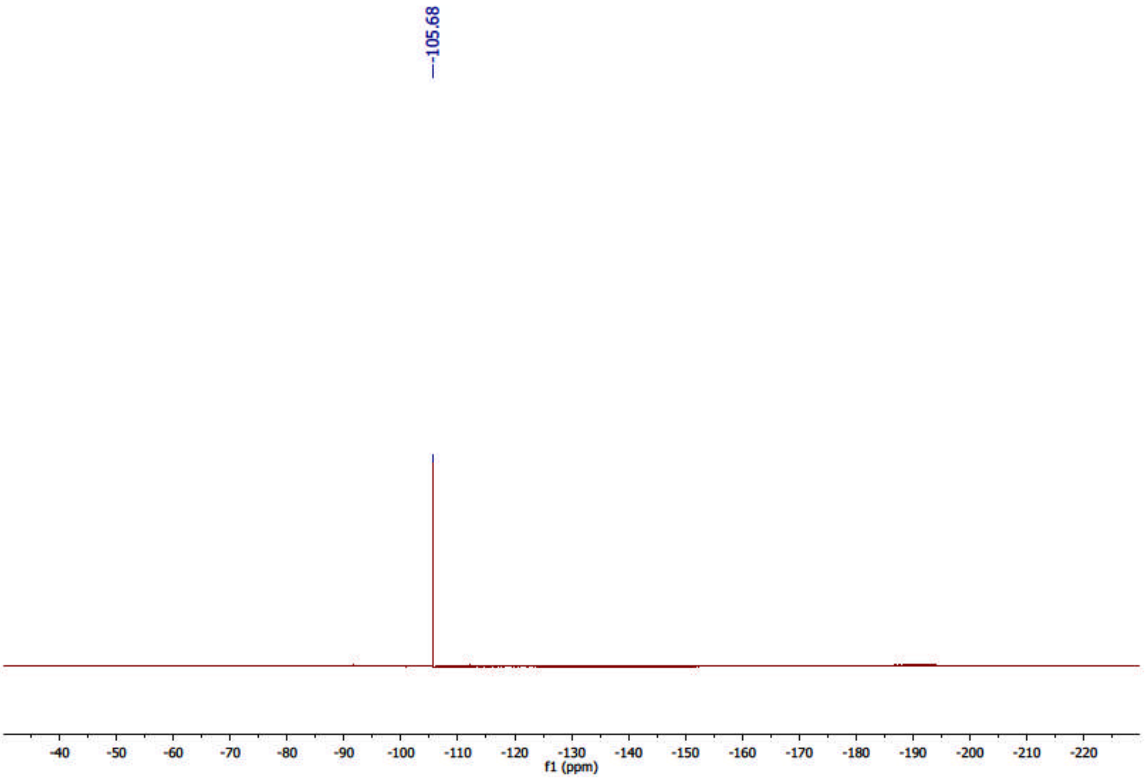

8i: ^{1}H NMR

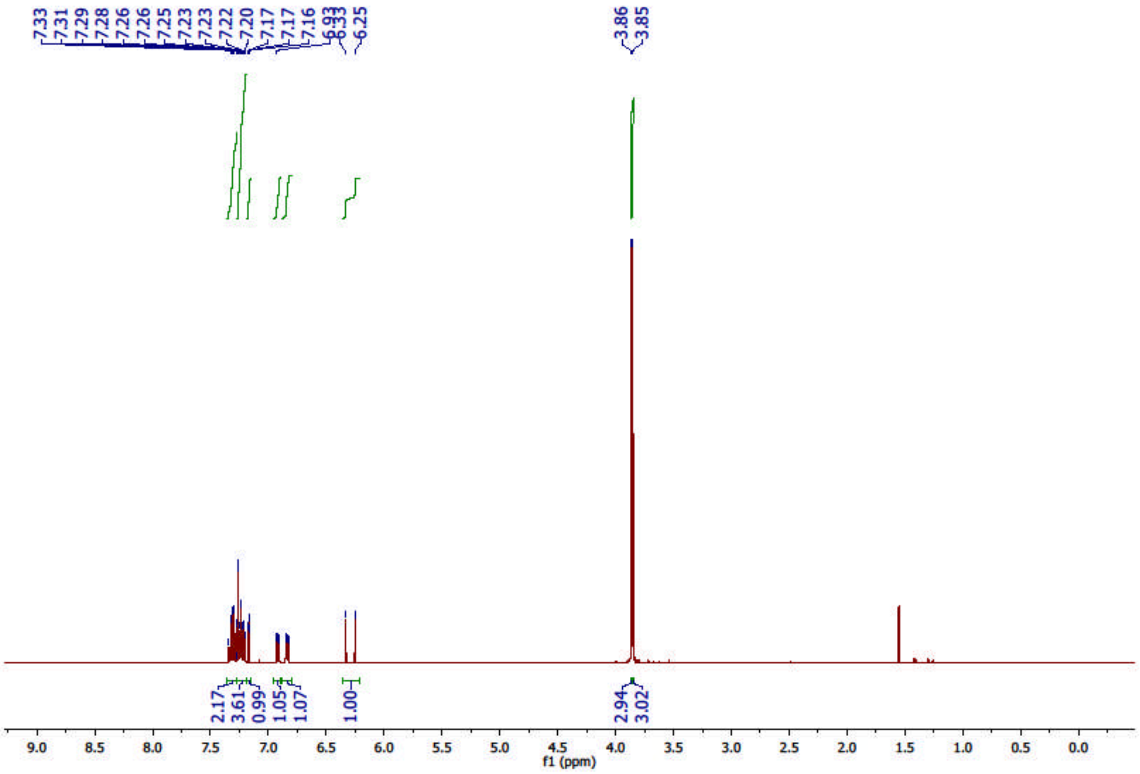

8i: ^{13}C NMR

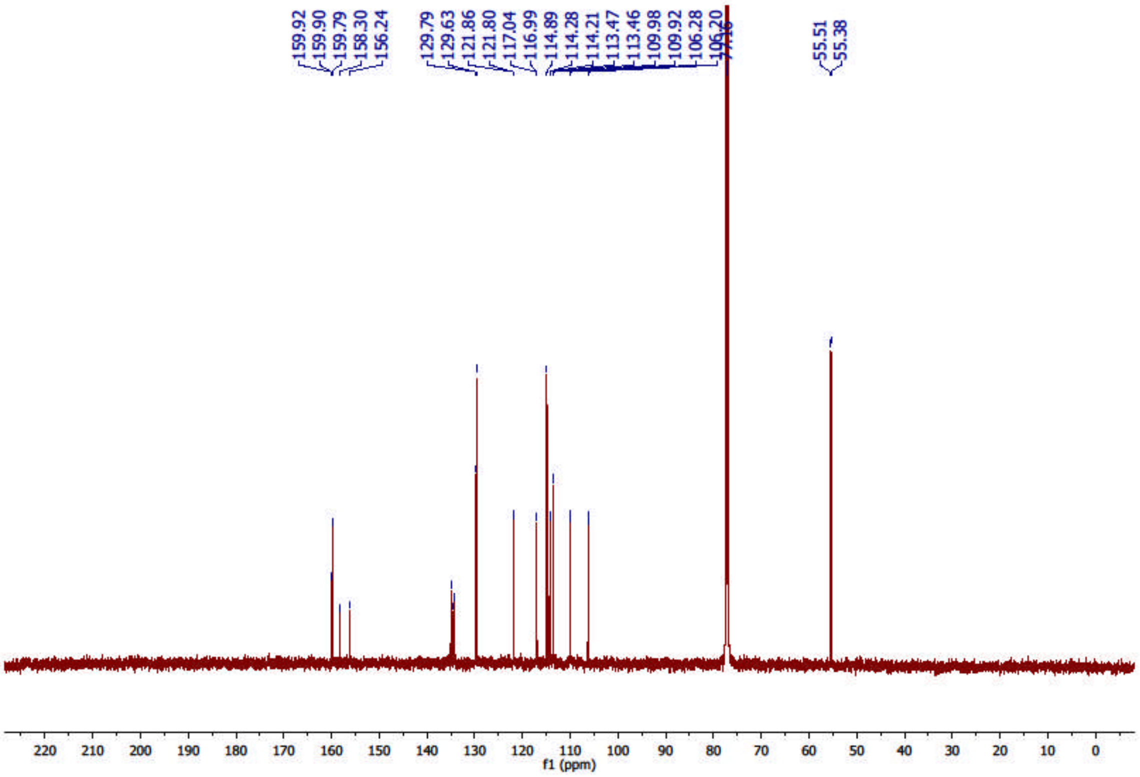

8i: ^{19}F NMR

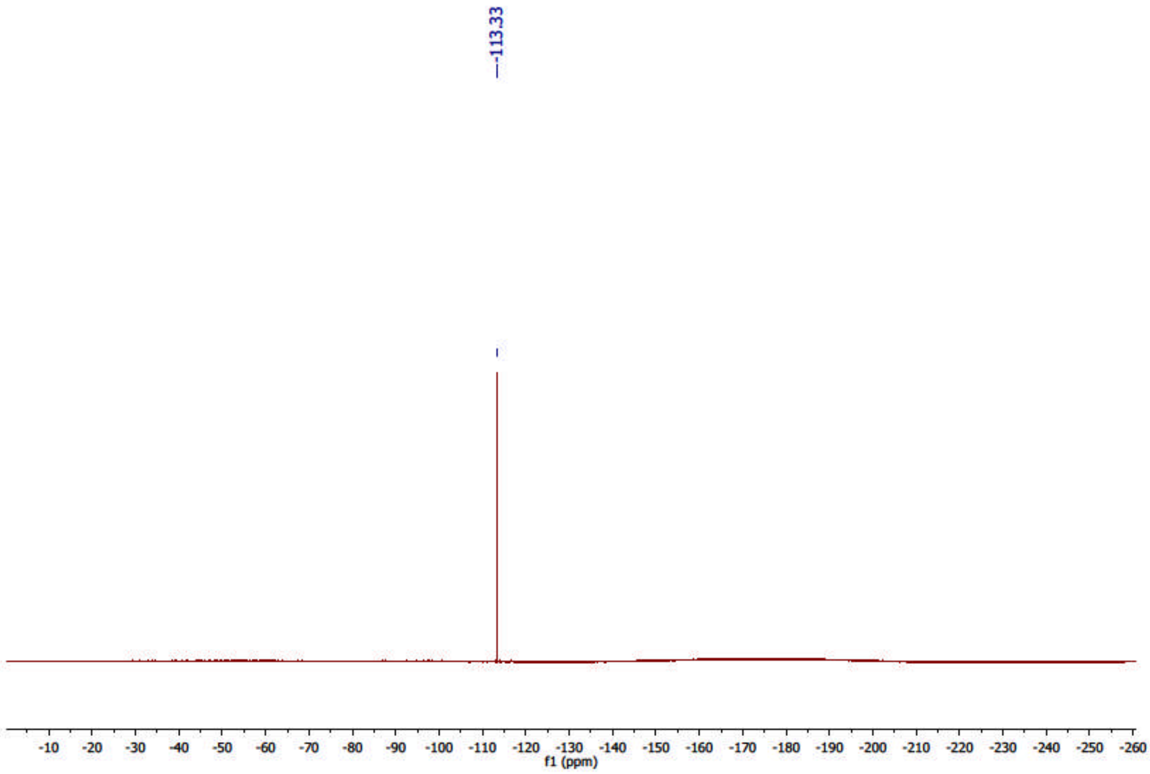

9g: ^{1}H NMR

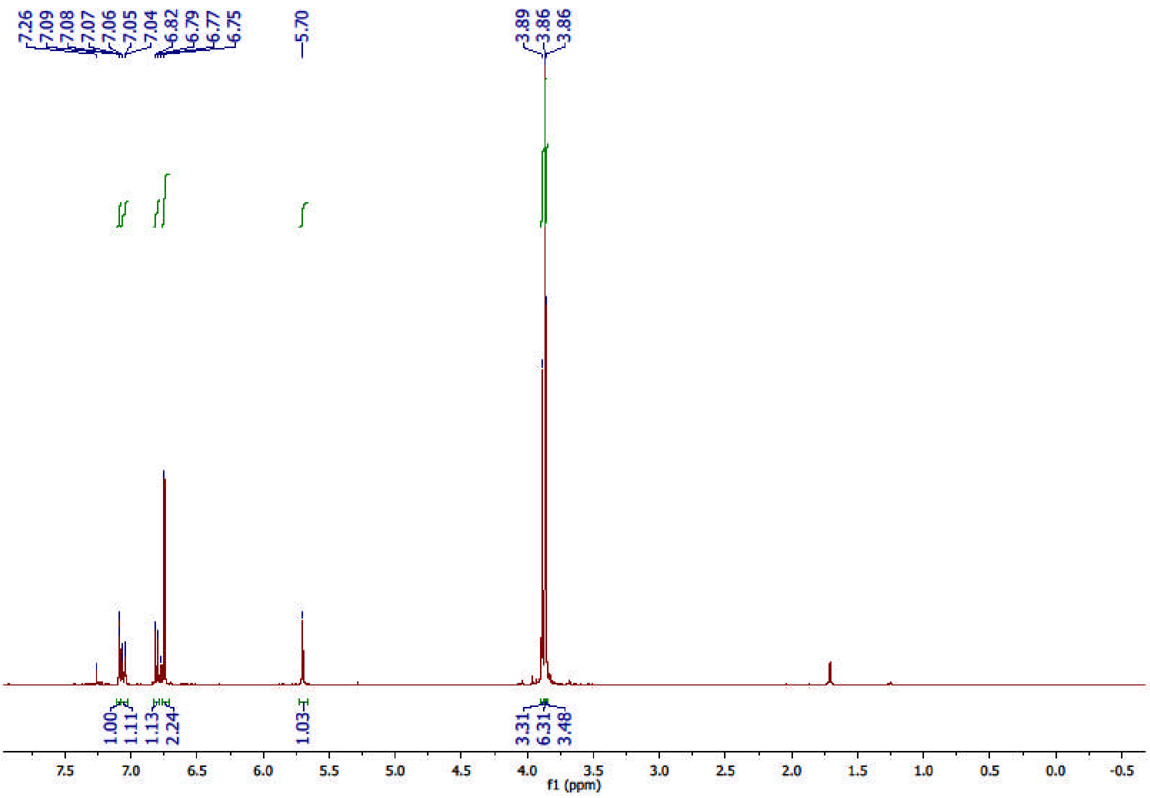

9g: ^{13}C NMR

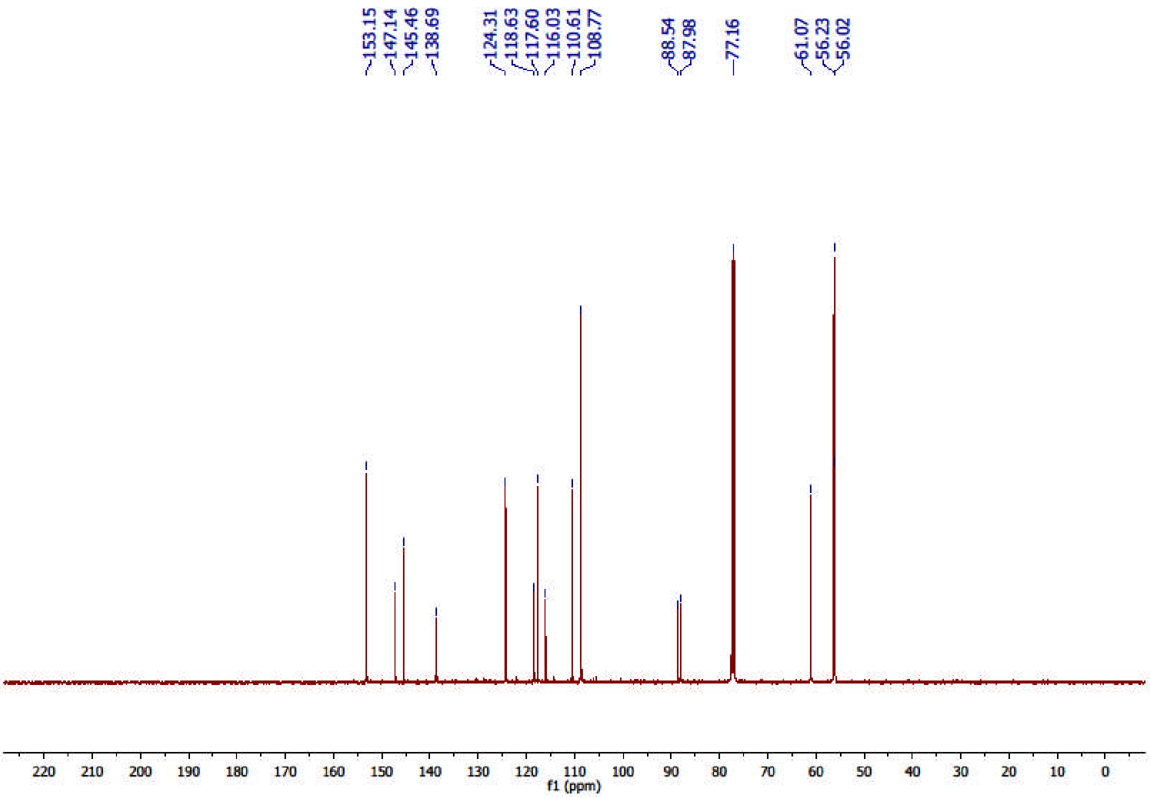

10a: ^{1}H NMR

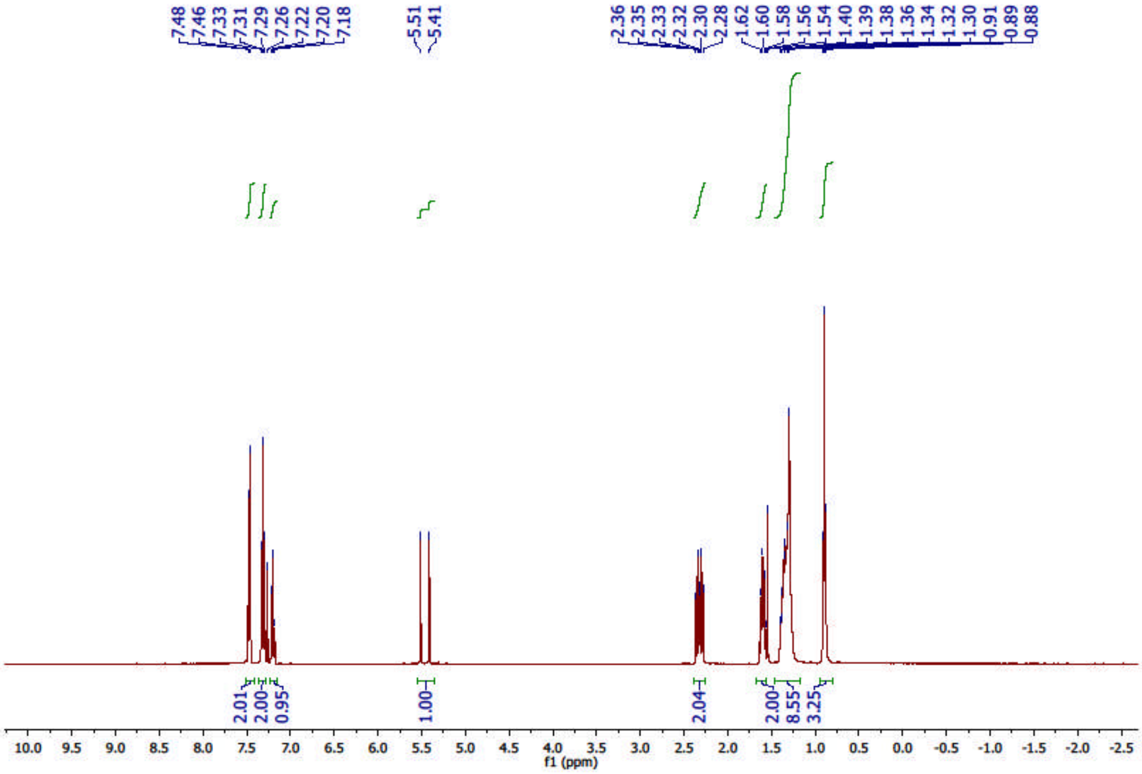

10a: ^{13}C NMR

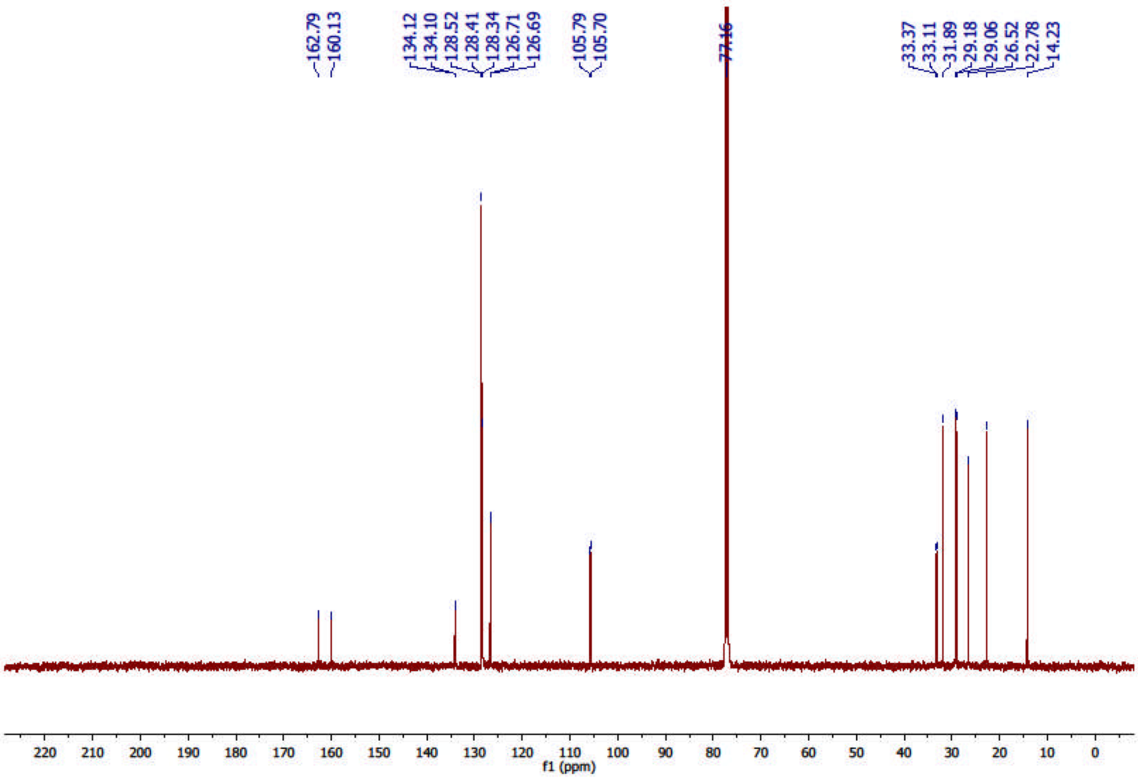

10a: ^{19}F NMR

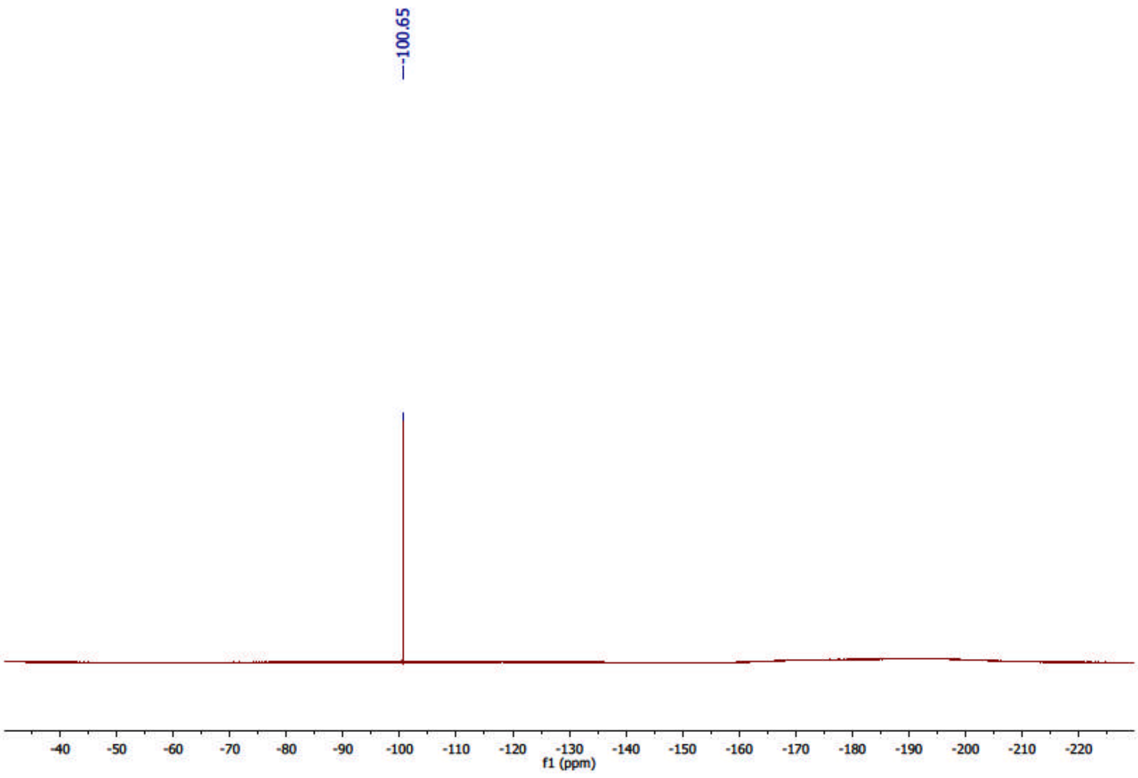

10b: ^{1}H NMR

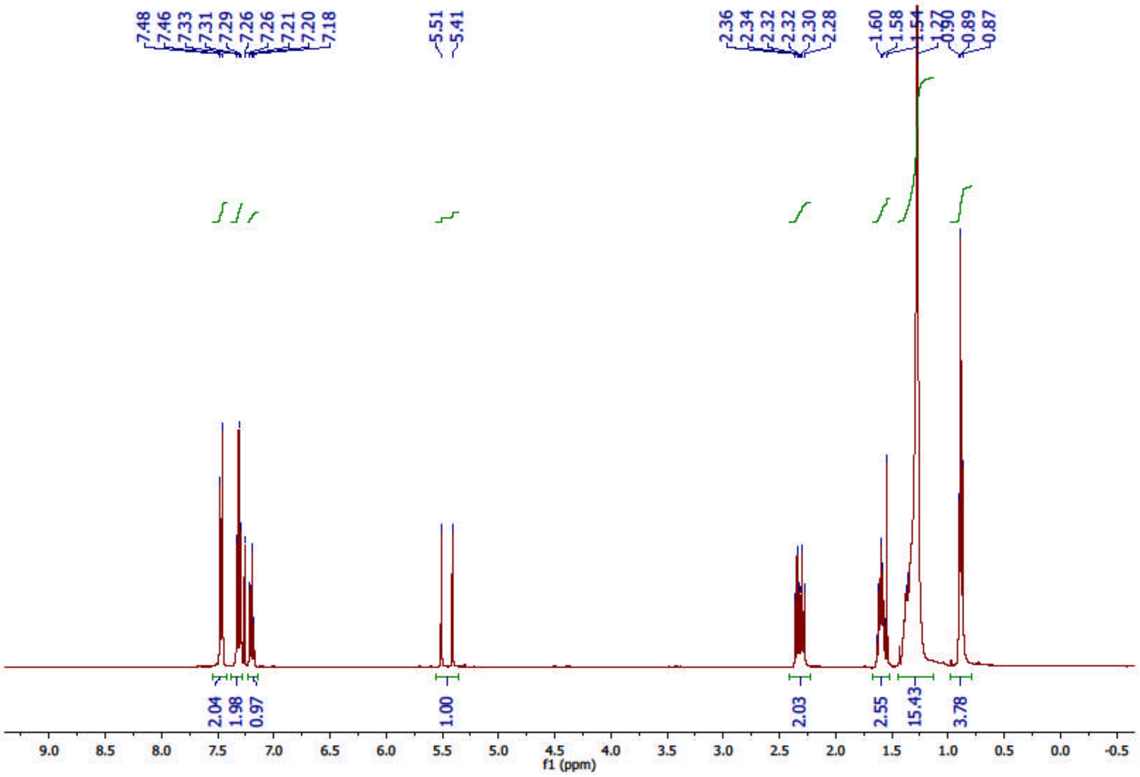

10b: ^{13}C NMR

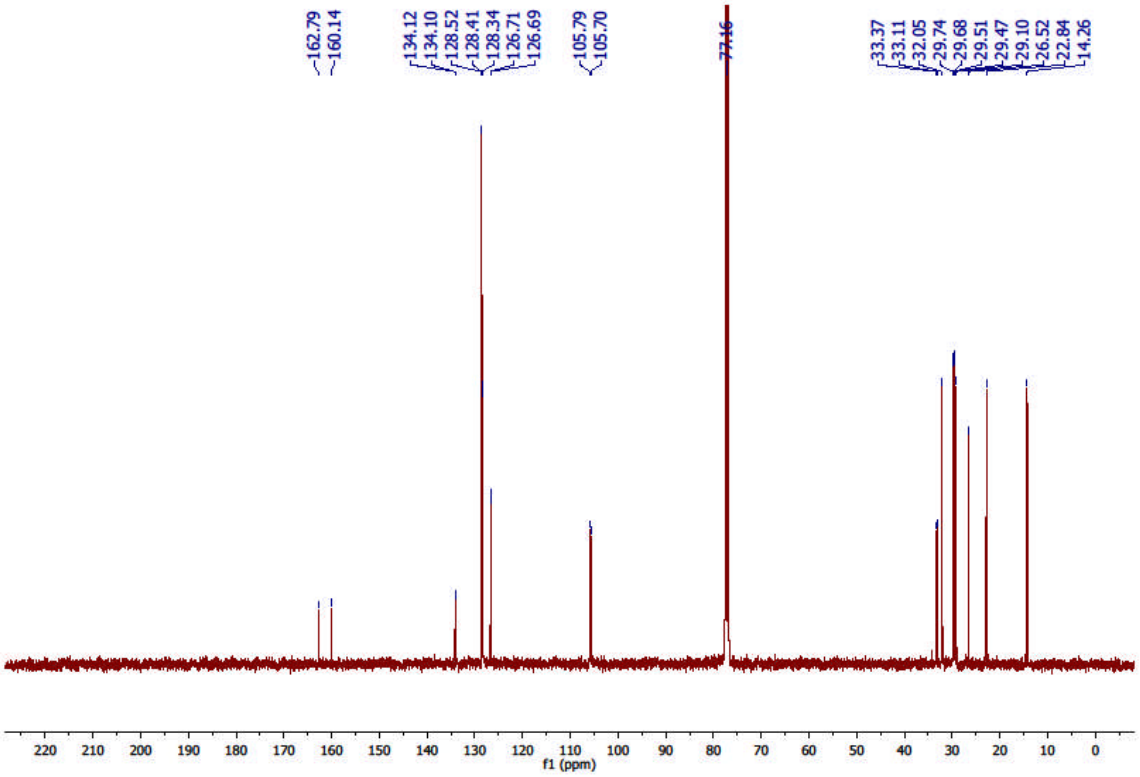

10b: ^{19}F NMR

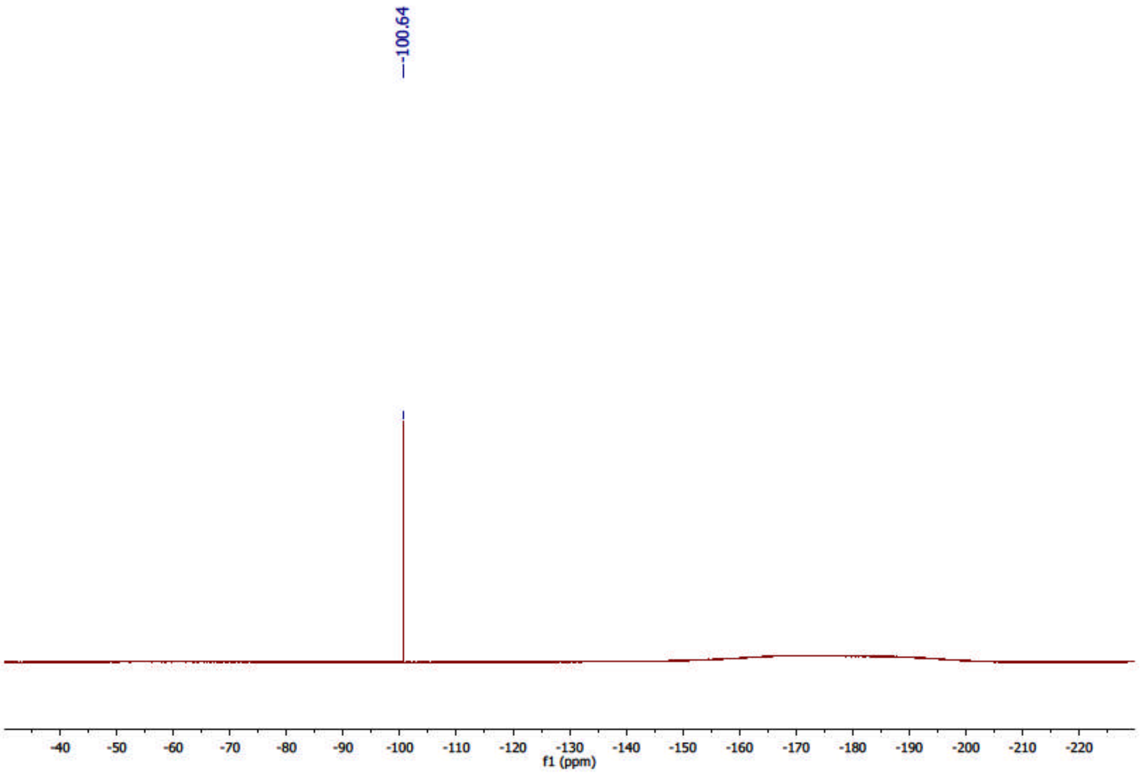

10c: ^{1}H NMR

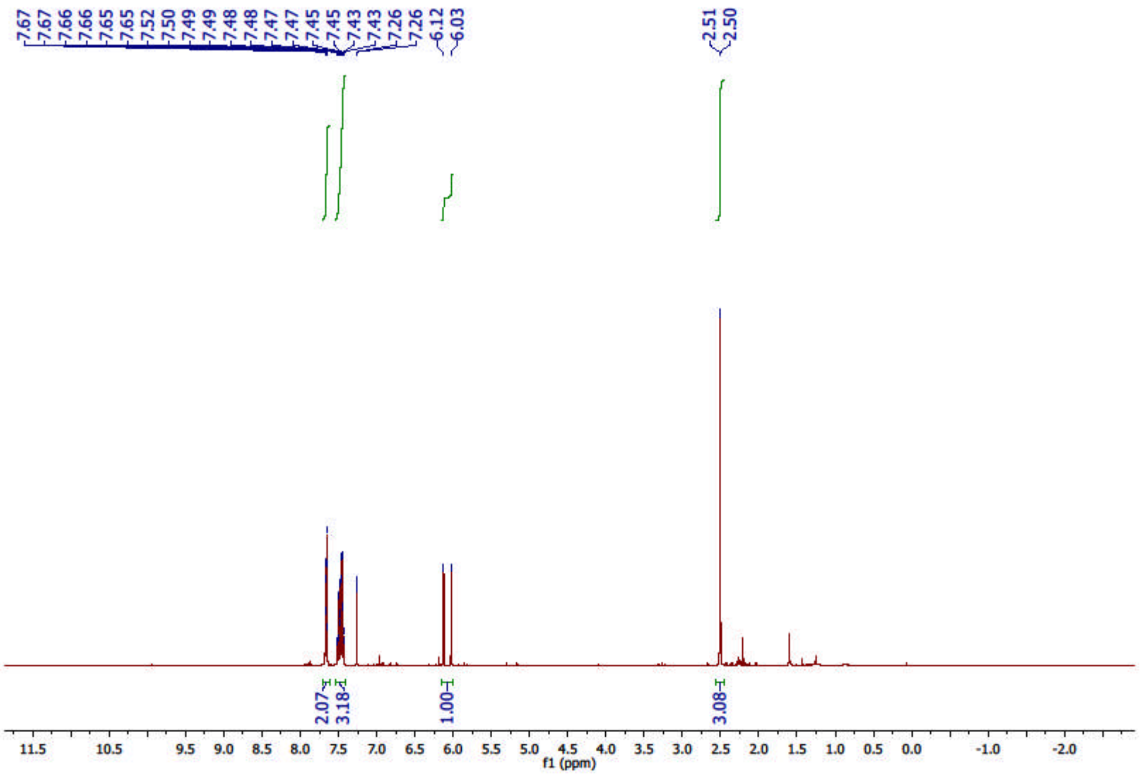

10c: ^{13}C NMR

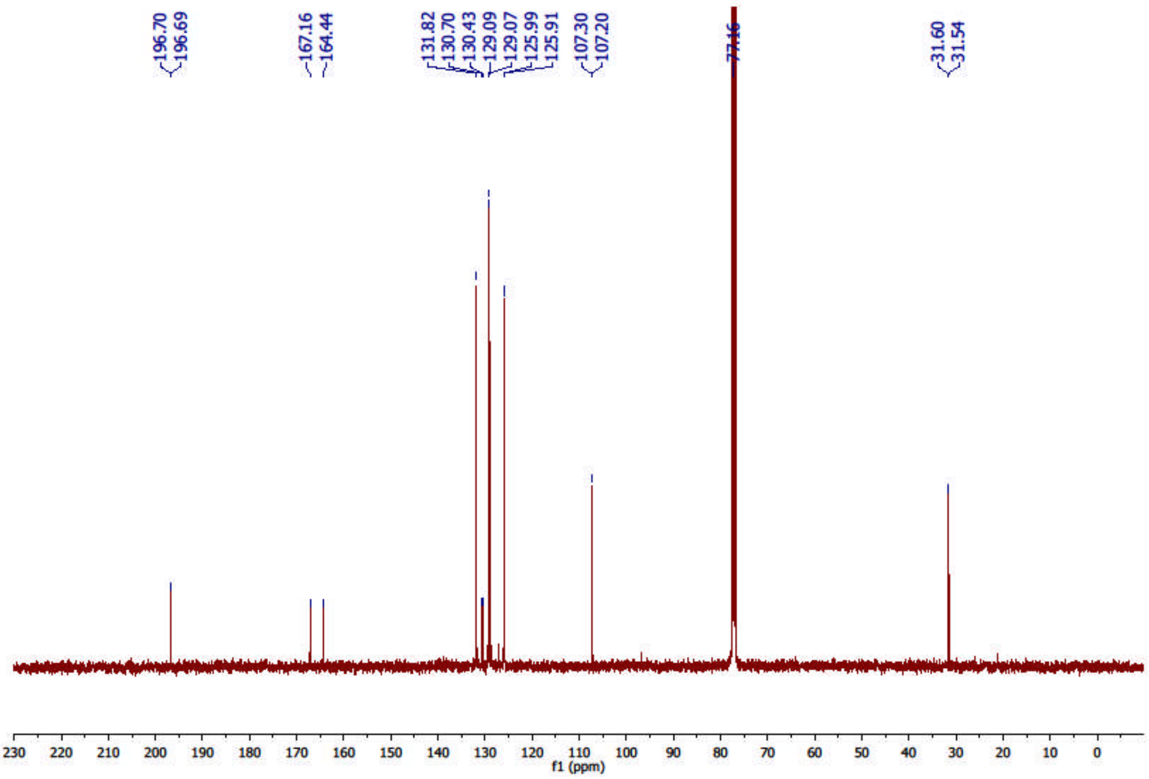

10c: ^{19}F NMR

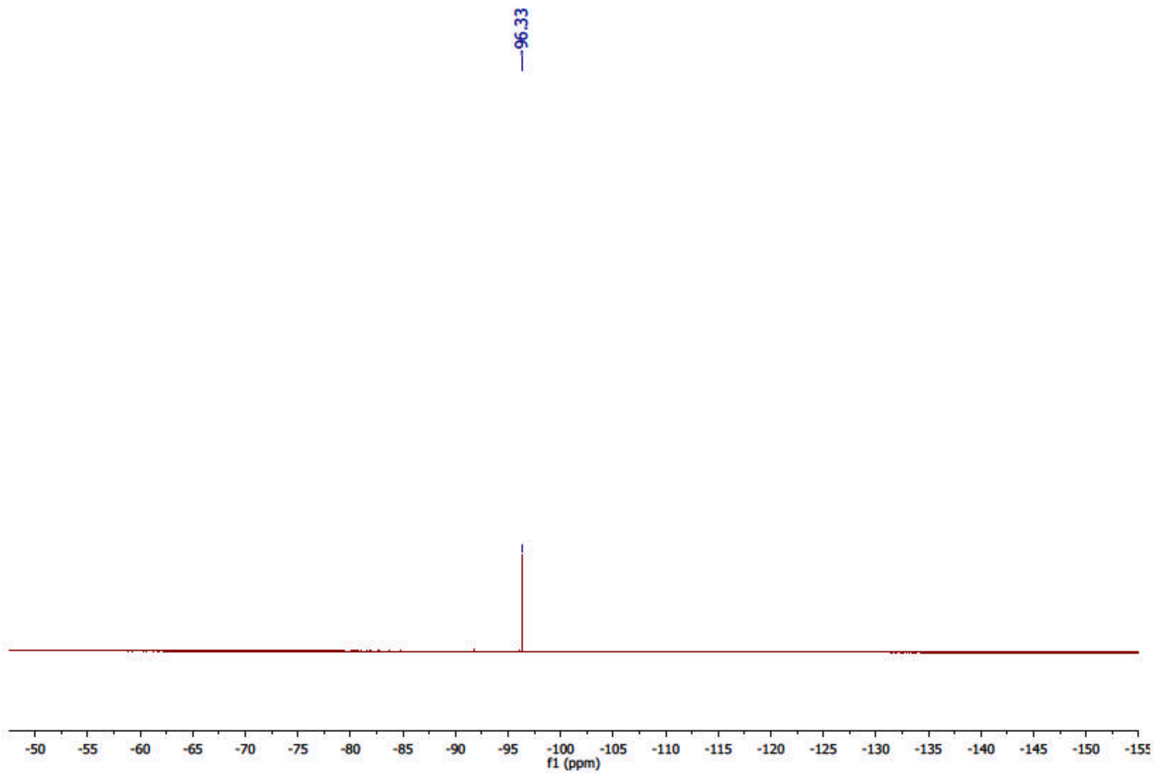

10d: ^{1}H NMR

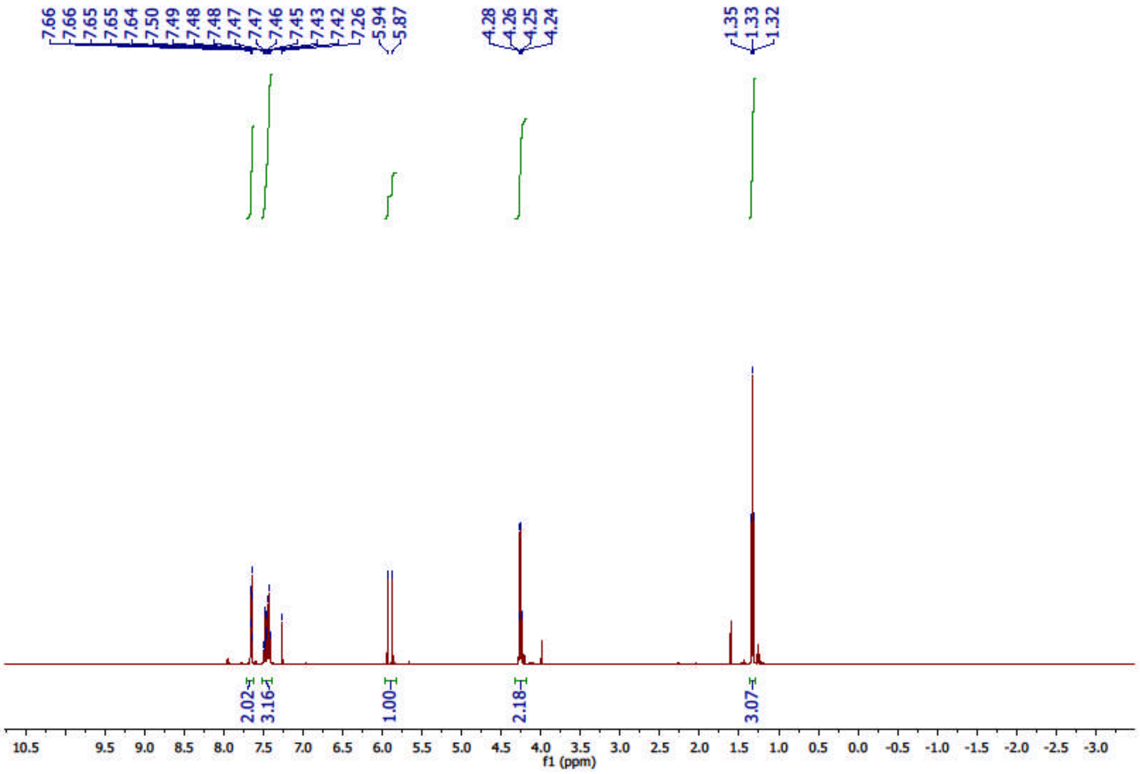

10d: ^{13}C NMR

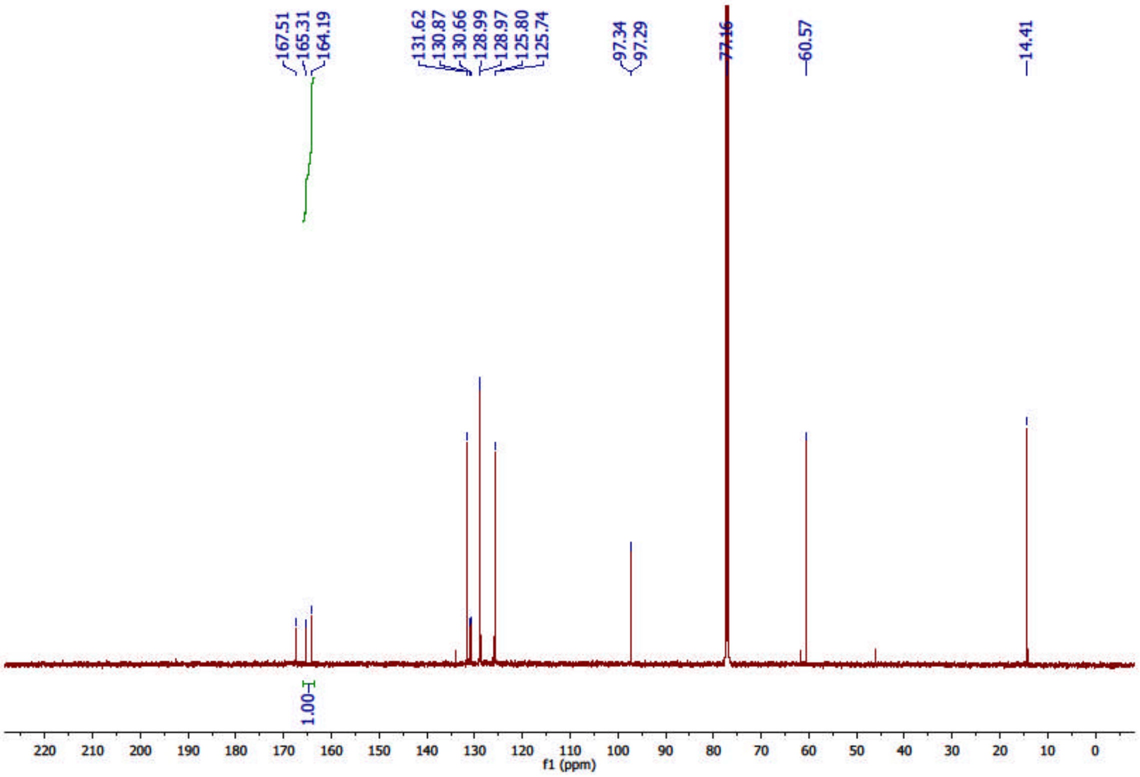

10d: ^{19}F NMR

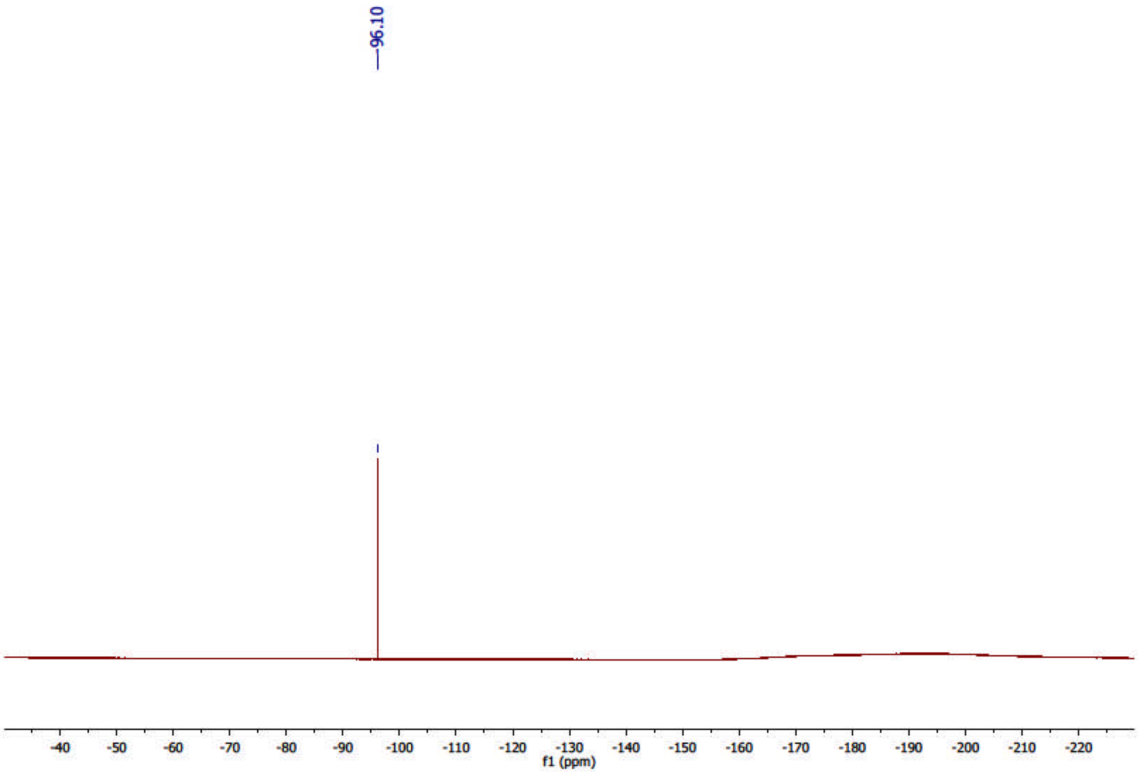

10e: ^{1}H NMR

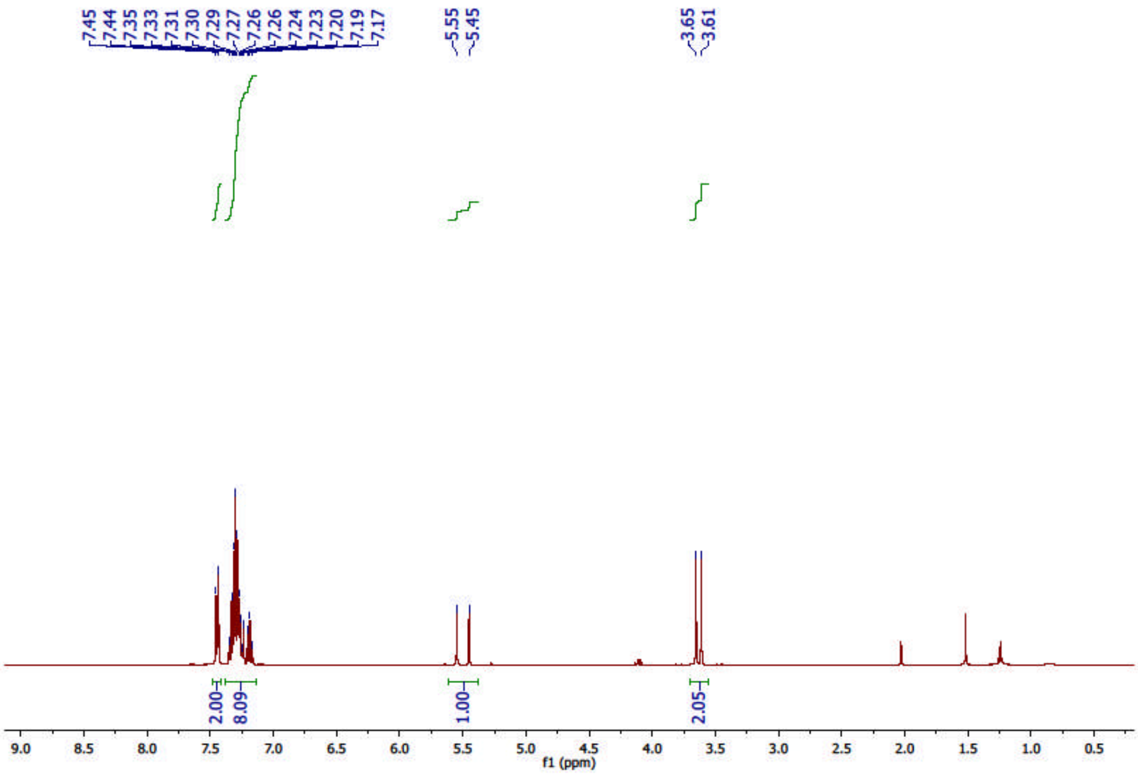

10e: ^{13}C NMR

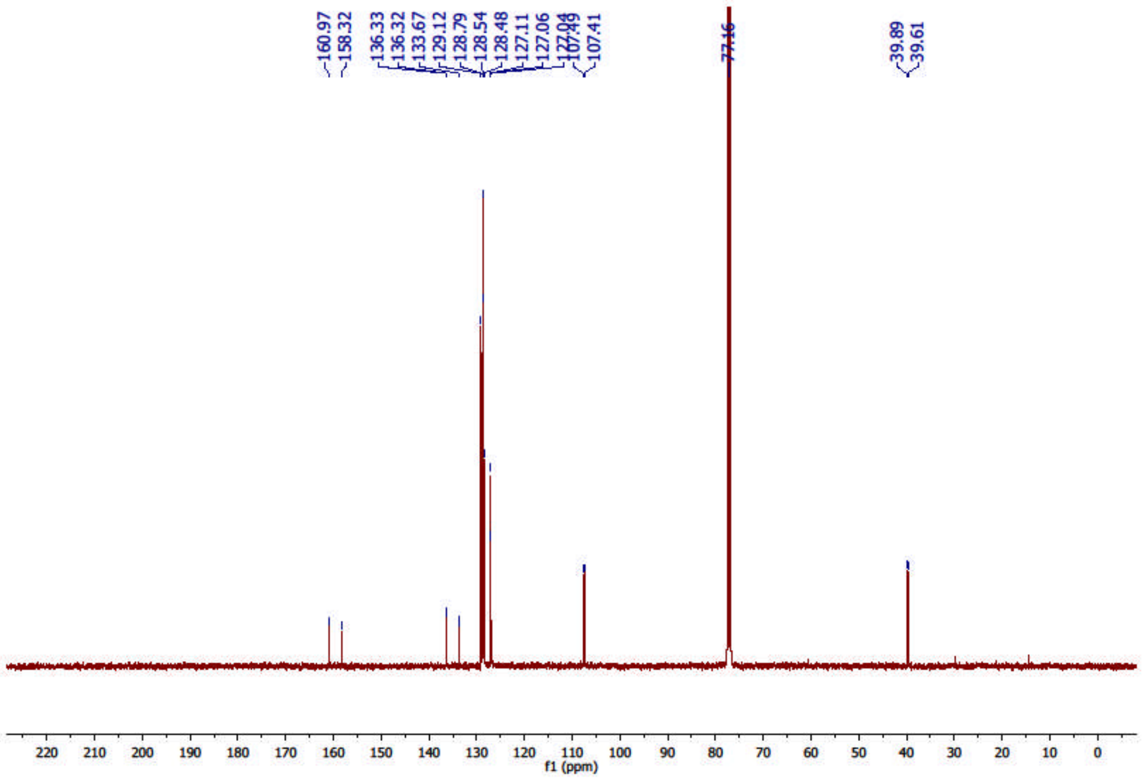

10e: ^{19}F NMR

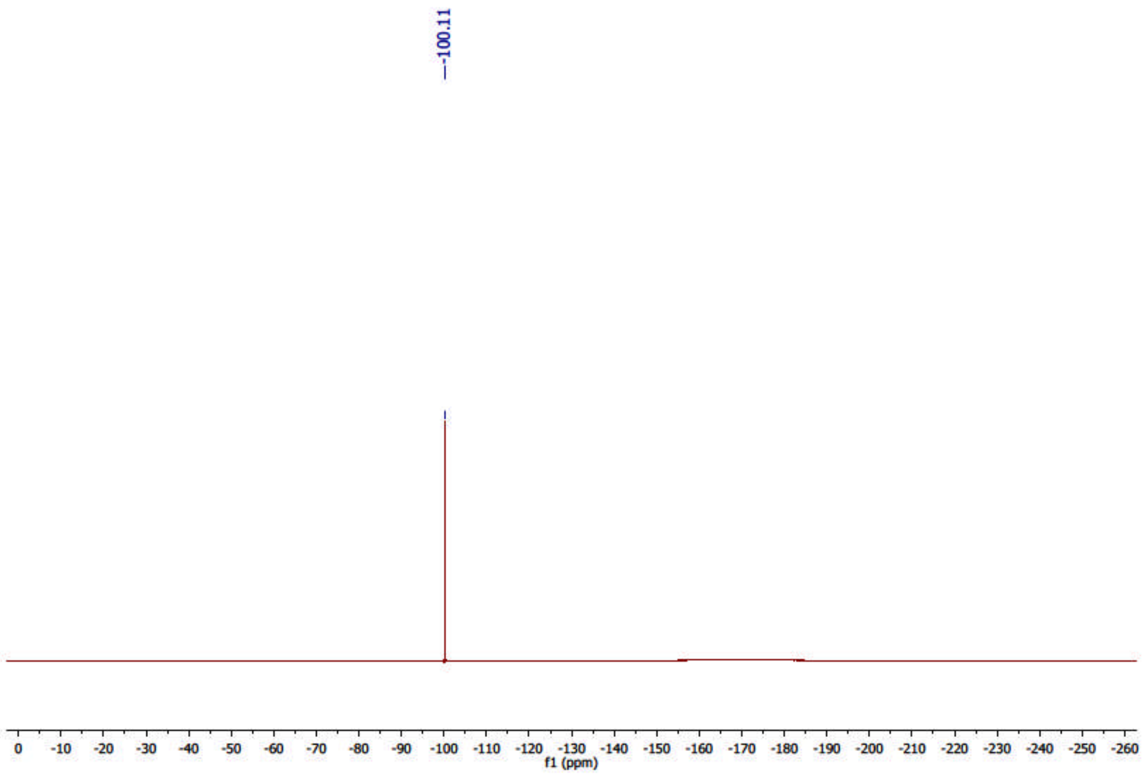

10f: ^{1}H NMR

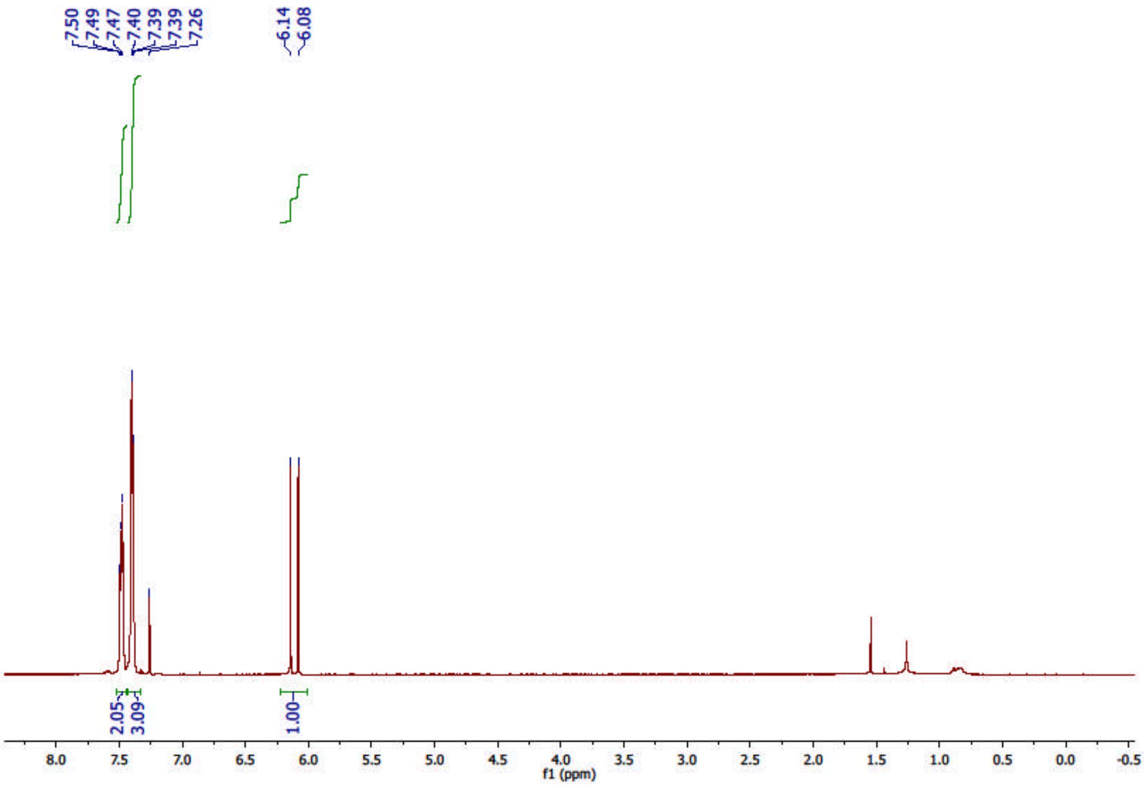

10f: ^{13}C NMR

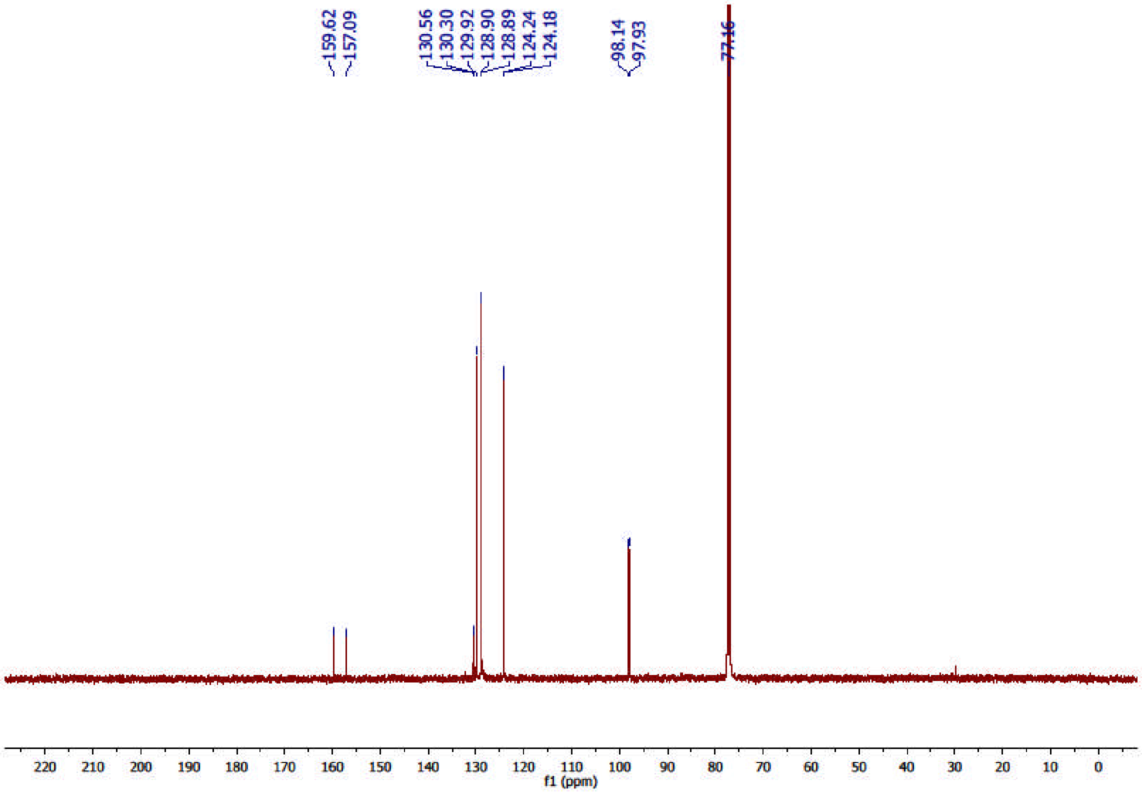

10f: ^{19}F NMR

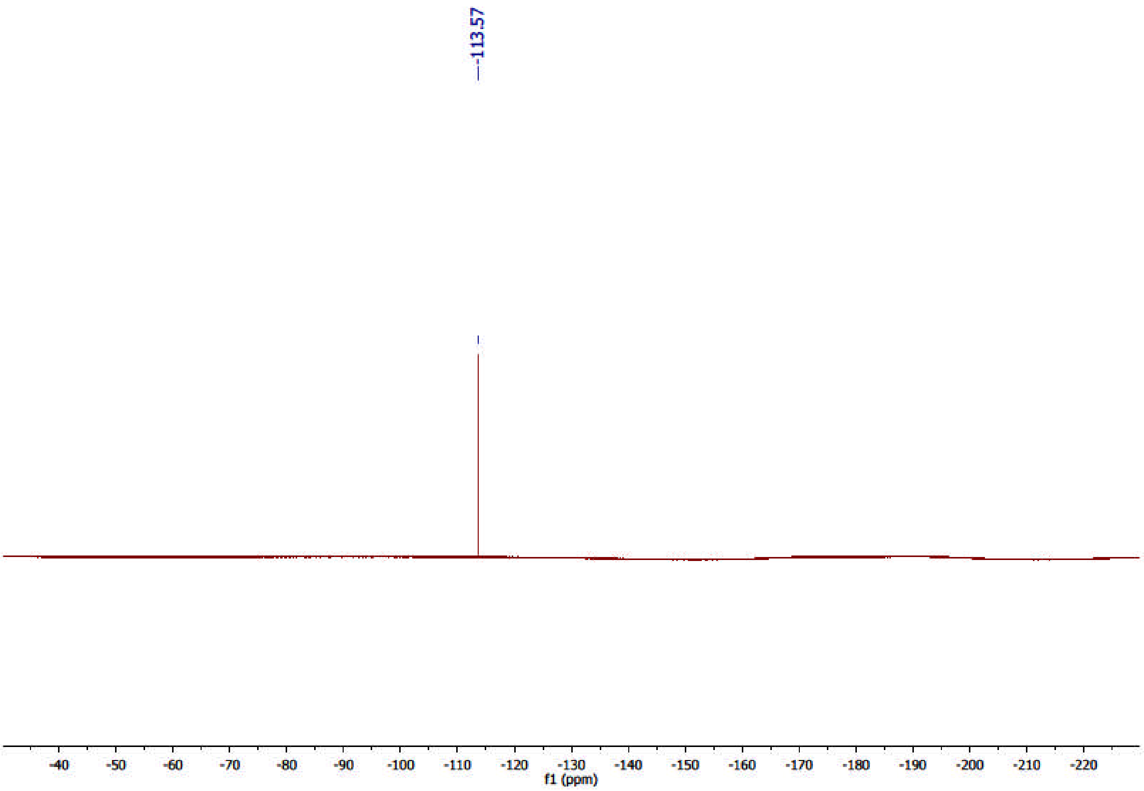

10g: ^{1}H NMR

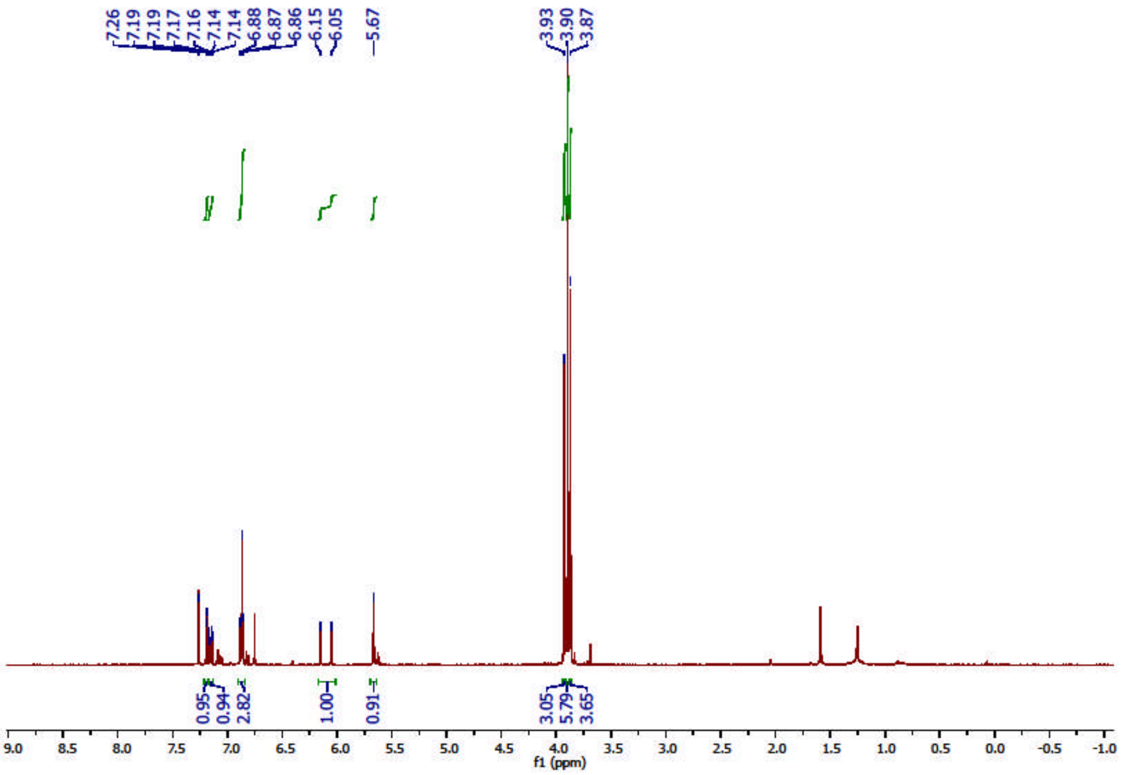

10g: ^{13}C NMR

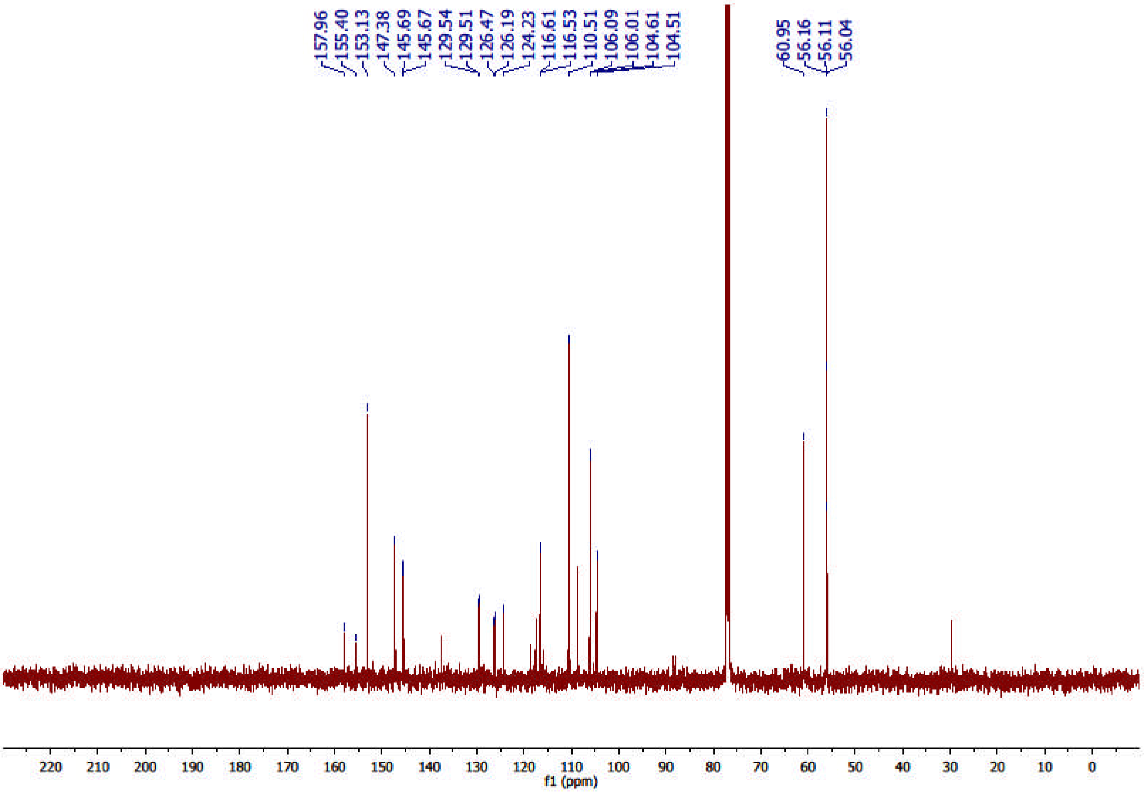

10g: ^{19}F NMR

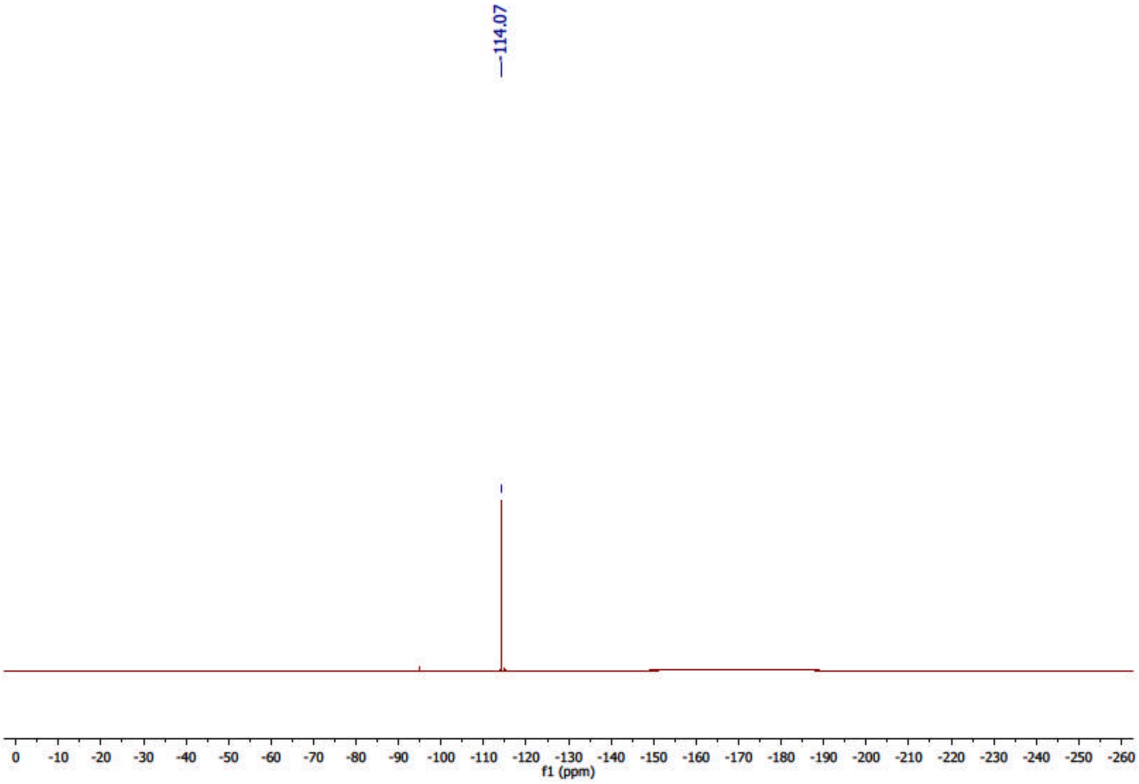

12a: ^{1}H NMR

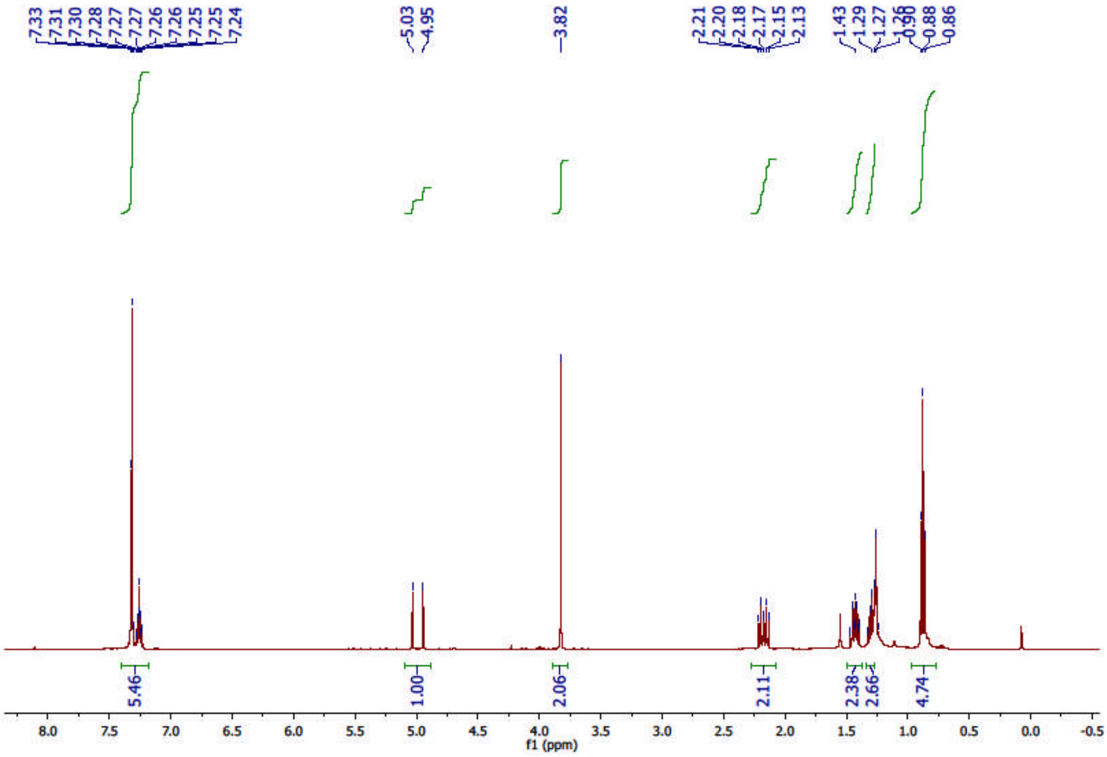

12a: ^{13}C NMR

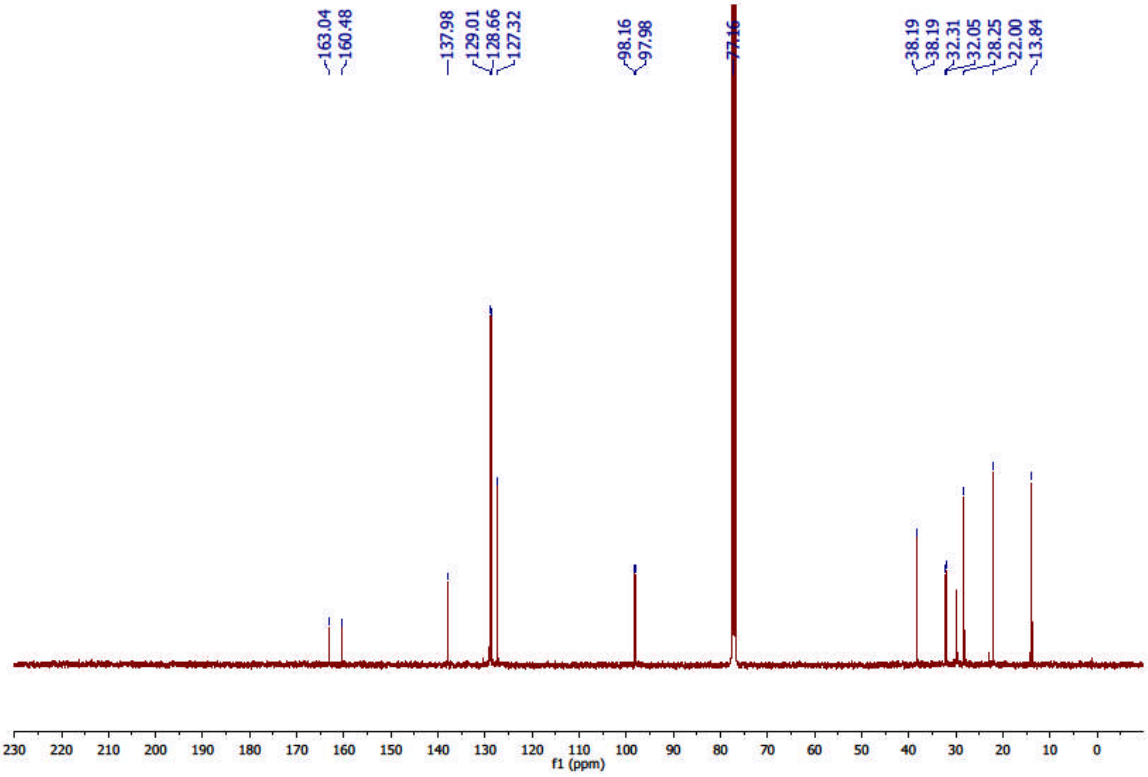

12a: ^{19}F NMR

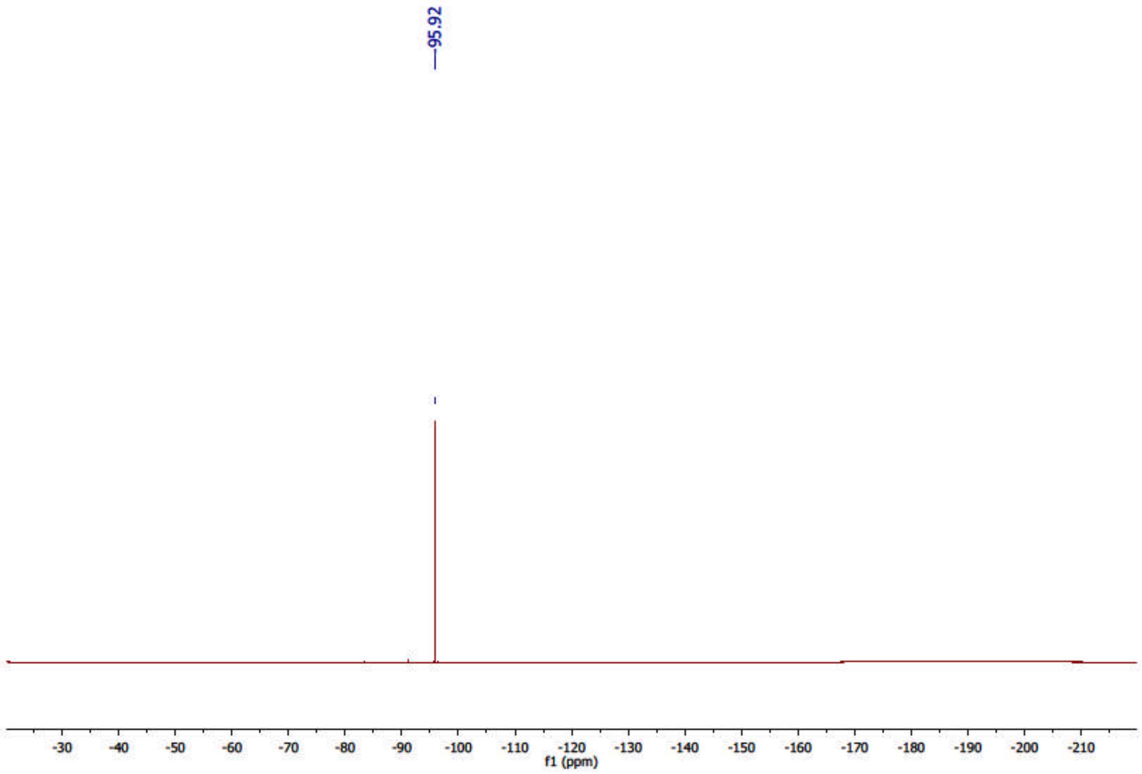

—95.92
-30
-40
-50
-60
-70
-80
-90
-100
-110
-120
-130
-140
-150
-160
-170
-180
-190
-200
-210
f1 (ppm)
